# Sm-site containing mRNAs can accept Sm-rings and are downregulated in Spinal Muscular Atrophy

**DOI:** 10.1093/nar/gkaf794

**Published:** 2025-08-18

**Authors:** Anton J Blatnik, Manu Sanjeev, Jacob Slivka, Benjamin Pastore, Caleb M Embree, Wen Tang, Guramrit Singh, Arthur H M Burghes

**Affiliations:** Department of Biological Chemistry and Pharmacology, The Ohio State University Wexner Medical Center, 395 W. 12th Ave, Columbus, OH 43210, United States; Department of Molecular Genetics, The Ohio State University, 484 West 12th Avenue, Columbus, OH 43210, United States; Department of Biological Chemistry and Pharmacology, The Ohio State University Wexner Medical Center, 395 W. 12th Ave, Columbus, OH 43210, United States; Department of Biological Chemistry and Pharmacology, The Ohio State University Wexner Medical Center, 395 W. 12th Ave, Columbus, OH 43210, United States; Center for RNA Biology, The Ohio State University, Columbus, OH 43210, United States; Department of Molecular Genetics, The Ohio State University, 484 West 12th Avenue, Columbus, OH 43210, United States; Department of Biological Chemistry and Pharmacology, The Ohio State University Wexner Medical Center, 395 W. 12th Ave, Columbus, OH 43210, United States; Center for RNA Biology, The Ohio State University, Columbus, OH 43210, United States; Department of Molecular Genetics, The Ohio State University, 484 West 12th Avenue, Columbus, OH 43210, United States; Center for RNA Biology, The Ohio State University, Columbus, OH 43210, United States; Department of Biological Chemistry and Pharmacology, The Ohio State University Wexner Medical Center, 395 W. 12th Ave, Columbus, OH 43210, United States; Center for RNA Biology, The Ohio State University, Columbus, OH 43210, United States

## Abstract

Sm-ring assembly is important for the biogenesis, stability, and function of uridine-rich small nuclear RNAs (U snRNAs) involved in pre-messenger RNA (mRNA) splicing and histone pre-mRNA processing. Sm-ring assembly is cytoplasmic and dependent upon the Sm-site sequence and structural motif, ATP, and *Survival motor neuron* (SMN) protein complex. While RNAs other than U snRNAs were previously shown to associate with Sm proteins, whether this association follows Sm-ring assembly requirements is unknown. We systematically identified Sm-sites within the human and mouse transcriptomes and assessed whether these sites can accept Sm-rings. In addition to snRNAs, Sm-sites are highly prevalent in the 3′ untranslated regions of long mRNAs. RNA immunoprecipitation experiments confirm that Sm-site containing mRNAs associate with Sm proteins in the cytoplasm. In modified Sm-ring assembly assays, Sm-site containing mRNAs, specifically associate with Sm proteins in an Sm-site, SMN, and ATP-dependent manner. In cell and animal models of Spinal Muscular Atrophy (SMA), mRNAs containing Sm-sites are downregulated, suggesting reduced Sm-ring assembly on these mRNAs may contribute to SMA pathogenesis. Together, this study establishes that Sm-site containing mRNAs can accept Sm-rings and identifies a novel mechanism for Sm proteins in regulation of cytoplasmic mRNAs.

## Graphical Abstract

**Graphical Abstract:**
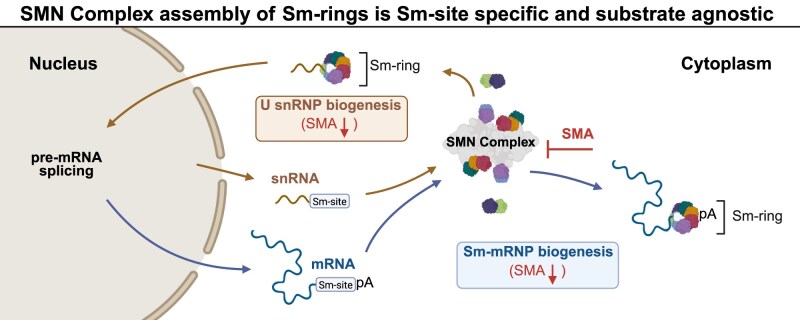

## Introduction

Sm-protein ring assembly is the process by which the seven Sm-proteins are assembled around a uridine-rich ribonucleic acid (RNA) sequence [1–6]. This action is essential for the biogenesis and stability of spliceosomal, uridine-rich small nuclear RNAs (U snRNA) that function as the catalytic subunits of the spliceosome [7, 8]. The Sm-ring stabilizes U snRNAs and provides a handle for components of the spliceosome to interact with the assembled U small nuclear RNP (snRNP) [9, 10]. While RNAs beyond U snRNAs have been proposed to interact with Sm-ring-like assemblies [11], the prevalence of Sm-ring assembly sites in eukaryotic genomes and the possibility of Sm-ring assembly on RNAs beyond U snRNAs remains largely unexplored.

Sm-ring assembly is a highly choreographed process involving several trans-acting protein members. The Sm-ring is comprised of seven proteins—SmB/B’, SmD1, SmD2, SmD3, SmE, SmF, and SmG—that are pre-assembled by the PRMT5 complex into SmB(B’)/D3, SmD1/D2, and SmF/E/G sub-complexes [5, 12–15]. These higher order Sm sub-complexes are bound by pICln, kinetically trapping the Sm-proteins from assembling nonspecifically on RNA [5, 15]. The higher-order pICln:Sm blocks are recruited to the SMN complex, composed of SMN, Gemins2-8, and Unrip, which specifically assembles the SmD1/D2/F/E/G subcore and then the SmB(B’)/D3 block to generate the full seven-member, heteromeric Sm-ring around a uridine-rich sequence motif within the U snRNA, known as an Sm-site [5, 6, 8, 12–27].

The Sm-site is classically defined as 5′-AUUUUU(U)G-3′, found in the U2, U4, U4atac, U5, U11, and U12 snRNAs [2, 4, 23, 28]. The Sm-site in U1 snRNA is distinct, in which the fourth uridine is replaced by a guanosine to yield 5′-AUUUGUG-3′. Mutation of the third uridine to a cytosine does not alter Sm-ring formation [23]. The U7 snRNA Sm-site is unique in that the sequence is longer —5′-AUUUGUCUAG-3′—, and results in the replacement of SmD1/D2 with Lsm10/11 [29]. This U7-specific hybrid Sm-ring is still assembled by the SMN complex, but results in a different protein association profile that facilitates the processing of histone pre-messenger RNAs (mRNAs) [22, 29, 30]. Apart from the uridine-rich sequence motif, the Sm-site includes a 3′ stem loop necessary for Sm-ring assembly [23, 28]. The Sm-ring forms a toroidal structure, in which individual bases directly interact with the individual members of the Sm-ring, making a core that is stable under high salt, heparin, and urea conditions [1–7, 12, 31]. Following Sm-ring assembly, U snRNA m^7^G-caps are hypermethylated (m^2,2,7^G), their 3′ ends are trimmed [26, 32–36] and they are imported into the nucleus through a mechanism involving snurportin-1 and importin-beta [26, 33, 34, 37–43]. Within the nucleus, nucleotides of U snRNPs are pseudouridylated or 2′-O-methylated [44, 45] and they associate with their intended RNA targets and protein effectors; pre-mRNA and the spliceosomal machinery in the case of spliceosomal U snRNPs, and histone pre-mRNA and processing factors for U7 snRNPs [46, 47].

Sm-ring assembly is an ATP, SMN, and Sm-site dependent process in cell extracts [21, 23, 48–50]. Though Sm-proteins will spontaneously associate with RNA when in high concentration, the PRMT5/SMN complex system inhibits this promiscuity in favor of the Sm-site [5, 12]. Sm-ring assembly requires the addition of ATP in cell and oocyte extracts [21, 49, 50]; however, reconstitution of ring assembly components *in vitro* indicate Sm-ring assembly is a Brownian activity involving a minimal SMN complex—SMN, Gemin2,6–8, Unrip, [15]. It was recently reported that the DEAD-box helicase, Gemin3, confers the requirement for ATP, unfolding U snRNA secondary structure that can shield the Sm-site [51]. Modification of the first and third uridine positions in the Sm-site, or removal of stem loop structures 3′ of the Sm-site sequence ablates Sm-ring assembly [23, 28]. Furthermore, SMN protein deficiency—causal of the human disease Spinal Muscular Atrophy (SMA)—results in a reduced capacity to assemble spliceosomal and U7 snRNPs, indicating Sm-ring assembly is SMN dependent [22, 30, 50, 52].

Reductions in SMN protein abundance have been shown to perturb several RNA processing events including transcription [53, 54], pre-mRNA splicing [55], U snRNP assembly [6, 16, 21, 30, 50, 56–60], histone pre-mRNA processing [22, 30], snoRNP assembly [43, 61–63], telomerase activity [43, 63], translation [64–67], signal recognition particle biogenesis [68], and mRNA trafficking [69–78]. However, a direct role for SMN in these events has not been forthcoming. A previous report indicated many types of RNAs associate with Sm proteins but did not establish that these RNAs could accept an Sm-ring [11]. Furthermore, Sm-ring assembly was found to be prerequisite to Lsm-ring assembly on the fission yeast telomerase RNA subunit (TER1) [79]. Given the short sequence conferring the Sm-site, we hypothesized that Sm-sites are present within many other RNAs beyond U snRNAs, that Sm-rings are assembled upon them, and this process may provide a direct link between accepted SMN function and the aforementioned RNA processing events. Our informatic search revealed that many human and mouse RNAs contain Sm-sites, but most notably, Sm-sites are enriched in mRNA 3′ untranslated regions (UTRs). To investigate whether prediction of an Sm-site informed enrichment upon Sm-protein immunoprecipitation, we modified U snRNP Sm-ring assembly assays to detect ring assembly on polyA-enriched RNA (polyA-RNA) using anti-Sm RNA immunoprecipitation and next-generation sequencing (RIP-Seq). We report that many Sm-site containing mRNAs associate with Sm-proteins and that Sm-ring assembly on these RNAs can occur in an ATP, SMN, and Sm-site specific manner. Lastly, Sm-site containing mRNAs are downregulated in models of SMA, potentially identifying a link between SMN and metabolism of novel Sm-site-containing mRNAs.

## Materials and methods

### Informatics pipeline to identify Sm-site containing RNAs

A miniconda3 environment was created to run SQlite3, SeqIO, pandas, numpy, and Bioconda packages Bio.Seq, and SeqUtils. A database was generated using SQlite3 from the NCBI Refseq and Gencode annotations of the human GRCh38 and mouse GRCm39 genomes that could easily recall gene ID, transcript ID, gene name, and coding sequence start and stop from the GFF files. Next, a python script was written to create a cursor that would search the supplied transcriptome fasta files for the presence of a U1 (5′-AUUUGUG-3′), U2 (5′-AUUUUUG-3′), U5 (5′-AUUUUUUG-3′), U7 (5′-AUUUGUCUAG-3′), or Noncanonical (5′-AUNUKUN-3′, where K is a G or U) sequence and capture the preceding 5′ 200 nucleotides proceeding 3′ 16–50 nucleotides into two separate output files. The aforementioned databases are used to populate information like the transcript ID, gene ID, and the region the Sm-site sequence was located within the transcript. This generates two output files for each Sm-site type, one for the preceding and one for the proceeding sequence. Next, these Sm-site output files were provided as input for RNAfold (ViennaRNA Package 2.0, version 2.6.4) [80] which predicts secondary structure for both the preceding and proceeding Sm-site sequences, resulting in a file providing the transcript ID, the queried sequence, and the structure prediction. A third python script was then used to determine if a secondary structure is predicted within 20 nucleotides 5′ or 10 nucleotides 3′ of the Sm-site using recognition. This script then discards all identified Sm-sites that are not flanked by secondary structures on both sides. This yields a single output file for each type of Sm-site identified with the associated transcript ID, gene name, the region the Sm-site is found in, and the proceeding 3′ sequence used in structure prediction. These output files are supplied in Supplementary File 1 and were then used to generate all following master tables (Supplementary File 2). The python scripts used to generate this data are available in Github (https://github.com/ajblatnik/sm_ring_assembly_mrna.git) and Zenodo (DOI:10.5281/zenodo.15476098).

For the figures displayed within the study, frequency of Sm-sites is given for different regions and different transcripts on a gene-level basis. In this, each gene ID corresponds to a single transcript ID, and only Sm-sites identified for that transcript ID were used from the output files. To facilitate this, R (version 4.4.1) was used to cross-reference the shared and annotation-specific Sm-sites identified in the Refseq and Gencode Sm-site output files in Supplementary File 1, by first retaining those transcript IDs that have shared proceeding 3′ sequence and then appending sites specific to Refseq or Gencode. The frequency of Sm-sites identified for each transcript and transcript region was calculated and a master table was generated by inner-joining with a list of gene IDs pared down to a single transcript ID, prioritizing by MANE and Ensembl Transcript is Canonical notation as provided in BiomaRt [81, 82]. This was repeated for each type (U1, U2, U5, U7, and Noncanonical) to generate a single table that contained all information. Given that U1, U2, and U5 Sm-site types will satisfy the Noncanonical arguments, the frequency of true Noncanonical Sm-sites was determined by subtracting the frequency of noncanonical Sm-sites from the raw output by the number of U1, U2, and U5 Sm-site types. Canonical Sm-site frequencies were determined by adding the frequencies for U1, U2, and U5 Sm-site types. Transcript, 5′UTR, CDS, and 3′UTR lengths were then populated into these master tables which are available as Supplementary File 2. The R scripts and session info used to generate these tables are available in Github (https://github.com/ajblatnik/sm_ring_assembly_mrna.git) and Zenodo (DOI:10.5281/zenodo.15476098). These tables were then used to generate all the data and graphs within the study.

### Informatics pipeline to identify splice-site sequence containing RNAs

The python scripts used to identify RNAs containing Sm-sites was modified to find sequences matching 5′ splice-site (5′ss) sequences and branch-points (Bpt). 5′ss were defined as 5′-MAGGURAGK (where M: A/C, R: A/G, and K: G/T). Provided known 5′ss can incorporate a mismatch (snRNA side) or deletion (within the splice-site), this query was opened to include either a single mismatch or deletion—5′ss w/ bulge. Branch-points were defined in two manners: the degenerate Consensus sequence (5′-YUNAYYYY, where Y is C/T, and N is A/T/C/G) or a more Stringent U1/U12 match (5′-YMYUNACW, where Y: C/T, M: A/C, W: A/T). Queries were performed in both NCBI Refseq and Gencode annotations of the human GRCh38 and mouse GRCm39 transcriptomes and matches were cross-referenced between both in R to provide a single match file for each query (Supplementary File 1). The presence of these sequences was annotated into the Sm-site master tables in Supplementary File 2. The python code, R scripts, and tables are available in Github (https://github.com/ajblatnik/sm_ring_assembly_mrna.git) and Zenodo (DOI:10.5281/zenodo.15476098). These tables were then used to generate all the data and graphs within the study.

### Informatics pipeline to assign counts to only intronic sequences

A Simplified Annotation File (SAF) was generated from the Gencode human GRCh38 and mouse GRCm39 genomes using the GRanges R package and using setdiff to subtract transcript coordinates from the genome. This intron SAF was supplied to featureCounts, along with the appropriate human or mouse transcript GTF and STAR supplied BAM files to assign counts only to intronic regions. The featureCounts outputs were then processed in R to obtain a list of all genes in which a read was assigned within an intron for the transcript. This list was used to annotate the Sm-site master tables in Supplementary File 2. The python code and R scripts and tables are available in Github (https://github.com/ajblatnik/sm_ring_assembly_mrna.git) and Zenodo (DOI:10.5281/zenodo.15476098). These tables were then used to generate all the data and graphs within the study.

### Cell culture

Neural stem cell NSC-34 cells were cultured in 12% Fetal Bovine Serum (FBS) Dulbecco’s modified Eagle’s medium (DMEM) supplemented with L-glutamine and PenStrep [12% FBS (Atlanta S11550), 10% PenStrep/L-Glutamine (Gibco 10378-016), DMEM (Gibco 11960077)] under 37°C, 5% CO_2_ in normoxic conditions. Cells were routinely passaged every 2–3 days by washing twice with phosphate-buffered saline (PBS) lacking divalent cations, and then treating with 0.05% Trypsin–ethylenediaminetetraacetic acid (EDTA) (Gibco 25300054). S3 fibroblasts were induced to a neural progenitor cell fate as described by Meyer *et al.* [83]. To culture neural progenitor cell S3-induced neural progenitor cells (iNPCs), dishes were coated with fibronectin (Millipore FC010-10MG) and cells were cultured in DMEM/F12 + Glutamax (Gibco 10565-042) supplemented with 1% N2 (Gibco 17502-048), 1% B27 (Gibco 17504-044), 1% Anti-Anti (Gibco 15270-62), and 20 ng/ml FGF2 (Peprotech 100-18B), under 37°C, 5% CO_2_ in normoxic conditions. Cells were routinely passaged every 2–3 days by washing twice with PBS lacking divalent cations and treating with StemPro Accutase (Thermo Fisher A1110501).

### Oligo-dT isolation of polyA-RNA

Four 15-cm dishes of NSC-34 or S3-iNPC cell were washed three times with PBS lacking divalent cations and treated with 8-ml TRIzol Reagent (Invitrogen 15596026) per 15-cm dish. TRIzol samples were pooled and a large scale total RNA isolation and precipitation was performed following manufacturer’s instructions. After purification, isolated RNA was treated with 200 units of Turbo DNase (Invitrogen AM2239) and incubated at 37°C for 30 min with gentle rotation at 350 rpm in Eppendorf Thermomixer R. DNase was removed by phenol:chloroform extraction. RNA was precipitated with 1.5-M final concentration of LiCl and 100% ethanol, centrifuged at >18 000 × *g* for 15 min. RNA was resuspended in 1 ml nuclease-free water and quantified using Qubit RNA Broad Range Assay (Invitrogen Q10211). PolyA-RNA was isolated using Dynabeads mRNA Purification Kit (Invitrogen 61006). Oligo-dT beads were reused post-polyA-RNA elution to isolate the magnitude of polyA-RNA required for modified Sm-ring assembly reaction and sequencing conditions. After isolation, polyA-RNA was quantified using Qubit RNA Broad Range Assay (Invitrogen Q10211) and RNA integrity (RIN) was quantified by TapeStation analysis performed by the Ohio State Comprehensive Cancer Center Genomics Core to determine removal of polyA-minus RNAs (Supplementary Fig. S5C).

### Sm-ring assembly assays

Preparation of cytoplasmic cell extracts and standard Sm-ring assembly assays were performed as previously described in Wan *et al.* (2005), and Blatnik *et al.* (2020) [50, 84]. Cytoplasmic cell extracts were generated by scraping and collecting NSC-34 or S3-iNPC cells in cold PBS containing only monovalent cations. Cells were pelleted by centrifugation 400 × *g* for 4 min. Cells were resuspended in digitoxin lysis buffer (20-mM Tris, pH 8, 50-mM KCl, 0.5 MgCl_2_, 1× eComplete, 200 units/ml RNase-OUT, 50-μg/ml digitonin, in diethyl pyrocarbonate (DEPC)-treated water) and incubated on ice for a few minutes. Lysates were passaged six times through a 27 gauge needle with syringe then centrifuged at 1500 × *g* for 1 min at 4°C. Supernatant was measured and transferred to a new microfuge tube and supplemented with NP-40 to achieve 0.01% final concentration. Lysates were then centrifuged at 4000 × *g* for 15 min at 4°C. Supernatants were then aliquoted into microfuge tubes and frozen in liquid nitrogen. Protein concentrations were determined by bicinchoninic acid (BCA) assay (Pierce 23250). Protein-A DynaBeads (Invitrogen 10001D) were aliquoted 8.6 μl/rxn and washed three times in PBS + 0.1% NP-40. 0–3.2 μg/rxn Y12 antibody (Novus Biologicals NB600-546, EMD Millipore MABF2793, Invitrogen MA5-13449) or SmB/B’/N (12F5) (Santa Cruz sc-130670) was conjugated to Protein-A DynaBeads by incubating in PBS + 0.1% NP-40 for 1 h at 4°C with rotation; 1.6 μg/rxn Novus Biologicals NB600-546 Y12 antibody was used for all subsequent experiments. Beads were washed three more times with PBS + 0.1% NP-40, then three times with RSB-500 + 0.1% NP-40 (RSB-500: 10-mM Tris, pH 7.4, 500-mM NaCl, 2.5 mM MgCl_2_). Beads were finally resuspended in 120-μl/rxn RSB-500 + 0.1% NP-40 supplemented with 2-mg/ml heparin. Sm-ring assembly was performed in 20-μl total volume and incubated for 1 h at 30°C with shake at 750 rpm (1 unit/μl RNAse-OUT, 0.25-μg/μl yeast transfer RNA, 0.01% NP-40, 2.5-mM ATP, 20-mM Tris, pH 8, 50-mM KCl, 0.5-MgCl_2_, 25-μg cytoplasmic extract, and 10-nM biotinylated U4, U4ΔSm snRNA, mRNA, or mRNA 3′UTR). Assembly reactions were immunoprecipitated with 120-μl/rxn Y12-conjugated dynabeads, incubated for 1 h at 30°C with shaking at 750 rpm. Beads were washed eight times with RSB-500 + 0.1% NP-40, utilizing magnets to sequester beads. Assembled snRNPs were detected by incubating the reactions with 120-μl RSB-500 + 0.1% NP-40 supplemented with 1:10 000 dilution of NeutrAvidin-horseradish peroxidase (HRP) (Invitrogen A2664) for 1 h at 30°C with shake at 750 rpm. Beads were further washed eight times with RSB-500 + 0.1% NP-40, utilizing magnets to sequester beads. Reactions were finally resuspended in 150 μl of 1:1 mixture of SuperSignal Femto ELISA substrate (Pierce 37075), transferred to Nunclon white-bottom plate (Nunclon Delta Surface 136101). Luminescence was measured on Tecan Infinite F200 using I-Control software or Promega GloMAX plate reader, no attenuation, 1000-ms integration, 0-ms settle. Raw luminescence values were plotted for +ATP, −ATP, ΔSm + ATP, and ΔSm − ATP conditions. Graphs were plotted using GraphPad Prism Version 10.2.3 (347). For testing Sm-ring assembly directly on mRNA and mRNA 3′UTRs, prior to incubation with Y12 antibody, reactions were supplemented with 2-M urea and 5 mg/ml heparin and incubated for 15 min at 30°C.

For modified Sm-ring assembly reactions, reactions were scaled up to 250-μg human S3-iNPC and 438-μg mouse NSC-34 total protein cytoplasmic extract, supplemented with 4 μg polyA-RNA (RIN 5–6; Supplementary Fig. S5C) to capture sufficient RNA for sequencing library generation; 10 nM U4 snRNA is routinely used to perform assembly reactions in 25 μg total protein cytoplasmic lysate, however human U4 snRNA is only 141 nt in length. Therefore, ∼18 nM polyA-RNA was used as input for the assembly reactions, assuming an average mRNA length of ∼3400 nt in humans and ∼3200 nt in mouse. Three replicates of the four conditions presented in Supplementary Fig. S5B for both species combinations were used to generate sequencing libraries, using 50 ng of captured RNA as input for the Takara SMARTer Stranded Total RNA-Seq Kit v3-Pico Input Mammalian (Takara 634451), and sequenced using the NOVA-Seq platform, 150 base-pair paired-end reads. The four conditions are: (i) the polyA-RNA input used for the assembly reactions, (ii) anti-Sm-RIP of the cytoplasmic cell extract, (iii) anti-Sm-RIP of cell extracts incubated with polyA-RNA, and (iv) anti-Sm-RIP of cell extracts incubated with polyA-RNA and ATP but are also described in Supplementary Fig. S4B. For sequencing experiments in Figs 5 and 6 and Supplemental Figs S19–S21, extracts and/or reactions were supplemented with 2-M urea and 5 mg/ml heparin and incubated for 15 min at 30°C prior to Sm-antibody capture in RSB-500 + 0.1% NP-40 supplemented with 2-mg/ml heparin. Eight washes were performed using RSB-1000 + 0.1% NP-40 (RSB-1000: 10-mM Tris, pH 7.4, 1-M NaCl, 2.5-mM MgCl_2_).

### Informatic analysis of sequencing data

*For sequencing experiments discussed in* *Supplementary Fig. S4B:* Table 1 lists the sequencing data generated in this study. Adapter sequences were trimmed using Cutadapt. Reads were aligned to both the human GRCh38 and mouse GRCm39 genome assemblies (Ensembl release 100) using STAR [85]. Reads were counted using FeatureCounts from the subread package, using the fractional (—*fraction*) argument for reads mapped to multiple loci. Bash scripts used to process reads and MultiQC [86] html are provided to assess quality of reads, alignment, and features in Github (https://github.com/ajblatnik/sm_ring_assembly_mrna.git) and Zenodo (DOI:10.5281/zenodo.15476098), and percent uniquely mapped reads for alignment are provided in Supplementary Fig. S5D. Wald-test comparisons of the FeatureCounts [87] outputs were performed using Deseq2 [88] in R (version 4.4.1). For all comparisons, the adjusted *P-*values from Deseq2 were used. Comparisons performed are discussed in Supplementary Fig. S4B and Figs 2A and 3A. Principal component analysis was performed on the top 1000 genes to ensure proper clustering of samples (Supplementary Fig. S5E). Custom R scripts were then written to analyze the data presented within this manuscript and the R session info are available in Github (https://github.com/ajblatnik/sm_ring_assembly_mrna.git) or Zenodo (DOI:10.5281/zenodo.15476098).

**Table 1.** Sm-RIP sequencing data generated for this study (GSE278538)

SRA RunID	Condition	Sample shorthand
GSM8548423	Human polyA-RNA 1	hr1
GSM8548424	Human polyA-RNA 2	hr2
GSM8548425	Human polyA-RNA 3	hr3
GSM8548426	Mouse polyA-RNA 1	mr1
GSM8548427	Mouse polyA-RNA 2	mr2
GSM8548428	Mouse polyA-RNA 3	mr3
GSM8548429	Human Sm-RIP 1	hx1
GSM8548430	Human Sm-RIP 2	hx2
GSM8548431	Human Sm-RIP 3	hx3
GSM8548432	Mouse Sm-RIP 1	mx1
GSM8548433	Mouse Sm-RIP 2	mx2
GSM8548434	Mouse Sm-RIP 3	mx3
GSM8548435	Modified Sm-RIP (human extract, mouse polyA-RNA) 1	hxmr1
GSM8548436	Modified Sm-RIP (human extract, mouse polyA-RNA) 2	hxmr2
GSM8548437	Modified Sm-RIP (human extract, mouse polyA-RNA) 3	hxmr3
GSM8548438	Modified Sm-RIP (mouse extract, human polyA-RNA) 1	mxhr1
GSM8548439	Modified Sm-RIP (mouse extract, human polyA-RNA) 2	mxhr2
GSM8548440	Modified Sm-RIP (mouse extract, human polyA-RNA) 3	mxhr3
GSM8548441	Modified Sm-RIP (human extract, mouse polyA-RNA, +ATP) 1	hxmra1
GSM8548442	Modified Sm-RIP (human extract, mouse polyA-RNA, +ATP) 2	hxmra2
GSM8548443	Modified Sm-RIP (human extract, mouse polyA-RNA, +ATP) 3	hxmra3
GSM8548444	Modified Sm-RIP (mouse extract, human polyA-RNA, +ATP) 1	mxhra1
GSM8548445	Modified Sm-RIP (mouse extract, human polyA-RNA, +ATP) 2	mxhra2
GSM8548446	Modified Sm-RIP (mouse extract, human polyA-RNA, +ATP) 3	mxhra3
GSM8974308	Human cytoplasmic RNA 1	hc1
GSM8974309	Human cytoplasmic RNA 2	hc2
GSM8974310	Human cytoplasmic RNA 3	hc3
GSM8974311	Human SmB Sm-RIP, stringent washing 1	hbs1
GSM8974312	Human SmB Sm-RIP, stringent washing 2	hbs2
GSM8974313	Human SmB Sm-RIP, stringent washing 3	hbs3
GSM8974314	Human Y12 Sm-RIP, stringent washing 1	hys1
GSM8974315	Human Y12 Sm-RIP, stringent washing 2	hys2
GSM8974316	Human Y12 Sm-RIP, stringent washing 3	hys3
GSM8974317	Modified Sm-RIP (human extract, mouse polyA-RNA, +ATP, stringent washing) 1	hxmrs1
GSM8974318	Modified Sm-RIP (human extract, mouse polyA-RNA, +ATP, stringent washing) 2	hxmrs2
GSM8974319	Modified Sm-RIP (human extract, mouse polyA-RNA, +ATP, stringent washing) 3	hxmrs3
GSM8974320	Modified Sm-RIP (mouse extract, human polyA-RNA, +ATP, stringent washing) 1	hxmras1
GSM8974321	Modified Sm-RIP (mouse extract, human polyA-RNA, +ATP, stringent washing) 2	hxmras2
GSM8974322	Modified Sm-RIP (mouse extract, human polyA-RNA, +ATP, stringent washing) 3	hxmras3

*For analyses of sequences discussed in* *Figs 5 and* *6:* Table 2 lists the publicly available sequencing data analyzed in the study. Data for each sample were retrieved from the NCBI Short Read Archive using their corresponding RunIDs with the fastqdump function of the SRA toolkit. The downloaded libraries were free from significant adapter content, eliminating the need for trimming. Sequencing reads were aligned to the genome (GRCh38/GRCm39, Ensembl release100) utilizing the STAR aligner [85]. The aligned reads were then quantified using FeatureCounts [87] from the subread package. Differential expression was calculated for SMN deficient conditions by running DESeq2 [88] on the Featurecounts output. For each study, principal component analysis was performed with 2000 genes showing most variance to confirm that samples clustered by disease condition. Genes without a calculated *padj* value after DESeq2 were discarded. The cumulative distribution of log_2_ fold-changes was plotted after grouping genes based on the presence/absence of a canonical Sm-site.

**Table 2.** Publicly available sequencing data analyzed in this study

SRA RunID	Genotype	Sample name	Sample type	Species	Study
ERR922474	Heterozygous	Spinal_cord_PND5_Het_10	Spinal cord	Mus musculus	Doktor *et al.*
ERR922475	Heterozygous	Spinal_cord_PND5_Het_4	Spinal cord	Mus musculus	
ERR922476	Heterozygous	Spinal_cord_PND5_Het_6	Spinal cord	Mus musculus	
ERR922477	Heterozygous	Spinal_cord_PND5_Het_7	Spinal cord	Mus musculus	
ERR922478	Spinal muscular atrophy	Spinal_cord_PND5_SMA_1	Spinal cord	Mus musculus	
ERR922479	Spinal muscular atrophy	Spinal_cord_PND5_SMA_13	Spinal cord	Mus musculus	
ERR922480	Spinal muscular atrophy	Spinal_cord_PND5_SMA_14	Spinal cord	Mus musculus	
ERR922481	Spinal muscular atrophy	Spinal_cord_PND5_SMA_5	Spinal cord	Mus musculus	
ERR922458	Heterozygous	Muscle_PND5_Het_10	Muscle	Mus musculus	
ERR922459	Heterozygous	Muscle_PND5_Het_4	Muscle	Mus musculus	
ERR922460	Heterozygous	Muscle_PND5_Het_6	Muscle	Mus musculus	
ERR922461	Heterozygous	Muscle_PND5_Het_7	Muscle	Mus musculus	
ERR922462	Spinal muscular atrophy	Muscle_PND5_SMA_1	Muscle	Mus musculus	
ERR922463	Spinal muscular atrophy	Muscle_PND5_SMA_2	Muscle	Mus musculus	
ERR922464	Spinal muscular atrophy	Muscle_PND5_SMA_5	Muscle	Mus musculus	
ERR922465	Spinal muscular atrophy	Muscle_PND5_SMA_9	Muscle	Mus musculus	
ERR922442	Heterozygous	Liver_PND5_Het_10	Liver	Mus musculus	
ERR922443	Heterozygous	Liver_PND5_Het_4	Liver	Mus musculus	
ERR922444	Heterozygous	Liver_PND5_Het_6	Liver	Mus musculus	
ERR922445	Heterozygous	Liver_PND5_Het_7	Liver	Mus musculus	
ERR922446	Spinal muscular atrophy	Liver_PND5_SMA_1	Liver	Mus musculus	
ERR922447	Spinal muscular atrophy	Liver_PND5_SMA_13	Liver	Mus musculus	
ERR922448	Spinal muscular atrophy	Liver_PND5_SMA_14	Liver	Mus musculus	
ERR922449	Spinal muscular atrophy	Liver_PND5_SMA_5	Liver	Mus musculus	
ERR1547208	Adenocarcinoma	HeLa_NT_1_p	HeLa cell culture	Homo sapiens	
ERR1547209	Adenocarcinoma	HeLa_NT_2_p	HeLa cell culture	Homo sapiens	
ERR1547210	Adenocarcinoma	HeLa_SMN_KD_1_p	HeLa cell culture	Homo sapiens	
ERR1547211	Adenocarcinoma	HeLa_SMN_KD_2_p	HeLa cell culture	Homo sapiens	
SRR1206242	Wildtype	Motor neurons derived from Hb9 normal mESCs	Motor neurons	Mus musculus	Maeda *et al.*
SRR1206243	Wildtype	Motor neurons derived from Hb9 normal mESCs	Motor neurons	Mus musculus	
SRR1206244	Wildtype	Motor neurons derived from Hb9 normal mESCs	Motor neurons	Mus musculus	
SRR1206245	SMN2+/+;mSmn-/-	Motor neurons derived from A2 SMA mESCs	Motor neurons	Mus musculus	
SRR1206246	SMN2+/+;mSmn-/-	Motor neurons derived from A2 SMA mESCs	Motor neurons	Mus musculus	
SRR1206247	SMN2+/+;mSmn-/-	Motor neurons derived from A2 SMA mESCs	Motor neurons	Mus musculus	
SRR1016945	WT	Motor neurons PND1	Motor neurons	Mus musculus	Zhang *et al.*
SRR1016946	WT	Motor neurons PND2	Motor neurons	Mus musculus	
SRR1016947	SMN2+/+;SMN? 7+/+;Smn-/-	Motor neurons PND3	Motor neurons	Mus musculus	
SRR1016948	SMN2+/+;SMN? 7+/+;Smn-/-	Motor neurons PND4	Motor neurons	Mus musculus	
SRR7298263	Control	SC_wt_5	Motor neurons	Mus musculus	Nichterwitz *et al.*
SRR7298264	Control	SC_wt_5	Motor neurons	Mus musculus	
SRR7298265	Control	SC_wt_5	Motor neurons	Mus musculus	
SRR7298266	Control	SC_wt_5	Motor neurons	Mus musculus	
SRR7298267	Control	SC_wt_5	Motor neurons	Mus musculus	
SRR7298268	Control	SC_wt_5	Motor neurons	Mus musculus	
SRR7298269	Control	SC_wt_5	Motor neurons	Mus musculus	
SRR7298270	Control	SC_wt_5	Motor neurons	Mus musculus	
SRR7298271	Control	SC_wt_5	Motor neurons	Mus musculus	
SRR7298272	Control	SC_wt_5	Motor neurons	Mus musculus	
SRR7298273	SMA	SC_ko_5	Motor neurons	Mus musculus	
SRR7298274	SMA	SC_ko_5	Motor neurons	Mus musculus	
SRR7298275	SMA	SC_ko_5	Motor neurons	Mus musculus	
SRR7298276	SMA	SC_ko_5	Motor neurons	Mus musculus	
SRR7298277	SMA	SC_ko_5	Motor neurons	Mus musculus	
SRR7298278	SMA	SC_ko_5	Motor neurons	Mus musculus	
SRR7298279	SMA	SC_ko_5	Motor neurons	Mus musculus	
SRR7298280	SMA	SC_ko_5	Motor neurons	Mus musculus	
SRR7298281	SMA	SC_ko_5	Motor neurons	Mus musculus	
SRR7298282	SMA	SC_ko_5	Motor neurons	Mus musculus	

### Cloning and *in vitro* transcription of mRNA and mRNA-3′UTRs

Candidate mRNA and mRNA-3′UTRs were selected as described within the results section. RNA sequences were imported from Ensembl, and all Sm-sites (canonical and noncanonical) were identified using Supplementary File 3 using a word processor. A duplicate sequence was generated in which each Sm-site (canonical and noncanonical) was mutated to 5′-ACCCCCG-3′ or 5′-ACUCUCG-3′ to provide the ΔSm sequence (Supplementary File 3). Sequences were then loaded into ApE (A plasmid Editor v2.0.61) to identify restriction enzymes that could be added to linearize plasmids for *in vitro* transcription. T7 promoter sequence was added to the 5′-end of the sequence, and PmlI or AflIII restriction sites were added to the 3′-end of the sequence. Invitrogen GeneART Synthesis services were used to generate plasmids (maps available in Supplementary File 4 and reagents can be made available upon request). Upon delivery of plasmids, sequences were confirmed by Sanger Sequencing. Plasmids were linearized using restriction digestion, followed by phenol:chloroform extraction and ethanol precipitation isolation methods. RNAs were *in vitro* transcribed using the HiScribe T7 High Yield Kit (New England Biolabs SE20405), substituting 25% of the UTP concentration with Bio-16-UTP (Invitrogen AM8452), following manufacturer’s instructions. Transcribed, labeled RNAs were purified using Urea-PAGE and correct band sizes were isolated using ultraviolet light shadow casting against a thin layer chromatography plate. RNAs were eluted from polyacrylamide slices in nuclease-free water overnight at 4°C on a nutating mixer. RNAs were quantified using Qubit RNA Broad Range Assay (Invitrogen Q10211).

### Western blots

Whole cell lysates were prepared from NSC-34 and NSC-34 Δ10 cell lines in 6% sodium dodecyl sulfate (SDS)-lysis buffer—62.5 mM Tris, pH 6.8, 6% SDS, 0.5 mM EDTA [84]. Samples were sonicated for 30 s, boiled 5 min and centrifuged at 10 000 × *g* to remove debris. protein lysates were quantified with BCA assay (Thermo Fisher, Pierce BCA cat# 23250) following manufacturer’s recommendations. Assay was read on BioTek Synergy HT spectrophotometer using Gen5 software, reading 562-nm wavelength. Bovine serum albumin standards were fitted to polynomial function in Apple Numbers to back calculate protein concentration.

Fifty micrograms of total protein cytoplasmic lysates or entire immunoprecipitation eluants were prepped to load into 12% sodium dodecyl sulfate (SDS)–polyacrylamide gels using SDS Loading Buffer—62.5-mM Tris, pH 6.8, 6% SDS, 0.5-mM ehtylenediaminetetraacetic acid (EDTA), 100-mM dithiothreitol (DTT), 10% glycerol, 0.004% bromophenyl blue. The Bio-Rad Criterion system was used to perform all sodium dodecyl sulfate–polyacrylamide gel electrophoresis and membrane transfers. Samples were electrophoresed through 4% SDS-polyacrylamide stacking section of the gel under 40 mV using Model 200.2.0 power supply (Bio-Rad), after which the electrophoresis was ramped up to 100 mV by 20 mV increments every 10 min, in SDS-Running Buffer (0.025-M Tris, 0.192-M glycine, 0.1% SDS, pH 8.3). Gel electrophoresis was monitored by addition of 10-μl Amersham ECL Rainbow Marker – Full Range (Cytiva RPN800E). Transfer onto Immobilon-P^SQ^ membrane (Millipore ISEQ00010) was performed in Transfer Buffer (25-mM Tris, 192-mM glycine, 10% MeOH) at 24 V, <3 A using Model DX5 power supply (Idea Scientific).

Membranes were separated between the 38 and 52 kDa protein markers, briefly rinsed with water, then blocked in 5% Carnation dry milk PBS + 0.2% Tween-20 for 1 h at room temperature with gentle mixing. Block was then removed, and membranes were incubated at 4°C overnight with 1% Carnation dry milk PBS + 0.2% Tween-20 supplemented with mouse anti-pan-SMN/Smn [1:1000 (v/v), BD Transduction Laboratories 610647] or mouse anti-Prp19 [1:1000 (v/v), Santa Cruz sc514338]. Blots were then washed five times briefly and three times for 10 min at 4°C using PBS + 0.4% Tween-20. Primary antibodies were detected by incubating with 1:15 000 goat anti-mouse IgG light chain-specific antibody conjugated to horseradish peroxidase (Jackson Immuno 115-035-174) in 1% Carnation dry milk PBS + 0.2% Tween-20 for 1 h at room temperature. Blots were then washed five times briefly and three times for 10 min at 4°C using PBS + 0.4% Tween-20. Blots were treated with SuperSignal West Pico PLUS Chemiluminescence Substrate (Thermo Scientific 34580) and were imaged using Azure Biosystems Sapphire Molecular Imager using Azure Capture Software and bands were quantified in AzureSpot 2.0 using rolling-ball method of background subtraction. Results are recorded as volume under peak for the ∼36 kDa Smn band and ∼55 kDa Prp19 band.

#### Reagents

Protein concentrations were determined by BCA assay (Pierce 23250). Sm-ring assembly reactions used Mouse mab Smith Antigen Ab (Y12) (Novus Biologicals NB600-546) or SmB/B’/N (12F5) (Santa Cruz sc-130670) antibodies bound to Protein-A Dynabeads (Invitrogen 10001D) for Sm-protein immunoprecipitation, and NeutrAvidin-HRP (Invitrogen A2664) for biotin detection. Other Y12 antibodies tested include SNRPB Monoclonal Antibody (Y12) (Invitrogen MA5-13449) and Anti-Smith Antigen Antibody, clone Y12 (Millipore Sigma MABF2793). Antibodies used for western blot detection include anti-pan-SMN/Smn (BD Transduction Laboratories 610647), anti-Prp19 (Santa Cruz sc514338), and goat anti-mouse IgG light chain-specific antibody conjugated to horseradish peroxidase (Jackson Immuno 115-035-174). HRP signal was detected using SuperSignal Femto ELISA substrate (Pierce 37075) or SuperSignal West Pico PLUS Chemiluminescence Substrate (Thermo Scientific 34580). anti-Sm-RIP-Seq experiments were performed using anti-Sm(Y12) antibody (Novus Biologicals NB600-546). PolyA-RNA was isolated using Dynabeads mRNA Purification Kit (Invitrogen 61006). Sequencing libraries were prepared using Takara SMARTer Stranded Total RNA-Seq Kit v3-Pico Input Mammalian (Takara 634451). Plasmids used for *in vitro* transcription (Supplementary File 4) were restricted digested with PmlI (New England Biolabs R0532L), AfeI (New England Biolabs R0652L), or AflIII (New England Biolabs R0541L). Long RNAs were *in vitro* transcribed using the HiScribe T7 High Yield Kit (New England Biolabs SE20405). U4 snRNA was *in vitro* transcribed using MEGAshortscript T7 Transcription Kit (Invitrogen AM1354). Biotin labeling was performed by supplementing *in vitro* transcription reactions with with Bio-16-UTP (Invitrogen AM8452). RNA was quantified using Qubit RNA Broad Range Assay (Invitrogen Q10211).

#### Biological resources

Cell lines used include neural stem cell-like NSC-34, NSC-34 Δ10, and S3-iNPC [66, 83, 84]. Plasmids for *in vitro* transcription and labeling of U4 and U4ΔSm were supplied by the lab of Dr Dan Battle, formerly at The Ohio State University. Sequences for plasmids used for *in vitro* transcription and labeling of mouse and human candidate mRNA or mRNA 3′UTRs are provided in Supplementary Files 3 and 4. All biological reagents can be made available upon request.

#### Statistical analyses

Graphing was performed using ggplot2 in R (version 4.4.1) or GraphPad Prism [version 10.3.0 (461)]. Statistics were performed in R (version 4.4.1). Wald-test comparisons were performed by DESeq2 (version 1.44.0). Pearson correlations were performed for continuous distribution comparisons. Kendell correlations were performed for rank-order comparisons. Wilcoxon tests were performed for all cumulative distribution functions, using the assumptions that the curves were either ‘greater’ or ‘less’ than the curve being compared to. Specific tests, their n, and data being compared are given in each specific plot and figure legend. If required, Wilcoxon rank sum tests were corrected for multiple testing using the Benjamini–Hochberg false discovery rate method. R session info is provided in Github (https://github.com/ajblatnik/sm_ring_assembly_mrna.git) and Zenodo (DOI:10.5281/zenodo.15476098).

#### Novel programs, software, algorithms

Informatics pipelines for identifying Sm-sites, 5′ splice site and branch-point mimics, mapping to intronic regions, for processing sequencing data, and generating figures are provided in Github (https://github.com/ajblatnik/sm_ring_assembly_mrna.git) and Zenodo (DOI:10.5281/zenodo.15476098).

#### Data base referencing

Human and mouse NCBI RefSeq [89] reference genomes and transcript sequences were downloaded from https://www.ncbi.nlm.nih.gov/datasets/. Human and mouse Gencode [90] reference genomes and transcript sequences were downloaded from https://www.gencodegenes.org/. Genomic features, including transcript lengths, biotype, etc. were pulled using Ensembl BiomaRt [91] package installed in R (version 4.4.1). The RNA Binding Protein Data Base (RBPDB) (http://rbpdb.ccbr.utoronto.ca/) [92] was used to search for known proteins that bind sequences similar to the Sm-site. BioRender [Blatnik, A. (2025); https://BioRender.com/ko8o1mi] was used to generate some images in figures, all figures were assembled in Adobe Illustrator 2024 using an Adobe Creative Cloud license. Tables used to generate figures are available in Supplementary Files 1, 2, and 5. All scripts and tables are available in Github (https://github.com/ajblatnik/sm_ring_assembly_mrna.git) and Zenodo (DOI:10.5281/zenodo.15476098).

## Results

### Sm-sites are commonly found in the 3′UTRs of long mRNAs

**Figure 1.**
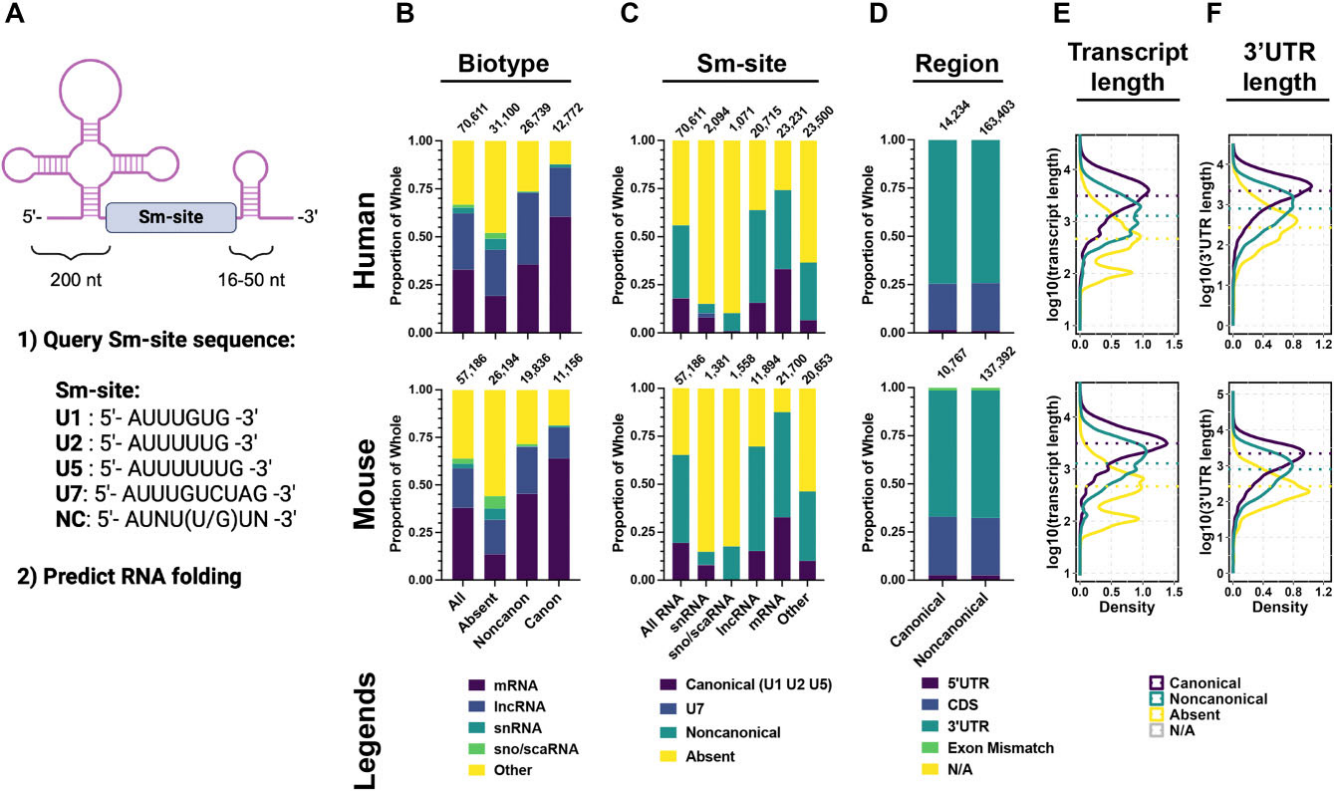
Sm-sites are commonly found in 3′UTRs of long mRNAs. (**A**) Generalized schematic of an snRNA, and the features queried by the search algorithm. First, the NCBI Refseq and Gencode transcriptomes were queried for presence of an Sm-site sequence—defined as U1, U2, U5, U7, or Noncanonical. Second, the 200 nt to the 5′ side of Sm site and 16–50 nt to its 3′ side were queried for thermodynamic favorability to fold using RNAfold. Created in BioRender [Blatnik, A. (2025); https://BioRender.com/ko8o1mi]. (**Top**) For human genes, (**bottom**) for mouse genes. For all, bar graphs, top numbers are the number of genes represented within the bar. Data presented in panels (**B**)–(**F**) correspond to a single, unique transcript ID of a singleunique gene. Genes producing multiple transcripts are pared to only correspond to a single MANE or Ensembl Transcript is Canonical containing transcript. (**B**) Proportional bar graph of RNA biotypes. **All** is a breakdown of the different RNA biotypes within the annotated genome. **Absent** are RNAs not predicted to contain an Sm-site. **Noncanonical** are those RNAs only predicted to contain noncanonical Sm-site sequences. **Canonical** are those RNAs predicted to have at least one U1, U2, or U5 Sm-site sequence, but may have additional canonical or noncanonical sequences. (**C**) Proportional bar graph giving a breakdown of types of Sm-sites predicted in each of the following biotypes: All, small nucleolar (sno) and Cajal-body specific (sca) RNAs (sno/scaRNA), long noncoding RNA (lncRNA), mRNA, and Other. (**D**) Proportional bar graph depicting the region of an mRNA where Sm-sites are predicted. (**E**) Density plot of total transcript lengths categorized by type of Sm-site predicted. (**F**) Density plot of mRNA 3′UTR lengths categorized by the type of Sm-site predicted. See Supplementary Fig. S2 for this analysis broken down for each individually identified U1, U2, U5, U7, and noncanonical Sm-site.

To identify all RNAs in the human and mouse transcriptomes that have features of an Sm-site, we developed a custom algorithm to identify Sm-sites in the corresponding RefSeq and Gencode databases. First, the algorithm identifies transcripts that contain canonical or noncanonical Sm-site sequences. As the canonical Sm-protein ring is classically assembled on U1, U2, and U5-type Sm-site sequences, we defined canonical Sm-site sequences as those pertaining to U1, U2, and U5 snRNAs. Sm-sites from U4, U12, and U4atac snRNAs are classified within the U2-type, and the U11 snRNA site is classified in the U5-type (see Supplementary Fig. S1 for a breakdown of the individual types of Sm-sites within snRNAs). The noncanonical Sm-site sequence was defined as—5′-AUNU(U/G)UN-3′—allowing for base substitutions that do not disrupt Sm-ring assembly [23]. Transcript sequences containing the Sm-site sequence were further filtered to require an RNA secondary structure to start within the 20 nucleotides 5′ of the Sm-site sequence and 10 nucleotides 3′ of the sequence. A schematic describing the sequences and information supplied to the algorithm is provided in Fig. 1A. The identified Sm-sites were cross-referenced between both NCBI RefSeq and Gencode analyses. The output for each type of Sm-site detected within the human and mouse transcriptomes is provided in Supplementary File 1. These data are summarized, providing counts of each Sm-site type on a per-gene basis in Supplementary File 2 (see the ‘Materials and methods’ section for a more detailed description of these tables and their construction). Importantly, the identification of several known Sm-site containing snRNAs and the exclusion of the U6 snRNAs from the predictions demonstrates the validity of our approach for unbiased identification of Sm-site containing RNAs (Supplementary Fig. S1). The inability of our algorithm to identify an Sm-site in human or mouse U4atac suggest its limitations (Supplementary Fig. S1 and Supplementary Files 1 and 2).

Many classes of RNAs were identified to contain Sm-sites, including snRNA, sno/scaRNA, lncRNAs, and mRNAs (Fig. 1B). Surprisingly, although mRNAs comprise about a third of all annotated transcripts (33% in human, 38% in mouse), as a group, they make up an outsized proportion of predicted canonical (60% in human, 64% in mouse) and noncanonical (36% in human, 45% in mouse) Sm-site-containing RNAs (Fig. 1B). Similarly, only about a fifth of all genes are predicted to contain a canonical Sm-site (18% in humans and 19% in mouse; Fig. 1C), but a third of all mRNAs are predicted to contain canonical Sm-sites (33% of human and 33% of mouse mRNAs) suggesting that Sm-sites may be preferentially enriched in mRNAs. The second most-represented group is lncRNAs, making up 26% of human and 16% of mouse canonical Sm-sites (Fig. 1B). Noncanonical Sm-sites are predicted to occur in over a third of human (38%) and nearly half of mouse (46%) annotated genes. Noncanonical Sm-sites appear most frequently in longer RNAs—lncRNA and mRNA—in similar proportions (∼45% in human and 54% in mouse) (Fig. 1C). Only 15% of snRNAs are predicted to contain an Sm-site. This is not surprising given nearly 71% of annotated human snRNAs are U6/U6atac, which does not have an Sm-site and does not receive an Sm-ring [93] (Supplementary Fig. S1 and Supplementary Files 1 and 2). Of the remaining human snRNAs, only 46% are predicted to contain an Sm-site, suggesting many annotated snRNAs may not be functional, may not be Sm-class, or their Sm-site sequence remains to be defined (Supplementary Fig. S1 and Supplementary Files 1 and 2). In all biotypes except snRNAs, U2-type Sm-sites are the most abundant, particularly in lncRNA and mRNAs (Supplementary Fig. S2A and B). U7-type sites are primarily identified in U7 snRNAs and only identified in a handful of other transcripts, supporting our argument to exclude these from our canonical Sm-site definition. These data suggest that many RNAs contain features of snRNAs, and that many of these RNAs are mRNAs.

To assess whether Sm-site prediction is a function of transcript length, we stratified RNAs by the presence (≥1 canonical or noncanonical) or absence of an Sm-site, and plotted them by their transcript length (Fig. 1E). Interestingly, canonical Sm-sites are found in longer transcripts (median transcript length, human = 3092 nt; mouse = 2936 nt) than those that have noncanonical Sm-sites (human = 1281 nt; mouse = 1531 nt) or that do not have Sm-sites (human = 466 nt; mouse = 407 nt) (Fig. 1E). The frequency of canonical Sm-sites is moderately correlated with transcript length (human: Kendell $\tau$ = 0.35, *P* < 2.2 × 10^−16^; mouse: Kendell $\tau$ = 0.35, *P* < 2.2 × 10^−16^) (Supplementary Fig. S3A). The frequency of noncanonical Sm-sites is more strongly correlated with transcript length (human: Kendell $\tau$ = 0.54, *P* < 2.2 × 10^−16^; mouse: Kendell $\tau$ = 0.61, *P* < 2.2 × 10^−16^) (Supplementary Fig. S3B). The finding that the most stringent predicted Sm-sites are more commonly found in longer transcripts than the more promiscuous noncanonical sites suggests that canonical Sm-sites are a feature of these long transcripts and not simply a function of RNA length.

mRNAs are known to contain regulatory elements within their 5′ and 3′ UTRs [94, 95]. To test if Sm-sites are more prevalent in one or more of these regions, Sm-sites identified in mRNAs were binned by their location: 5′UTR, Coding Sequence (CDS), and 3′UTR (Fig. 1D). Additionally, Sm-sites identified in exonic sequences (Exon Mismatch) or other sequences (N/A) that do not clearly match the reference genome were also included (Fig. 1D). We observe that nearly three-fourths of Sm-sites, irrespective of the type of Sm-site, are found in the 3′UTRs of mRNAs (Fig. 1D and Supplementary Fig. S2C). An estimate of the density of Sm-sites showed that canonical Sm-sites are three times more likely to be detected in a 3′UTR than in any other mRNA region (in units of sites/million bases, human: 5′UTR 60.2 CDS 87.7, 3′UTR 291.4; mouse 5′UTR 70.3, CDS 90.7, 3′UTR 260.0). Not only are canonical Sm-sites more likely to be found in mRNA 3′UTRs, but they are also more commonly detected in long 3′UTRs (human: n = 7717 mRNAs, median 2181 nt; mouse: n = 7121 mRNAs, median 1478 nt) (Fig. 1F) whereas noncanonical Sm-sites are found in shorter 3′UTRs (human: n = 9529 mRNAs, median 794 nt; mouse: 11 878 mRNAs, median 645 nt). These observations further strengthen the argument that canonical Sm-sites are preferentially found in mRNA 3′UTRs. Lastly, like overall transcript lengths, 3′UTR length is moderately correlated with the number of canonical sites (human: Kendell $\tau$ = 0.44, *P* < 2.2 × 10^−16^; mouse: Kendell $\tau$ = 0.36, *P* < 2.2 × 10^−16^) (Supplementary Fig. S3C) whereas the correlation between 3′UTR length and noncanonical Sm-site frequency is stronger (human: Kendell $\tau$ = 0.62, *P* < 2.2 × 10^−16^; mouse: Kendell $\tau$ = 0.60, *P* < 2.2 × 10^−16^) (Supplementary Fig. S3D). Taken with the aforementioned results, these data suggest that Sm-sites are more commonly found in the 3′UTRs of longer mRNAs over other RNAs.

### Biochemical identification of RNAs that associate with Sm-proteins in an ATP-dependent manner

The prevalence of Sm-sites in mRNAs and other non-snRNAs suggest that Sm-protein rings may assemble on RNAs beyond snRNAs. Although Lu *et al.* [11] previously reported Sm-protein association with mRNAs, it is not known whether the interaction is dependent on Sm-sites. To identify RNAs that associate with Sm-proteins at equilibrium in human and mouse cell cytoplasmic extracts, we performed anti-Sm (Y12) RNA immunoprecipitation followed by high-throughput sequencing (RIP-Seq) from cytoplasmic extracts of human S3-iNPC and immortalized mouse neuronal stem cells (NSC-34). Previous work from multiple laboratories has shown that Sm-protein rings can be assembled onto the Sm-site of synthetic, labeled U snRNAs by the SMN complex in an ATP-dependent manner in cytoplasmic cell extracts [21, 23, 28, 49, 50]. Such newly assembled U snRNPs can be enriched and quantified by anti-Sm immunoprecipitation in a stringent manner that selects for RNAs that assemble a high-salt and heparin-resistant Sm-ring [21, 23, 28, 50] (Supplementary Fig. S4A). We also sought to determine if Sm-protein rings can be assembled on RNAs other than snRNAs. To this end, a library of polyA-RNA was supplemented into cytoplasmic cell extracts to be used as a substrate (instead of labeled U snRNA) in modified Sm-ring assembly reactions, which were performed in the presence or absence of ATP (ATP+ or ATP−, respectively) (Supplementary Fig. S4B). The cytoplasmic cell extracts and polyA-RNA were prepared from human iNPCs and mouse NSC-34 cell lines, and the assembly reactions were performed in a cross-species manner, i.e. human polyA-RNA incubated with mouse extract and vice versa. This allowed us to identify newly assembled Sm-RNPs from the Sm-RNPs already present in the cytoplasmic extract using the input polyA-RNA libraries from the two cell types as a baseline for the enrichment analysis (Supplementary Fig. S4B). All Sm-RNPs were then identified via anti-Sm RIP-Seq.

To ensure robust, reproducible and equivalent Sm-ring assembly across experimental conditions, several key procedural controls were performed. First, snRNP assembly capacity was normalized between cytoplasmic extracts used for the RIP-seq experiments by empirically determining the amounts of extracts that yield equivalent Sm-ring assembly on a biotinylated *in vitro* transcribed human U4 snRNA (Supplementary Fig. S5A). Second, multiple anti-Sm (Y12) antibodies were tested to determine the source and amount required for saturation of capture (Supplementary Fig. S5B). Third, polyA-RNA after oligo-dT enrichment was confirmed to be depleted of ribosomal RNAs (Supplementary Fig. S5C). Fourth, reactions were scaled up to contain 400-μg total protein in the cytoplasmic extracts and 4 μg of polyA-RNA to provide sufficient yields for sequencing library preparation. Sequencing libraries were generated and sequenced for three biological replicates for each condition presented in Supplementary Fig. S4B. Finally, to further verify specific Sm–RNA associations, modified Sm-ring assembly reactions were repeated under more stringent washing conditions to determine the stability of newly assembled Sm-RNPs and the Sm-RIP conditions at equilibrium were repeated using an SmB/B’/N-specific antibody to ensure captured RNAs were Sm-protein specific.

Alignment of sequencing reads to both the human GRCh38 and mouse GRCm39 genome assemblies using STAR [85] showed that, reads from samples consisting of only human or mouse RNAs align primarily to their corresponding genomes, but poorly to the cross-species genome, as expected (Supplementary Fig. S5D). Furthermore, samples containing RNA from both species show greater read mapping to the genome corresponding to the input polyA-RNA (Supplementary Fig. S5D), suggestive of higher abundance of new Sm–RNA interactions. Less than 10% of genes align to both human and mouse genomes (Supplementary Fig. S5D). These genomically ambiguous RNAs were removed from further analysis. Principal component analysis shows that replicates for each condition are very tightly clustered (Supplementary Fig. S5E). The largest variation between samples is conferred by species and the second largest variation is between the input polyA-RNA condition and Sm-RIP conditions (Supplementary Fig. S5E). All modified Sm-ring assembly reaction conditions cluster together, most closely resembling the Sm-RIP-Seq samples isolated from extracts of the same species the samples were aligned to (Supplementary Fig. S5E). Thus, our libraries are reproducible and specific, and the data indicates robust signal for polyA-RNAs receiving a newly assembled Sm-ring. We will first discuss what RNAs are enriched with Sm-proteins from un-supplemented cytoplasmic extracts and the degree to which the prediction of Sm-sites informs this enrichment. Then we will discuss the degree to which we detect newly assembled Sm-RNPs on exogenously supplemented polyA-RNAs.

### Many RNAs including mRNAs with predicted Sm-sites associate with Sm-proteins

**Figure 2.**
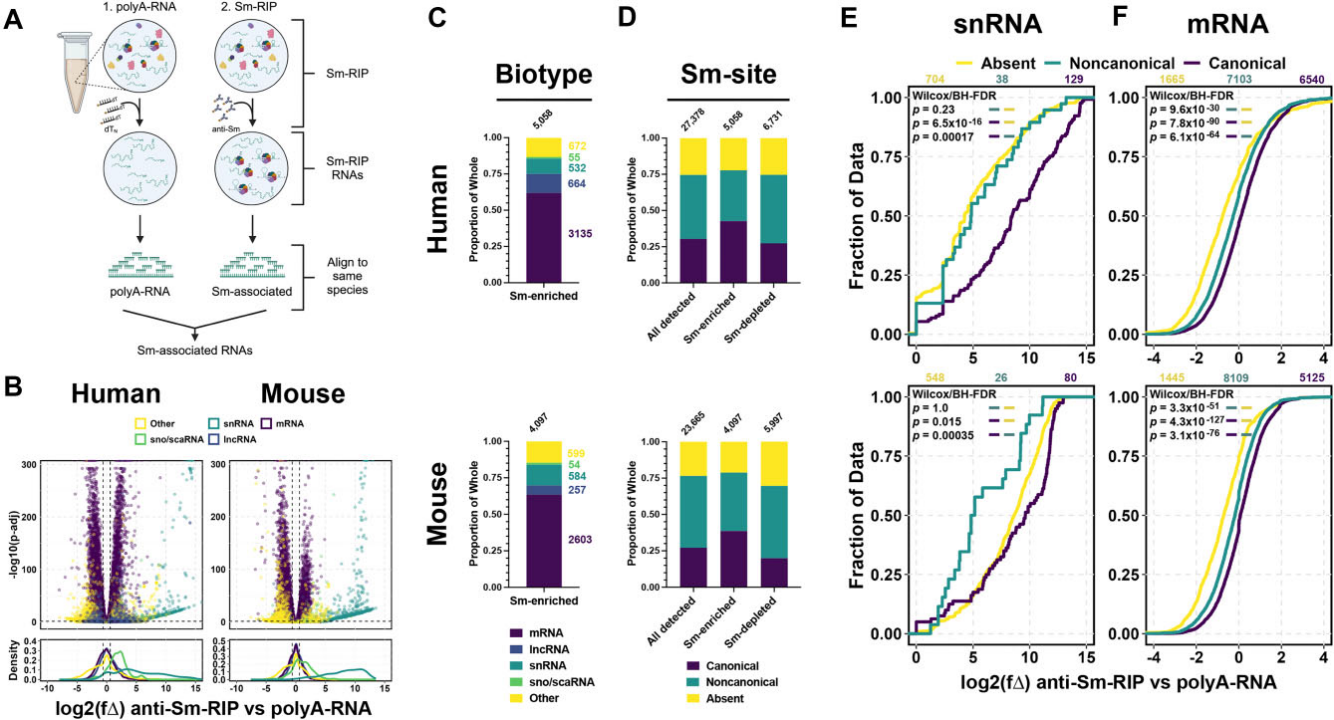
Sm-site containing RNAs are specifically enriched with Sm-proteins. (**A**) Schematic representation of experimental conditions. Created in BioRender [Blatnik, A. (2025) https://BioRender.com/ko8o1mi]. (**B**) Volcano plot and accompanying density plot indicating the different types of RNAs that are enriched in an anti-Sm-RIP experiment when compared to the polyA-RNA enriched transcriptome. *X*-axis for volcano and density plots are shared—log_2_ fold change [log_2_(fΔ)] of anti-Sm-RIP versus polyA-RNA. For all bar graphs, at the top are the number of genes represented within the bar. Values to the side of the bar are the number of genes contained within the portion corresponding to the represented color. (**C–F**) (**Top**) human analysis and **(bottom)** mouse analysis. U7 Sm-site-containing RNAs were removed from analysis as this site is both infrequent and receives a specialized Sm-ring differentiating it from U1, U2, and U5 type Sm-sites. (**C**) Proportional bar graph of RNA biotypes enriched [log_2_(fΔ) > 0.6, *padj*<.05] in anti-Sm-RIP versus the polyA-RNA transcriptome. (**D**) Proportional bar graph giving a breakdown of the Sm-sites. **All** indicates the total different transcripts mapped in the sequenced library, **Sm-enriched** are those transcripts enriched [log_2_(fΔ) > 0.6 *padj* <.05] in the anti-Sm-RIP. **Sm-depleted** are those transcripts that were significantly depleted [log_2_(fΔ) < −0.6, *padj*<.05] in the anti-Sm-RIP. (**E, F**) Cumulative distribution plots for given RNA biotypes, plotting the log_2_(fΔ) by increasing value. Log_2_(fΔ) was calculated between the anti-Sm-RIP versus polyA-RNA conditions. (**E**) snRNA, (**F**) mRNA. Colors designate whether an Sm-site is **Absent** (yellow), **Noncanonical** (green), or **Canonical** (purple). Values above Cumulative Distribution Functions (CDFs) indicate the number of genes plotted for each condition in color. Wilcoxon rank sum tests with continuity correction and Benjamini–Hochberg False discovery rate corrections for multiple testing were used to calculate adjusted *P-*values for the left color being greater than the right color are provided in the upper lefthand corner of the graphs.

Many different species, or biotypes, of RNA have been shown to co-immunoprecipitate with Sm-proteins (Supplementary Fig. S6A) [11]. However, it was not determined if the enriched RNAs contained features of U snRNAs or whether Sm-rings were assembled upon these Sm-associating RNAs [11]. Using our Sm-site prediction method, we determined 28% of the RNAs identified by Lu *et al.* were predicted to contain a canonical Sm-site and 37% were predicted to contain noncanonical Sm-sites (Supplementary Fig. S6B). To expand upon this study and provide the transcriptome context for the RIP, we investigated the type of RNAs enriched in Sm-RIP-Seq samples from NCS-34 and S3-iNPC cytoplasmic cell extracts (without addition of exogenous polyA-RNA and ATP) and compared them to the polyA-RNAs from the same cell line (Fig. 2A).

Many RNA biotypes, other than snRNAs, are represented in the Sm-enriched fraction [log_2_(fΔ) > 0.6, *P*<.05] (Fig. 2B and C). mRNAs comprise 62% of human and 64% of mouse RNAs detected in Sm-RIPs, with the second largest biotypes being lncRNAs and the catch-all “Other” category (Fig. 2C). The proportion of RNAs with a canonical Sm-site is the highest among the Sm-enriched RNAs as compared to RNAs that are depleted (Sm-depleted) and all the total RNAs detected in the analysis (Fig. 2D). On the other hand, the proportion of noncanonical Sm-site containing RNAs is reduced in the anti-Sm-RIP condition, suggesting that noncanonical sites are less deterministic for RNA association with Sm-proteins. Indeed, the proportion of noncanonical sites within the Sm-depleted gene list is similar to all RNAs detected in both human and mouse (Fig. 2D). Furthermore, RNAs predicted to have a canonical Sm-site are more enriched as compared to those predicted to have a noncanonical Sm-site (Supplementary Fig. S7A), further suggesting the noncanonical condition is permissive and likely comprises RNAs that exhibit reduced specificity in Sm-protein association. Surprisingly, only 30% of the RNAs identified by Lu *et al.* are shared in our anti-Sm-RIP dataset (Supplementary Fig. S6C), likely due to the differences in cell type and conditions of the comparisons.

To further test if the prediction of an Sm-site specifies association with Sm-proteins, we examined Sm-RIP enrichment of RNAs with and without Sm-sites from different RNA biotypes—mRNA, lncRNA, snRNA, and sca/snoRNA (Fig. 2E and F, and Supplementary Fig. S7B and C). The presence of an Sm-site in sno/scaRNAs, snRNAs, and mRNAs (and less so in the case of lncRNAs) leads to enrichment with Sm-proteins. For snRNAs and mRNAs, the presence of a canonical Sm-site leads to higher enrichment in comparison to a noncanonical site (Fig. 2E and F). Surprisingly, mRNAs are the largest contributor to the Sm-site containing RNAs enriched in Fig. 2F (compare gene numbers in Fig. 2E and F, and Supplementary Fig. S7A–C). Although sample sizes for snRNAs and sno/scaRNAs are low, human snRNAs that contain Sm-sites are more enriched with Sm-proteins than those without Sm-sites (Fig. 2E, top). Surprisingly, mouse snRNAs devoid of any detectable Sm-sites are nearly as enriched as canonical Sm-site containing snRNAs (Fig. 2E, bottom), suggesting the Sm-site definition used may not fully represent mouse snRNAs.

Importantly, all types of Sm-sites predict further enrichment in an Sm-RIP with U2 and U7-type Sm-site-containing RNAs exhibiting the largest shift in comparison to RNAs lacking Sm-sites (Supplementary Fig. S8). Furthermore, the frequency of Sm-sites within an RNA is directly correlated to RNA enrichment with Sm-proteins (Supplementary Fig. S9), suggesting specificity of Sm-proteins for the Sm-site. RNAs that contain 3 or more predicted canonical Sm-sites are further enriched in Sm-RIPs than those that have only 2, 1, or no predicted Sm-sites (Supplementary Fig. S9A). Additionally, the increased frequency of noncanonical Sm-sites also enhances enrichment in Sm-RIPs of transcripts that contain at least one canonical Sm-site (Supplementary Fig. S9B) or that do not contain a canonical Sm-site (Supplementary Fig. S9C). Transcript length and 3′UTR length does not correlate with Sm-protein enrichment, suggesting that enrichment is due to the presence of the predicted Sm-site and not simply dictated by length of the RNA (Supplementary Fig. S10A and B, respectively). Together, these data support a conclusion that RNAs predicted to contain Sm-sites are specifically and preferentially associated with Sm-proteins in human and mouse extracts under physiological conditions.

Based on our findings that RNAs containing canonical (and noncanonical) Sm-sites are enriched in anti-Sm-RIP-Seq experiments, we predicted that such RNAs will also show preferential binding to Sm-ring components in the published SmB/B’ CLIP-Seq dataset [96]. Indeed, as compared to RNAs lacking Sm-sites, the RNAs predicted to contain Sm-sites and enriched in our anti-Sm-RIPs are also enriched in the published SmB/B’ CLIP-Seq dataset (Supplementary Fig. S6D, F, and G) [96]. Importantly, among the highest quartile of RNAs enriched in the SmB/B’ CLIP-Seq dataset, 578 transcripts are shared with our Sm-RIP-Seq datasets (Supplementary Fig. S6H). A total of 97% of these shared transcripts are mRNAs and 97% are predicted to contain an Sm-site (canonical + noncanonical) (Supplementary Fig. S6I and J). Thus, we conclude that RNAs containing Sm-sites are preferentially associated with Sm-proteins.

### Sm-site containing polyadenylated RNAs associate with Sm-proteins in an ATP-dependent manner

**Figure 3.**
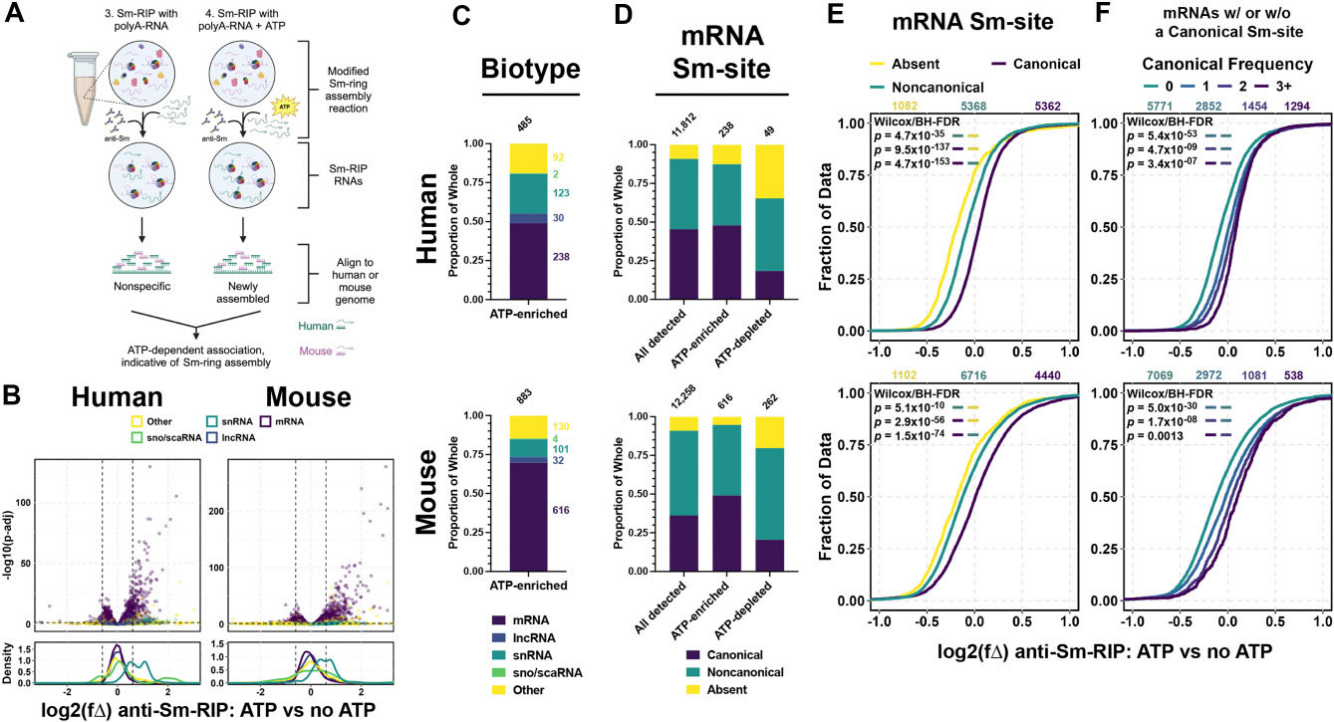
Sm-site containing mRNAs are specifically enriched in anti-Sm-RIPs in an ATP-dependent manner. (**A**) Schematic representation of the experiment. Created in BioRender [Blatnik, A. (2025); https://BioRender.com/ko8o1mi]. (**B**) Volcano plot and accompanying density plot indicating the different types of RNAs that are enriched in a modified Sm-RIP experiment supplemented with polyA-RNA and ATP versus a modified Sm-RIP supplemented polyA-RNA only. X-axis for volcano and density plots are shared—log_2_(fΔ) of anti-Sm-RIP: ATP versus no ATP. (**C**–**E**) (**Top**) for human analysis and (**bottom**) for mouse analysis. For all bar graphs, top numbers are the number of genes represented within the bar and values to the side correspond to the number of genes represented in each color of the graph. U7 Sm-site-containing RNAs were removed from analysis. (**C**) Proportional bar graph of RNA biotypes enriched [log_2_(fΔ) > 0.6, *padj*<.05] in anti-Sm-RIP supplemented with ATP versus anti-Sm-RIP not supplemented with ATP. (**D**) Proportional bar graph giving a breakdown of types of Sm-sites in mRNAs represented in the analysis (**All**), represented in those mRNAs enriched following anti-Sm-RIP [**ATP-enriched**, log_2_(fΔ) > 0.6, *padj*<.05], and those not-enriched [**ATP-depleted**, log_2_(fΔ) < −0.6, *padj*<.05]. (**E, F**) Values above CDFs indicate the number of genes plotted for each condition in color. Wilcoxon rank sum tests with continuity correction and Benjamini–Hochberg false discovery rate corrections for multiple testing were used to calculate adjusted *P-values* for the left color being greater than the right color are provided in the upper lefthand corner of the graphs. (**E**) Cumulative distribution plot of only mRNAs, delineating by Sm-site prediction—**Absent** (yellow), **Noncanonical** (green), **Canonical** (purple). (**F**) Cumulative distribution plot delineating the frequency of canonical Sm-site prediction in an mRNA transcript by log_2_(fΔ) in anti-Sm-RIP supplemented with polyA-RNA and ATP versus anti-Sm-RIPs supplemented only with polyA-RNA. mRNAs predicted to solely contain noncanonical Sm-sites are not plotted in this graph.

Sm-protein ring assembly is an ATP-dependent process in cells and cytoplasmic cell extracts [21, 49, 50]. To identify candidates that most likely accept an Sm-protein ring, we compared RNAs enriched in Sm-RIPs from extracts supplemented with both polyA-RNA and ATP (ATP+) to those without the addition of ATP (ATP−) (Fig. 3A). Notably, in these comparisons, sequencing reads were aligned to the genome reference corresponding to the species from which the polyA-RNA was isolated. For example, when mouse extract was supplemented with human polyA-RNA + ATP and compared to mouse extract supplemented with human polyA-RNA only, reads were aligned to the human genome (Fig. 3A). The capture of several transcripts is significantly different [log_2_(fΔ) > 0.6, *P*<.05 or log_2_(fΔ) > −0.6, *P*<.05] between ATP+ and ATP− conditions, with 485 human and 883 mouse RNAs significantly enriched, and 63 human and 315 mouse RNAs significantly depleted (Fig. 3B–D). The difference in the number of transcripts enriched following the addition of ATP between mouse and human is likely contributed by the 1.8-fold higher amount of mouse extract (438 μg) used in the RIP-Seq experiments as compared to the human extract (250 μg), which was done to accommodate the reduced capacity of mouse extracts to assemble Sm-rings (Supplementary Fig. S5A). Strikingly, the largest fraction of the ATP-induced Sm-enriched RNAs are protein coding (49% for human and 70% for mouse) (Fig. 3C). snRNAs comprise 25% of the human and 11% of the mouse ATP-induced Sm-enriched RNAs, possibly due to their depletion in the polyA-RNA libraries. A vast majority of enriched human (87%) and mouse (99%) mRNAs contain a predicted Sm-site (Fig. 3D) and Sm-site containing mRNAs are globally enriched in extracts supplemented with ATP (Fig. 3E). Akin to the anti-Sm-RIP in un-supplemented extracts (Fig. 2D), noncanonical Sm-sites are proportionally reduced in the ATP and polyA-RNA supplemented condition (Fig. 3D), suggesting the definition of a noncanonical Sm-site used is too permissive or bimodal. Addition of ATP also induces Sm-enrichment of Sm-site containing sno/scaRNA, snRNAs, and lncRNAs (Supplementary Fig. S11B–D). Further, mRNAs with canonical Sm-sites are significantly more enriched upon the addition of ATP than those with noncanonical or absent Sm-sites (Fig. 3D and E). The addition of ATP also enriches U2 and U5-type Sm-site containing mRNAs more than U1, U7, or noncanonical Sm-site containing mRNAs (Supplementary Fig. S12C). The frequency of Sm-sites identified within mRNAs is predictive of further enrichment upon the addition of ATP (Fig. 3F and Supplementary Fig. S13) whereas transcript and 3′UTR length does not correlate with ATP-inducible enrichment (Supplementary Fig. S14A and B, respectively), underscoring that enrichment is due to the presence of *bona fide* Sm-sites. These data show that hallmarks of Sm-ring assembly—Sm-site and ATP-dependence—correlate with enrichment of specific RNAs in *in vitro* cytoplasmic Sm-ring assembly reactions. Together, these data suggest that Sm-protein rings can be more broadly assembled on Sm-site containing RNAs

### Canonical Sm-site prediction drives active Sm-protein:mRNA interaction independent of U snRNP-binding potential

**Figure 4.**
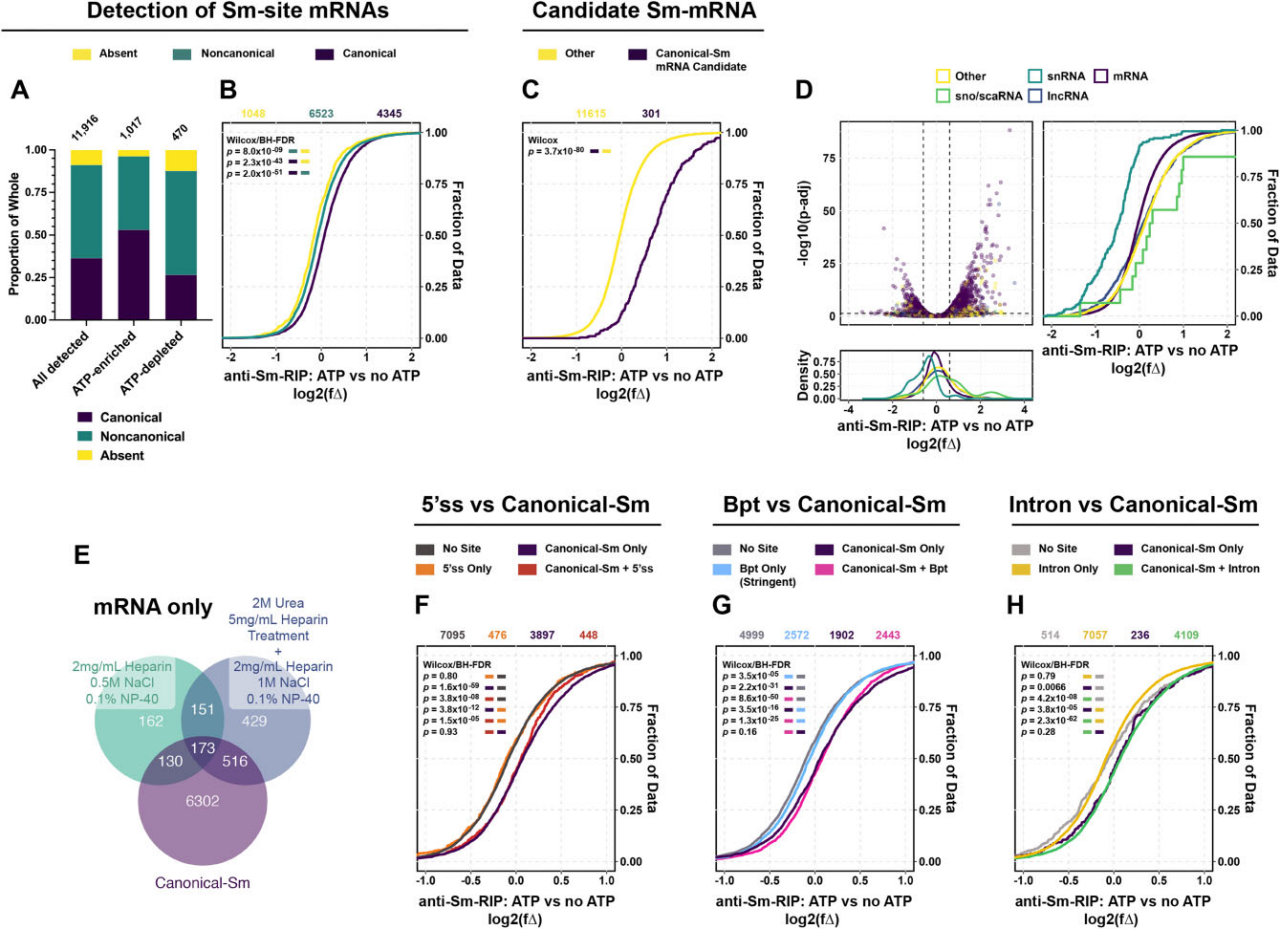
Canonical Sm-site mRNAs are specifically enriched in anti-Sm-RIPs in an ATP-dependent, stringent, and U snRNA-independent manner. For all analyses, U7 Sm-site-containing mRNAs were removed from analysis as this site is both infrequent and receives a specialized Sm-ring differentiating it from U1, U2, and U5 type Sm-sites. (**A, B**) Colors designate whether an Sm-site is **Absent** (yellow), **Noncanonical** (green), or **Canonical** (purple). (**C**) Colors designate whether an mRNA has a canonical Sm-site and was enriched [log_2_(fΔ) > 0.6, *padj*<.05] in anti-Sm-RIP (**Canonical-Sm mRNA Candidate**, purple) or not (**Other**, yellow) (**A**) proportional bar graph of mRNAs enriched [log_2_(fΔ) > 0.6, *padj*<.05] in anti-Sm-RIP: ATP versus no ATP condition, stratified by detected Sm-sites. **All** indicates the total different transcripts mapped in the sequenced library, **Sm-enriched** are those transcripts enriched [log_2_(fΔ) > 0.6 *padj* <.05] in the anti-Sm-RIP. **Sm-depleted** are those transcripts that were significantly depleted [log_2_(fΔ) < −0.6, *padj*<.05] in the anti-Sm-RIP. (**B**) Cumulative distribution plot for mRNAs, plotting the log_2_(fΔ) by increasing value, stratified by whether the RNA contains an **Sm-site**. (**D**) Volcano, density, and cumulative distribution plots for anti-Sm-RIP: ATP versus no ATP experiment treated with 2-M urea and 5-mg/ml heparin and washed with RSB-1000 + 0.1% NP-40. Colors indicate the RNA biotype **mRNA** (purple), **lncRNA** (dark blue), **snRNA** (blue-green), **sno/scaRNA** (green), **Other** (yellow). (**E**) Venn diagram showing overlap of ATP-enriched mRNAs for anti-Sm-RIP-seq performed in Figs 3 and 4, and the list of Canonical Sm-site containing mRNAs. (**F–H**) Cumulative distribution plots for mRNAs, stratified by U snRNA complementary sequence and canonical Sm-site. (**F**) for mimic of 5′ splice sites: **No Site** (dark gray), only a 5′ splice-site mimic (**5′ss Only**, orange), only a Canonical Sm-site (**Canonical-Sm Only**, purple), and the presence of both a canonical Sm-site and 5′ss (**Canonical-Sm + 5′ss**, red). (**G**) for mimic of branch-point sequence: **No Site** (dark gray), only a stringent branch-point mimic (**Bpt Only**, sky blue), only a Canonical Sm-site (**Canonical-Sm Only**, purple), and the presence of both a canonical Sm-site and stringent branch-point mimic (**Canonical-Sm + Bpt**, pink). (**H**) for detection of a read in an intron: **No Site** (gray), detection of an intronic read only (**Intron Only**, gold), only a Canonical Sm-site (**Canonical-Sm Only**, purple), and the presence of both a canonical Sm-site and an intronic read (**Canonical-Sm + Intron**, green). For all CDFs [panels (B), (C), (F), and (H)] log_2_(fΔ) was calculated between the anti-Sm-RIP: ATP versus no ATP conditions performed in stringent washing conditions. Values above CDFs indicate the number of genes plotted for each condition in color. Wilcoxon rank sum tests with continuity correction and Benjamini–Hochberg false discovery rate corrections for multiple testing were used to calculate adjusted *P-*values for the left color being greater than the right color are provided in the upper lefthand corner of the graphs.

Given the striking result that Sm-site containing polyA-RNAs are enriched with Sm-proteins in an ATP-dependent manner, we tested the robustness of these findings by repeating the experimental conditions under a more stringent wash procedure and added several informatic controls to assess the nature of the Sm-protein and RNA association. We incubated mouse polyA-RNA in human cytoplasmic cell extracts in the presence or absence of ATP, after which we treated with 2-M urea and 5-mg/ml heparin to disrupt RNA–RNA interactions [30, 52, 97–99]. Sm-proteins and bound RNA were captured using the Y12 antibody and washed in a 1 M NaCl buffer (RSB-1000 + 0.1% NP-40). Sequences were aligned to the mouse genome, as outlined in Supplementary Fig. S4B and the principal component analysis indicates expected data clustering and low sample variability (Supplementary Fig. S15A). Canonical Sm-site containing mRNAs continue to be specifically enriched in an ATP-dependent fashion (Fig. 4A and B). Furthermore, those canonical Sm-site containing mRNAs enriched in our previous ATP-dependent experiments (Fig. 3D), are almost entirely enriched as a group (Fig. 4C). In contrast to the Sm-RIPs performed in cytoplasmic extracts at equilibrium (Fig. 2B), snRNAs are depleted in these modified Sm-assembly reactions (Fig. 4D). This suggests that canonical Sm-site containing mRNAs may compete with snRNAs for Sm-proteins. A total of 173 canonical Sm-site containing mRNAs are Sm and ATP-enriched in both mild and stringent washing conditions, though many more transcripts predicted to contain canonical Sm-sites are enriched in the more stringent urea/heparin/1-M NaCl conditions (Fig. 4E)—highlighting the robustness of our previous experiments.

The RIP experiments performed by Lu *et al.* [11] and Briese *et al.* [96] indicate that Sm-association with RNAs other than snRNAs occurred through U snRNP-directed sequence complementarity and not by a direct interaction with Sm-proteins. To determine if Sm-captured mRNAs were associating through this mechanism, we defined transcripts containing sequences that match the U1/U11 5′ splice site (5′ss) recognition sequence (5′-MAGGAURAGK), including the noted bulge conformations (including either 1 deletion or 1 mismatch) [100] and the branch-point (Bpt) recognition sequence (U2/U12 Stringent 5′-YMYUNACW or Consensus YUNAYYYYY) [101–103] (Supplementary File 1 and Supplementary Fig. S16). We do observe that mRNAs containing sequences complementary to U snRNAs are enriched in the mild Sm-RIP conditions at equilibrium (Supplementary Figs S17A–D and S18A and B), but mRNAs containing these features are not enriched in extracts supplemented with ATP and polyA-RNA, regardless of wash stringency (Supplementary Figs S15B–E, S19A–D, and S20A and B). Indeed, mRNAs predicted to contain a canonical Sm-site are as globally Sm-enriched as mRNAs containing both an Sm-site and U snRNP complementary sequence in ATP-supplemented conditions (Fig. 4F and G, and Supplementary Fig. S20A and B), suggesting the Sm-site is driving Sm-protein association.

We additionally determined if mRNA capture in our Sm-RIPs was due to U snRNP canonical function in binding to pre-mRNA via retained introns. To this end, we tested for the presence of intronic sequences in the immunoprecipitated RNAs and for correlation between mRNA enrichment and exon number. Nearly 91%–95% of the mRNAs captured in Sm-RIPs are assigned at least one intronic count (Supplementary Figs S17E and S19E). Indeed, mRNAs with at least one intronic count are more globally enriched than those mRNAs without intronic reads at equilibrium (Supplementary Figs S17E and F and S18C). Further, transcripts with a higher number of exons are more enriched with Sm-proteins than transcripts with fewer exons (Supplementary Fig. S17G). However, as with the aforementioned U snRNP-binding features, retained intronic sequence and high exon mRNAs are not globally enriched in extracts supplemented with ATP and polyA-RNA (Fig. 4H, and Supplementary Figs S19E–G and S20C), again suggesting the Sm-site is conferring Sm-protein association.

These data strongly suggest that ATP-dependent capture of canonical Sm-site containing mRNAs in association with an assembled U snRNP. In conclusion, the robustness of canonical Sm-site mRNA prediction and ATP-dependent capture with an anti-Y12 antibody has identified a group of mRNAs that competes with snRNAs for Sm-proteins, strongly suggesting direct Sm:mRNA interaction that resembles assembly and stability of an Sm-protein ring on snRNAs.

### Sm-site containing mRNAs are also specifically enriched in SmB/B’/N and Y12 RIPs

**Figure 5.**
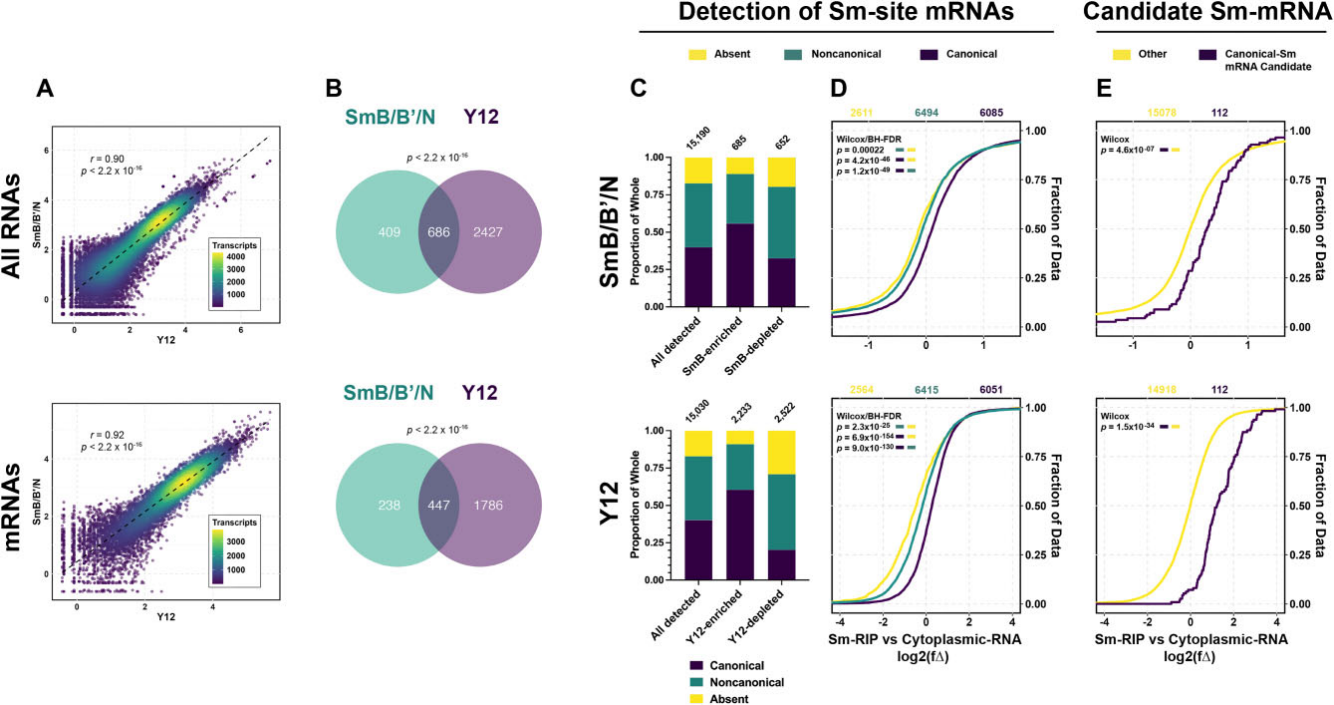
Sm-site containing mRNAs are enriched by both Y12 and SmB/B’/N-specific antibodies. (**A, B**) (**Top**) All RNAs captured by SmB/B’/N and Y12 antibodies, (**bottom**) mRNAs captured by SmB/B’/N and Y12 antibodies. (**A**) Scatter plot of Variance Stabilizing Transformed (VST)-normalized counts for RNAs captured by Y12 and SmB/B’/N-specific antibodies. *r*-values are a Pearson correlation coefficient. (**B**) Venn diagrams of SmB/B’N and Y12 enriched [log_2_(fΔ) > 0.6 *padj* <.05] transcripts. *P-*values are calculated from a Fisher’s exact test. (**C–E**) (**Top**) SmB/B’/N analysis and (**bottom**) Y12 analysis. U7 Sm-site-containing mRNAs were removed from analysis as this site is both infrequent and receives a specialized Sm-ring differentiating it from U1, U2, and U5 type Sm-sites. (**C**) Proportional bar graph of mRNAs enriched [log_2_(fΔ) > 0.6, *padj*<.05] in anti-Sm-RIP versus the cytoplasmic RNA transcriptome, stratified by detected Sm-sites. **All** indicates the total different transcripts mapped in the sequenced library, **Sm-enriched** are those transcripts enriched [log_2_(fΔ) > 0.6 *padj* <.05] in the anti-Sm-RIP. **Sm-depleted** are those transcripts that were significantly depleted [log_2_(fΔ) < −0.6, *padj*<.05] in the anti-Sm-RIP. Colors designate whether an Sm-site is **Absent** (yellow), **Noncanonical** (green), or **Canonical** (purple). (**D, E**) Cumulative distribution plots for mRNAs, plotting the log_2_(fΔ) by increasing value, stratified by Sm-site group. (**D**) for Sm-site: **Absent** (yellow), **Noncanonical** (green), or **Canonical** (purple). (**E**) for canonical Sm-site mRNAs enriched in the ATP+ condition in Fig. 3. Colors designate whether an mRNA is a **Canonical-Sm mRNA Candidate** (purple) or **Other** (yellow). Log_2_(fΔ) was calculated between the anti-Sm-RIP versus cytoplasmic RNA conditions. Values above CDFs indicate the number of genes plotted for each condition in color. Wilcoxon rank sum tests with continuity correction and Benjamini–Hochberg false discovery rate corrections for multiple testing were used to calculate adjusted *P-values* for the left color being greater than the right color are provided in the upper lefthand corner of the graphs.

The Y12 antibody detects Sm-proteins through binding their symmetrically di-methylated arginine-rich tails—a feature not exclusive to Sm-proteins [104–106]. To ensure that Sm-site RNA capture is Sm-protein specific, we compared Y12 antibody capture with an SmB/B’/N-specific capture. We performed SmB/B’/N and Y12 RIP-Seq from human S3-iNPC cytoplasmic cell extracts, treated with 2-M urea and 5-mg/ml heparin to disrupt RNA–RNA interactions, and washed the immunoprecipitates with the stringent 1-M NaCl buffer (RSB-1000 + 0.1% NP-40). Principal component analysis indicates the SmB/B’/N condition is more variable and shares more similarity than Y12 to human cytoplasmic RNA, suggesting the SmB/B’/N capture was weaker (Supplementary Fig. S21A). Consistently, the significance and fold change ranges in the SmB/B’/N capture are nearly a magnitude smaller for SmB/B’/N (Supplementary Fig. S21B). Despite these differences, profiles of RNAs captured with both antibodies are remarkably similar (Supplementary Fig. S21B). Indeed, all RNAs as well as mRNAs enriched with each antibody are largely correlated (Pearson *r* = 0.90 and 0.92, respectively) (Fig. 5A). A total of 686 RNAs are enriched in both RIPs, of which 447 are mRNAs (Fig. 5B). Both antibodies show identical capture and enrichment of Sm-site containing mRNAs (Fig. 5C and D), as well as mRNAs containing U snRNP-binding potential (Supplementary Fig. S22). Not only are canonical Sm-site containing mRNAs enriched with both antibodies, but the treatment with 2-M urea, 5-mg/ml heparin, and 1-M NaCl washes shifts the noncanonical Sm-site containing group closer to those mRNAs not predicted to contain an Sm-site, further suggesting the noncanonical group is too permissive (Fig. 5D). However, canonical Sm-site containing mRNAs that were enriched in an ATP-dependent manner (Fig. 3D) were also enriched with both SmB and Y12 capture (Fig. 5E). These data show the Sm-association with canonical Sm-site mRNAs is not restricted to the Y12 antibody, but a feature also shared by an antibody specific for SmB/B’/N—a component of the final step in assembling the Sm-ring [5, 12, 15, 26]. Additionally, these data highlight the robustness of our previous experiments, showing that canonical Sm-site mRNAs enriched with Sm-proteins in an ATP-dependent manner under milder washing conditions were also enriched with Sm-proteins using two different antibodies under more stringent washing conditions. These data strongly argue that Sm-protein association with these canonical Sm-site containing mRNAs is direct and stable—indicative of an Sm-ring.

### Sm-proteins can be directly assembled on *in vitro*-transcribed Sm-site containing mRNA 3′UTRs in an SMN dependent manner

**Figure 6.**
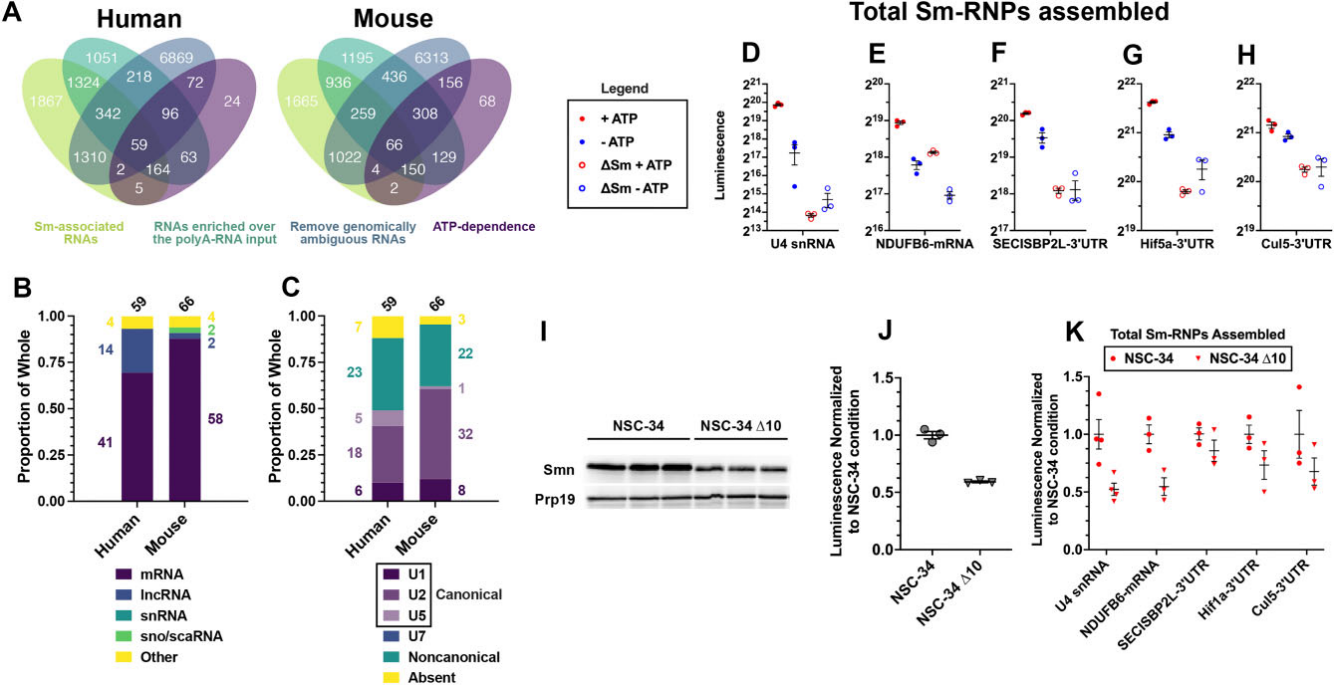
Sm-protein rings are directly assembled on mRNAs in an ATP, Sm-site, and SMN-dependent manner. (**A**) Venn diagrams depicting the comparisons made to select candidate mRNAs to test for Sm-ring assembly directly. Each ellipse is representative of gene products enriched [log_2_(fΔ) > 0.6, *padj* <.05] in the noted comparison. The ∼60 genes in the center are enriched in each comparison, comprising the candidate list. For panels (**B**) and (**C**), values above bars indicate the number of genes contributing to the plots. Numbers in color to the sides of bars indicate the number of genes contributing to the specified group within the bar. (**B**) Proportional bar graph of the RNA biotypes for gene products enriched [log_2_(fΔ) > 0.6, *padj*<.05] within each comparison depicted in panel (A). (**C**) Proportional bar graph giving a breakdown of types of Sm-sites predicted in RNA enriched [log_2_(fΔ) > 0.6, *padj*<.05] in all comparisons, depicted in panel (A). **(D–H)** Luminescence results from detection of *in vitro* transcribed, biotin-labeled human U4 snRNA (**D**), NDUFB6 mRNA (**E**), and mRNA 3′UTRs [SECISBP2L (**F**), Hif1a (**G**), and Cul5 (**H**)] enriched following 15-min treatment with 2-M urea and 5-mg/ml heparin, Y12 immunoprecipitation in 2-mg/ml heparin, RSB-500 + 0.1% NP-40 and washed eight times with RSB-500 + 0.1% NP-40. Four conditions were performed for each RNA: (solid red dot) cytoplasmic cell extract supplemented with wild-type RNA and ATP, (solid blue dot) cytoplasmic cell extract supplemented with wild-type RNA but not with ATP, (open red circle) cytoplasmic cell extract supplemented with ATP and RNA mutated to remove the Sm-site sequence, and (open blue circle) cytoplasmic cell extract supplemented with RNA mutated to remove the Sm-site sequence but not ATP. (**I**) Western blot for mouse Smn and Prp19 protein expression in cytoplasmic lysates prepared from WT Smn (NSC-34) and 50% Smn (NSC-34 Δ10) cell lines [64, 82]. (**J**, **K**) Cytoplasmic extracts prepared from WT Smn (NSC-34, square) and 50% Smn (NSC-34 Δ10, triangle). (**J**) Quantification of blot in panel (**I**), normalized to the WT Smn (NSC-34) condition. (**K**) +ATP luminescence relative to the WT Smn (NSC-34) condition for U4 snRNA and Sm-site candidate mRNAs.

Though Sm-site containing RNAs are associate with Sm-proteins in an ATP-dependent manner, this does not indicate Sm-protein rings specifically assemble at the predicted Sm-sites within these RNAs. Therefore, to identify the best candidate RNAs to directly test Sm-protein ring assembly on Sm-sites of representative polyA-RNAs, we compared different experimental conditions indicated in Supplementary Fig. S4B and applied a series of filtering steps (Fig. 6A). Top candidate RNAs were expected to be enriched upon the addition of ATP, genomically unambiguous, enriched over the polyA-RNA input library, and to associate with Sm-proteins. Enrichment within a particular experimental comparison was defined as a >1.5-fold change with an adjusted *P*-value <.05 [log_2_(fΔ) > 0.6, *P*<.05]. A total of 59 human and 66 mouse candidate RNAs satisfy the full set of conditions (Fig. 6A and Supplementary File 5). Of these candidate RNAs, the majority are mRNAs (69% in human and 88% in mouse) (Fig. 6B). Indeed, 71% of human and 86% of mouse candidate RNAs contain an Sm-site (Fig. 6C). This data further underscores that Sm-site containing polyA-RNAs, particularly mRNAs, are robust candidates to accept an Sm-protein ring following the rules governing U snRNP assembly.

For validating Sm-ring assembly on specific candidates in cytoplasmic extracts, we focused on candidate mRNAs that (i) contain a canonical Sm-site within the 3′UTR at a similar locus of the mRNA in both mouse and human orthologs, (ii) do not have multiple canonical Sm-sites at vastly different locations of the mRNA, and (iii) are devoid of noncanonical Sm-sites. Candidate mRNAs and 3′UTRs were *in vitro* transcribed in the presence of biotin-UTP. As controls, Sm-sites, canonical and/or noncanonical were mutated in which all uridines were changed to cytosines or alternating cytosines (i.e. from 5′-AUUUUUG-3′ to 5′-ACCCCCG-3′ or 5′-ACUCUCG-3′). Both candidate RNAs containing and lacking their respective Sm-sites were then assayed for Sm-protein ring assembly [50], testing for ATP-inducibility, SMN and Sm-site dependence of assembly, mimicking that of a similarly labeled human U4 snRNA.

Some mRNAs and 3′UTRs containing canonical Sm-sites show both an ATP and Sm-site dependence in Sm-protein ring assembly under conditions previously shown to select for RNAs with assembled Sm-rings (500-mM NaCl and 2-mg/ml heparin), as described by Wan *et al.* (Supplementary Fig. S23A–G left). These include the human NDUFB6 mRNA, and the mouse Hif1a and Cul5 3′UTRs (Supplementary Fig. S23B, E, and F left). Despite exhibiting the highest luminescence, the human KIF5B-3′UTR only shows an Sm-site dependence and not an additional ATP-inducible increase in luminescence signal (Supplementary Fig. S23C left). Furthermore, the only noncanonical Sm-site containing mRNA tested, mouse Rem2, showed only a modest ATP and Sm-site dependence (Supplementary Fig. S23G left).

To test Sm-ring assembly under conditions that could disrupt RNA–RNA interactions [30, 52, 97–99], we performed Y12 immunoprecipitations in 500-mM NaCl and 2-mg/ml heparin following treatment with 2-M urea and 5-mg/ml heparin. (Fig. 6D–H and Supplementary Fig. S23A–G right). Under these more stringent conditions, the maximal luminescence signal detected was reduced, but the signal between the ATP with intact Sm-site condition from the other conditions was widened—indicating a more stable and specific Sm-ring assembly (Fig. 6D–H and Supplementary Fig. S23A–G right). Urea/heparin treatment confirmed specific Sm-protein ring assembly on human NDUFB6 mRNA, on mouse Hif1A and Cul5 3′UTRs, and additionally on the SECISBP2L 3′UTR (Fig. 6E–H and Supplementary Fig. S23B and D–F right). Sm-site dependence but a lack of ATP-dependence was likewise confirmed for the human KIF5B 3′UTR (Supplementary Fig. 23C right). The mouse Rem2 mRNA showed no increase in luminescence signal following urea/heparin treatment, indicating that Sm-ring assembly on this candidate mRNA is not specific (Supplementary Fig. S23G right). Furthermore, stable Sm-ring assembly on NDUFB6 mRNA and Hif1a-3′UTR can also be detected via immunoprecipitation using an anti-SmB/B’/N specific antibody (Supplementary Fig. S24).

Sm-ring assembly is performed by the SMN Complex and the capacity to assemble Sm-rings in cytoplasmic extracts has a positive linear correlation with SMN protein abundance [21, 49, 50]. To determine whether SMN is required for Sm-mRNPs, we employed the NSC-34 cell lines we previously characterized to constitutively express 50% of the normal mouse Smn protein expression (Fig. 6I and J) [66, 84]. Using cytoplasmic extracts prepared from these cells, all tested mRNAs, with the exception of SECISBP2L 3′UTR, show an Smn-dependent response—detecting nearly half the luminescence in the 50% Smn cytoplasmic extract in comparison to the wild-type NSC-34 extract (Fig. 6K). These data confirm that Sm-protein rings can be assembled on mRNAs if they contain a canonical Sm-site and that this assembly is dependent on the known Sm-ring assembly machinery—the SMN Complex.

### Sm-site containing mRNAs have a reduced abundance in SMA animal and cell models

**Figure 7.**
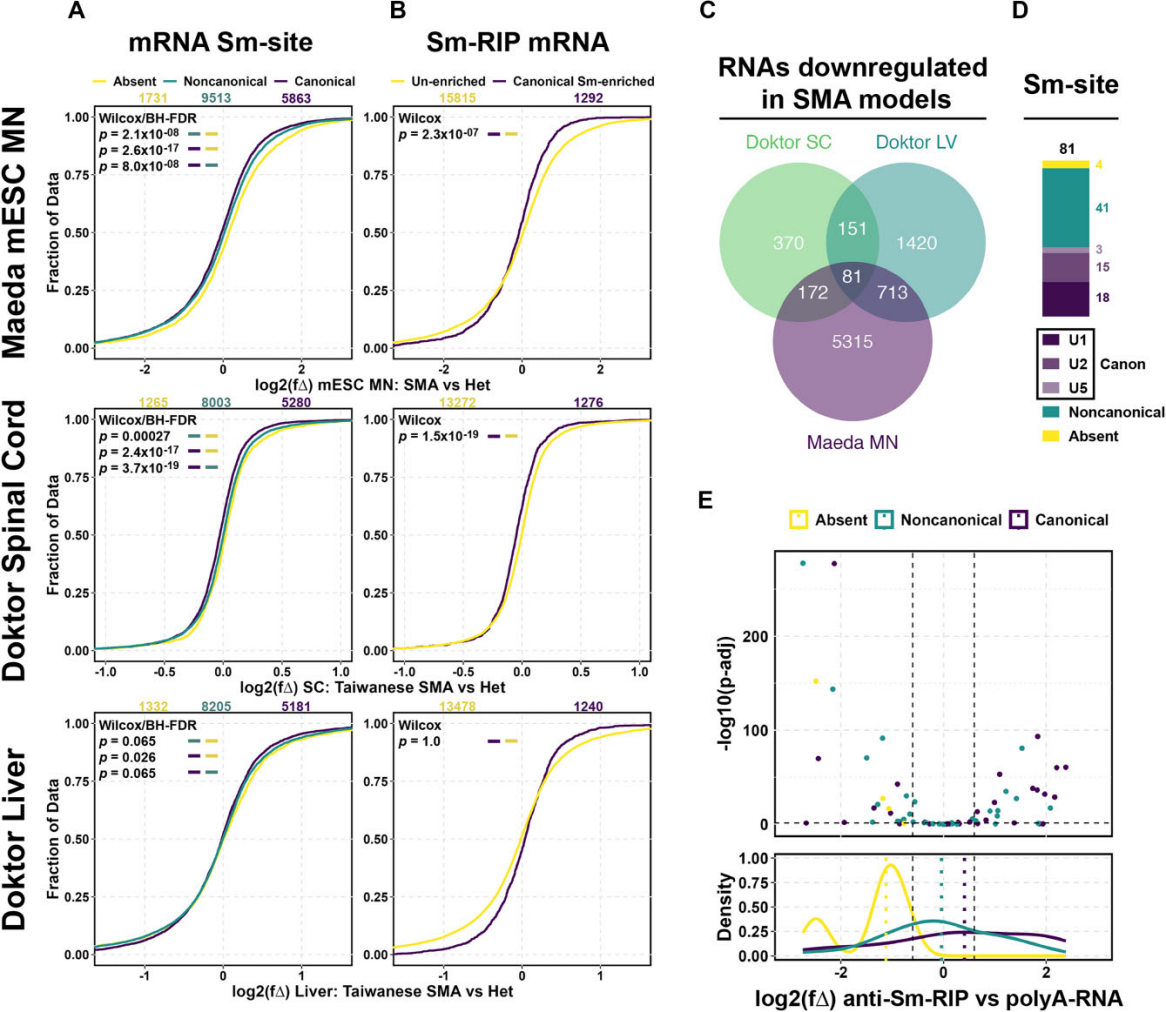
mRNAs predicted to have canonical Sm-sites have a reduced abundance in low SMN conditions. (**A, B**) Cumulative distribution function plots of mRNA fold changes in low SMN conditions compared to normal SMN conditions: (**top**) comparison of mESC differentiated motor neurons derived from SMA (*Smn^+/−^*;*SMN2^+/+^*) and normal (*Smn^+/+^*;*SMN2^+/+^*) as published by Maeda *et al.* (PLoS One, 2014) [96], (**middle**) comparison of spinal cord lysates collected from post-natal day 5 Taiwanese SMA (*Smn^−/−^*; *SMN2^+/+^*) and Taiwanese Het (*Smn^+/−^*;*SMN2^+/+^*) mice as published by Doktor *et al.*(NAR, 2017) [97], and (**bottom**) comparison of liver lysates collected from post-natal day 5 Taiwanese SMA (*Smn^−/−^*;*SMN2^+/+^*) and Taiwanese Het (*Smn^+/−^*;*SMN2^+/+^*) mice as published by Doktor *et al.* (NAR, 2017) [97]. Values above CDFs indicate the number of genes plotted for each condition in color. Wilcoxon rank sum tests with continuity correction and Benjamini–Hochberg false discovery rate corrections for multiple testing were used to calculate adjusted *P-values* for the left color being greater than the right color are provided in the upper lefthand corner of the graphs. (**A**) Plots delineated by Sm-site prediction—**Absent** (yellow), **Noncanonical** (green), **Canonical** (purple). (**B**) Plots delineated by whether those transcripts were enriched [log_2_(fΔ) > 0.6, *padj*<.05] in anti-Sm-RIPs (**Sm-enriched**, purple) or **Un-enriched** (yellow). (**C**) Venn diagram indicating the shared transcripts with reduced abundance [log_2_(fΔ) < 0, *padj*<.05] when SMN is deficient. (**D**) Proportional bar graph giving a breakdown of types of Sm-sites predicted in RNAs shared between each SMN-deficient dataset. Values above bars indicate the number of genes contributing to the plots. Numbers in color to the sides of bars indicate the number of genes contributing to the specified group within the bar. (**E**) Volcano and density plot for 81 shared genes with reduced abundance in SMA-models as detected in the anti-Sm-RIP versus the polyA-RNA comparison.

A reduced capacity to assemble U snRNPs has been shown to result in reduced stability of U snRNAs [107], thus we hypothesized that this may also be the case for other RNAs predicted to contain Sm-sites. We mined total RNA-seq datasets from four studies [108–111] in three mouse models of SMA [112–114] to determine if there was a correlation between the abundance of mRNAs containing Sm-sites and the SMA condition. In this analysis, we included the three RNA-seq datasets where the control and disease samples differentiate along the first component of a principal component analysis (PC1): mouse embryonic stem cell derived motor neurons from severe SMA (*Smn^−/−^;tg89SMN2^+/+^*) mice [109], and the spinal cord and liver datasets generated from post-natal day 5 ‘Taiwanese’ SMA (*Smn^−/−^;SMN2^+/−^*) mice [110] (Supplementary Fig. S25A–C). We find that, canonical Sm-site containing mRNAs are globally reduced in abundance versus mRNAs lacking Sm-sites in severe SMA mouse embryonic stem cells differentiated to motor neuron fates (Fig. 7A, top). Furthermore, this effect is shared, though milder, in spinal cord samples generated from post-natal day 5 ‘Taiwanese’ SMA mice (Fig. 7A, middle). Though significantly reduced in abundance, the shift is less apparent for Sm-site containing mRNAs in the liver (Fig. 7A, bottom), possibly suggesting cell type specificity. Importantly, canonical Sm-site mRNAs enriched in our Sm-RIP experiments mimic the reductions seen for Sm-site containing mRNAs (Fig. 7B). Perhaps surprisingly, only 81 significantly downregulated (log_2_fΔ < 0, *padj*<.05) genes are shared across the three datasets and nearly all of these are mRNAs (Fig. 7C). Of these 81 genes, 95% contain a predicted Sm-site, potentially identifying RNAs particularly susceptible to low levels of SMN protein abundance (Fig. 7D). Lastly, the majority of the canonical Sm-site containing mRNAs that are downregulated are also associated with Sm-proteins (Fig. 7E). Taken together, these data suggest Sm-site containing mRNAs, are less stable when SMN is deficient, mimicking the known physiology for U snRNAs and thereby underscoring the relevance for our findings to physiological states in SMA.

### Sm-ring associated mRNAs are strongly correlated with mRNAs enriched in motor neuron soma and alpha-COP vesicles

With the finding that Sm-site containing mRNAs are less abundant in SMA models, we asked whether these mRNAs are also identified in cellular processes shown to be affected by SMN-deficiency. One hypothesis in the SMA field is that SMN-deficiency affects mRNA trafficking for translation at specific sub-cellular locations, particularly at the axon terminal. To test whether the Sm-site affects this process, we analyzed enrichment of Sm-site containing RNAs in an axonal versus soma sequencing dataset generated from mouse embryonic stem cell derived motor neurons [115]. This dataset determined the axonal transcriptome is distinct from the soma transcriptome, and identifies mRNAs trafficked to be locally translated at axon terminals. In rejection of our initial hypothesis, Sm-site containing mRNAs are massively enriched in motor neuron somas (Supplementary Fig. S26A). Furthermore, Sm-associated mRNAs are also more enriched in motor neuron somas than axons (Supplementary Fig. S26B). These data reject the hypothesis that Sm-sites and Sm-ring assembly are a signal for mRNA axonal trafficking.

Additionally, RNAs have been shown to associate with vesicle complexes of the endoplasmic reticulum and Golgi apparatus, mediated through the alpha subunit of the coatamer protein I (COPI) complex—alpha-COP [74]. Not only does SMN and alpha-COP inhabit a similar sub-cellular locale, but over-expression of alpha-COP was shown to suppress the effects of SMN-deficiency in SMA zebrafish and mouse models [116, 117]. Previously, another group identified alpha-COP associated RNAs using formaldehyde cross-linking [74]. Thus, we asked if these alpha-COP associated RNAs were enriched in our Sm-RIP dataset. Of the 1519 RNAs enriched in alpha-COP RIPs, 1315 of them were present in our anti-Sm-RIP dataset and were more globally associated with Sm-proteins (Supplementary Fig. S26C). A total of 460 of the alpha-COP associated RNAs are significantly enriched with Sm-proteins, and almost all of them are mRNAs predicted to contain canonical or noncanonical Sm-sites (Supplementary Fig. S26D and E). These correlations between Sm-sites, Sm-association, and COPI-mediated trafficking of these mRNAs suggest a possible mechanism for alpha-COP-mediated suppression of SMN-deficiency in SMA.

## Discussion

### Sm-sites are not restricted to snRNAs and are prevalent in mammalian transcriptomes

Our algorithm searches for Sm-sites modeled on established features of the human U snRNAs [23, 28] and identified Sm-sites in all major RNA biotypes, including snRNAs and mRNAs. Multiple observations regarding the detection of Sm-sites in snRNAs indicate the specificity and precision of the algorithm. First, we detect Sm-sites in all snRNAs known to contain these sequences with the exception of U4atac. U4atac is particularly unique in that there are only 8 nucleotides 3′ of the Sm-site which are not predicted to fold into a structure [93]. As our algorithm requires both a 5′ and 3′ stem loop structure, it would not be expected to identify an Sm-site in U4atac. Second, the algorithm rarely detects Sm-sites in U6 snRNAs (2.6% human, 0% mouse), which lack Sm-sites, are not exported from the nucleus, and do not receive an Sm-ring [118–120]. Third, our anti-Sm-RIP-Seq experiments show that human snRNAs detected by the algorithm to contain canonical Sm-sites are enriched over snRNAs predicted to contain a noncanonical Sm-site, or those snRNAs with no prediction for an Sm-site (Fig. 2E, top). Thus, Sm-site containing RNAs identified across the transcriptome by our algorithm are highly likely to be targets of Sm-ring assembly.

We included noncanonical Sm-sites in our study to account for the well-known base-substitutions that still result in Sm-association and ring assembly [23]. We find that while noncanonical Sm-site containing snRNAs show a lower association with Sm-proteins than canonical Sm-site containing snRNAs (Fig. 2C, top), they are further enriched over snRNAs lacking Sm-sites in reactions supplemented with ATP (Supplementary Fig. S11C, top). These observations suggest that noncanonical Sm-sites likely accept Sm-rings. The inclusion of noncanonical sites in our search algorithm allowed us to make a comprehensive list of transcripts that exhibit a potential for Sm-ring assembly. Given the increased number of possible sequences that can satisfy the noncanonical Sm-site definition, it is not surprising that nearly twice as many transcripts contain noncanonical Sm-sites than canonical Sm-sites (Fig. 1B) and noncanonical Sm-site frequency is strongly correlated with transcript and 3′UTR length (Supplementary Fig. S3). Like snRNAs, mRNAs containing noncanonical Sm-sites exhibit a higher affinity for Sm-proteins than mRNAs lacking an Sm-site, when assembled endogenously and after supplementation with ATP (Figs 2F, 3E, and 4B, and Supplementary Figs S8C, S9BC, S12C, and S13). Thus, we propose that the noncanonical Sm-sites group is bimodal—likely comprised of both *bona fide* Sm-sites and sites that do not accept Sm-protein ring assembly. In support of this, the Rem2 mRNA, which is predicted to contain only noncanonical Sm-sites, does not show an ATP-dependent or Sm-site dependent association with Sm-proteins (Supplementary Fig. S23G).

A large fraction of annotated snRNAs are not predicted to contain an Sm-site. These include U6/U6atac snRNA that comprise ∼71% of the human annotated snRNAs. Of the remaining human spliceosomal and U7 snRNAs, 54% are not predicted to contain an Sm-site (Fig. 1C and Supplementary Fig. S1 top). Our data suggests that human snRNAs lacking an Sm-site likely do not receive an Sm-ring (Fig. 2E and Supplementary Fig. S11C), suggesting these variants may be nonfunctional forms of the classical snRNAs. In contrast, mouse snRNAs predicted to lack an Sm-site exhibit similar affinity for Sm-proteins as canonical Sm-site containing snRNAs in our standard anti-Sm-RIPs (Fig. 2E, bottom), but do not exhibit this same affinity in response to supplementing reactions with ATP (Supplementary Fig. S11C, bottom). Provided the annotations for mouse snRNAs are correct, these data suggest that the current definition of a mouse Sm-site may be incomplete. This may not be surprising as mouse snRNAs are very poorly annotated (Supplementary Fig. S1, bottom). Also, our program may be unable to correctly identify all mouse snRNAs that receive an Sm-ring as we defined Sm-sites based on the human sequences. Alternatively, it is also possible, though unlikely, that a large fraction of mouse snRNAs may not require ATP to receive an Sm-ring. Overall, our algorithm specifically identifies Sm-sites in the expected snRNAs and can additionally inform the Sm-ring potential of specific variants.

### Evidence for Sm-ring assembly on Sm-site containing mRNAs

Our findings that Sm-site containing RNAs, in addition to snRNAs, are significantly associated with Sm-proteins (Figs 2D–F and 5A–C, and Supplementary Figs S7 and S8) suggests that Sm-protein association with RNAs is more widespread in mammalian transcriptomes than previously known. Although mRNAs and sno/scaRNAs have been previously shown to associate with Sm-proteins, the association was suggested to occur through base-pair complementarity between the 5′ end of U snRNAs in already assembled snRNPs and the target mRNA [11, 96]. These previous studies could only assess those RNAs that associate with Sm-proteins in total cell lysates via immunoprecipitation of the endogenous Sm-RNPs. In comparison, our study has three key advantages for assessing whether Sm-rings are, indeed, assembled on RNAs other than U snRNAs. First, we perform Sm-ring assembly and immunoprecipitations from cytoplasmic cell extracts—identifying Sm-protein associations in the environment Sm-ring assembly occurs within the cell. Restricting to the cytoplasm limits capture of RNAs that associate with Sm-proteins through the spliceosome and the pre-mRNA splicing process. Second, akin to U snRNA *in vitro* assembly assays, we supplemented cytoplasmic extracts with exogenous polyA-RNA and ATP to drive active assembly of new Sm-protein rings on RNAs that contain putative Sm-sites. Third, through informatics, we can remove the confounding effects of whether an Sm-site containing RNA also has sequence that can complement U snRNAs.

Sm-RIPs were performed in stringent conditions that have been shown to contain only snRNAs that have assembled high-salt and heparin-resistant Sm-rings [50]. Additionally, several lines of evidence support that the Sm–RNA interactions we observe on mRNAs are indeed authentic. We show that association between Sm-proteins and Sm-site containing mRNAs is ATP, SMN, and Sm-site-dependent, thus conforming to the established rules for Sm-ring assembly on U snRNAs (Figs 3D and E, 4A–C, and 6E–H, and K, and Supplementary Figs S12C, S23, B, D, E, and F, and S24B, C, E, and F). Furthermore, ATP-dependent Sm-association is independent of sequence that could facilitate U snRNP-binding (Fig. 4F–H and Supplementary Figs S15B–H, 19, and 20). We validate ATP, Sm-site, and SMN dependence using *in vitro* cytoplasmic Sm-ring assembly reactions on NDUFB6, SECISBP2l, Hif1a, and Cul5, mRNA 3′UTRs, using two different anti-Sm antibodies, providing evidence these mRNAs can indeed accept Sm-rings following established mechanistic rules for Sm-assembly of snRNAs (Fig. 6E–H and K, and Supplementary Figs S23B, D, and F, and S24). Next, Sm-rings assembled on four out of six newly identified targets (NDUFB6, SECISBP2l, Hif1a, and Cul5, mRNA 3′UTRs) are stable following a 2-M urea treatment (Fig. 6D–J and Supplementary Fig. S23, right), a higher stringency condition that favors an assembled Sm-ring by destabilizing RNA–RNA interactions [30, 50, 97–99]. Not only are the novel Sm-mRNP interactions we report captured with the Y12 anti-Sm antibody, but they are also captured with an antibody specific to SmB/B’/N (Figs 5A–C and 6K, and Supplementary Figs S23 and S24). Lastly, snRNAs are globally depleted in active Sm-ring assembly reactions incubated with polyA-RNA and ATP, indicating polyA-RNAs are actively competing with snRNAs for Sm-proteins (Fig. 4D). That these active, newly assembled Sm-mRNPs are stable following treatment with 2-M urea and in 1-M NaCl washing conditions strongly suggests an Sm-ring-like interaction.

These data invoke a model by which RNAs containing Sm-sites are the substrates of the SMN complex. Sm-proteins are pre-assembled in blocks composed of SmF/SmE/SmG, SmB(B’/N)/SmD3, and SmD1/SmD2 by PRMT5 complex and pICLn [5, 12, 14, 15, 121]. This sequestration kinetically traps spontaneous Sm-ring assembly from occurring within the cell. Interaction with the SMN-complex and the corresponding RNA substrates facilitates the specific assembly of the Sm-ring in a single-nucleotide to single Sm-protein reaction: first adding the pre-assembled Sm subcore of SmF/SmE/SmG and SmD1/SmD2, and final ring formation with the addition of SmB(B’/N)/SmD3 [5, 12, 15]. This reaction is highly specific for the Sm-site and ATP-dependent in cytoplasmic cell extracts [21, 23, 48–50]. ATP-dependent and SMN-dependent capture of NDUFB6, Hif1a, and Cul5 with the SmB/B’/N antibody following treatment with 2-M urea strongly suggests stable ring formation on these RNAs (Fig. 6E, G, H, and K). It is our view, supported by data herein, that the known SMN Complex machinery assembles Sm-rings on RNAs containing Sm-sites, arbitrary of the class of RNA or purpose. We specifically show this for mRNAs, as Sm-sites were most abundantly found in mRNA 3′UTRs, but do not suggest this as an exclusivity.

The efficiency of Sm-ring assembly on canonical Sm-site mRNAs is weaker than observed for U4 snRNA (Fig. 6D–H and Supplementary Figs S23 and S24). However, the efficiencies of Sm-ring assembly can be different even between snRNAs [50]. Further, the reported assembly of U7 snRNA required ∼2.4 times more cytoplasmic extract and 5 times more U7 snRNA than required for U1 snRNA to reach similar detection levels [30]. The difference in Sm-ring assembly efficiencies in these reactions may be related to how long it takes Gemin3 RNA-helicase activity to make the Sm-site accessible for Sm-ring deposition [51]. For instance, mRNAs, with their longer sequence, may adopt more complex or diverse secondary structures requiring more time to open the Sm-site by Gemin3. Additionally, Sm-ring assembly on an mRNA would be expected to compete with the myriad other cytoplasmic proteins and processes regulating mRNAs—including decay, stability, trafficking, and translation—that are present in the assembly reactions. Further, it is possible that U snRNAs are uniquely equipped to evade these processes—with their compact size, structure, and base-modifications—as a result of the necessity to expedite their import into the nucleus to perform their essential functions in the spliceosome and histone pre-mRNA processing. Despite weaker efficiency of assembly, the increase in signal over noise for Sm-site-containing mRNAs upon treatment with 2-M urea/5-mg/ml heparin strongly suggests assembly of a stable Sm-mRNP indicative of an Sm-ring (Supplementary Fig. S23).

Curiously, a fraction of Sm-site containing RNAs identified in the Sm-RIP experiments, including the KIF5B-3′UTR and Rem2-mRNA, do not exhibit both ATP or Sm-site dependence (Supplementary Fig. S23CG). Though the KIF5B 3′UTR shows an Sm-site dependent association, stable under the stringent IP conditions, it does not show an ATP-dependent increase for the wild-type RNA (Supplementary Fig. S23C). Among the various reasons for ATP and/or Sm-site independence, one possibility is that some of these sites may resemble recently reported sites that can receive Sm-rings in an ATP-independent manner [51]. This report provided evidence that U snRNAs predicted to form secondary structures across the Sm-site require ATP and the DEAD-box helicase Gemin3 to be imported into the nucleus, whereas those mutated to reduce Sm-site folding could be imported into the nucleus even when Gemin3 abundance was reduced [51]. Additionally, as discussed above, our noncanonical Sm-site definition may be comprised of both *bona fide* Sm-sites, as well as false Sm-sites, potentially explaining why Rem2-mRNA did not show Sm-site or ATP-induced Sm-ring assembly (Supplementary Fig. S23G). It is also possible that *in vitro* transcribed mRNAs used in Sm-ring assembly reactions may be lacking certain base-modifications that may be present in the polyA-RNA libraries used to perform the modified Sm-ring assembly reactions. While it is currently unknown if base-modifications affect Sm-ring assembly, they can influence RNA secondary structure [122, 123], which can in turn affect Sm-ring assembly [51]. Lastly, the prior proposed mechanisms involving base-pairing between assembled U snRNPs and a target RNA also remains a possibility for ATP and/or Sm-site independent association between Sm-proteins and RNAs [11]. Nonetheless, given the specificity prediction of an Sm-site has on driving enrichment with Sm-proteins, our data suggests the Sm-site is a main contributor to Sm-association for our Sm-site containing mRNAs.

### Impact of Sm-ring assembly on expression of Sm-site containing mRNAs

Although it is largely unknown how SMN-deficiency results in SMA, it is quite clear that reduced SMN in cell or animal models of SMA results in the reduction of U snRNP assembly [50, 52, 124–128], resulting in downregulation of assembly reactants—U snRNAs and Sm-protein mRNA—whilst maintaining a constant level of Sm-protein expression [9, 52, 107]. Furthermore, correction of the SMA phenotype strongly correlates with an increase in SMN protein expression and improved U snRNP assembly [52, 124, 127–129]. In congruence with these studies, we show that RNAs, in particular, mRNAs predicted to contain Sm-sites are globally reduced in abundance in mouse models of SMA as compared to other RNAs (Fig. 7A). Additionally, we show that 95% of the downregulated transcripts shared across the three independent datasets contain Sm-sites (Fig. 7D). Indeed, canonical Sm-site containing transcripts downregulated in these datasets are enriched in anti-Sm-RIPs (Fig. 7E), providing further evidence of a correlation between Sm-site containing mRNAs, Sm-ring assembly, and SMN-deficiency. Therefore, our data supports a proposal that SMA results in reduced capacity of SMN to assemble Sm-rings on both snRNAs and mRNAs, resulting in their reduced abundance. Moreover, such a mechanism could contribute along with other proposed mechanisms of SMA pathogenesis including altered pre-mRNA splicing. Thus, the critical targets in SMA could be both mis-spliced and downregulated.

How does the U-rich Sm-site, prominently found in the 3′UTRs of long mRNAs, and the binding of Sm-proteins to these sites, impact expression of these mRNAs? One possibility is that assembly of Sm-rings onto these mRNA interplays with other RNA-binding proteins (RBPs) that bind 3′UTRs to regulate gene expression [94, 95]. Within the RBPDB, an experimentally validated collection of RBPs and their binding sites [92], we found that a handful of RBPs are annotated to bind elements that contain the Sm-site sequence. These RBPs include SFRS1, Tia1, ELAVL1/4, gld-1, pos-1, ZFP36, and Cus2. Two of these—SFRS1 and Tia1—are splicing factors that bind U-rich elements in introns to affect pre-mRNA splicing [130, 131]. Cus2 interacts with the U2 snRNA [132]. The rest—ELAVL1/4, gld-1, pos-1, and ZFP36—promote stability of their target mRNAs [130, 133–140], some by inhibiting target mRNA translation [135]. The most interesting of these are ELAVL1/4, also known as HuR and HuD. HuD is specifically expressed in neurons and it’s overexpression has been shown to partially suppress the SMA phenotype in zebrafish and mouse SMA models, and neurite outgrowth defects in SMN-deficient cells [71, 72, 78]. While the mechanism of SMA suppression by HuD has primarily focused on SMN binding [71, 72, 78], we suggest that a potential mechanism for HuD-mediated suppression of SMA may occur by stabilizing Sm-site containing mRNAs that do not recieve an Sm-ring as a result of reduced Sm-ring assembly by the SMN complex. Another possibility stems from observations that Sm-ring assembly occurs on immature yeast telomerase RNA, but to then be replaced by the Lsm-ring in mature telomerase [79]. It is possible that such a mechanism could be adopted by Sm-site containing mRNAs and other RNAs, whereby Sm proteins may assist or antagonize Lsm-ring assembly within 3′UTRs.

SMN-deficiency has been shown to affect the trafficking of mRNAs in motor neurons. Our findings indicate that Sm-site containing RNAs, and Sm-associated RNAs, are almost exclusively found in motor neuron somas, challenging the hypothesis that the Sm-site is used to traffic mRNAs to be locally translated at axon terminals (Supplementary Fig. S26A and B). However, this could raise a new possibility that the Sm-site acts as a signal for retention of the mRNA in the soma. Additionally, our finding that RNAs associated with COPI-mediated intracellular vesicle trafficking are also associated with Sm-proteins, and that the majority of these are predicted to contain Sm-sites (Supplementary Fig. S26C and E), suggests yet another mRNA transport mechanism involving 3′UTR Sm-sites. It has previously been shown that over-expression of alpha-COP, the large subunit of the COPI particle, can partially suppress the SMA phenotype in zebrafish, mice, and the neurite outgrowth defects in SMN-deficient neuronal cell cultures [116, 117, 141]. However, as in the case of HuD, the mechanism for this suppression has only focused on alpha-COP association with SMN. Our data suggests a link between alpha-COP mRNA trafficking and Sm-site containing mRNAs. Could the Sm-site confer a signal to incorporate an mRNA into a COPI vesicle for intracellular trafficking, and might the partial suppression of the SMA phenotype by over-expression of alpha-COP occur by increasing the trafficking of these mRNAs?

In summary, we have dramatically expanded the list of RNAs that contain classical and putative Sm-sites and provided stringent criteria for determining Sm-ring assembly potential for these RNAs. Our experiments suggest a novel role for Sm-ring assembly in the regulation of cytoplasmic RNAs, and in particular mRNAs. Exactly how this regulation is controlled and the magnitude of its effect on the physiology of Sm-site-containing RNAs is an intriguing question. Furthermore, how this novel mechanism fits into the pathogenesis of SMN-deficiency in SMA and whether it directly ties SMN function to affected RNA processing events will be beneficial to future progress on understanding the disease.

## Supplementary Material

gkaf794_Supplemental_Files

## Data Availability

Data supporting findings for this study are available in this manuscript and associated Supplementary Data and Files. Raw sequencing data are uploaded to NCBI GEO278538. Custom scripts and master tables used for generating figures are available in Github (https://github.com/ajblatnik/sm_ring_assembly_mrna.git) GitHub - ajblatnik/sm_ring_assembly_mrna Contribute to ajblatnik/sm_ring_assembly_mrna development by creating an account on GitHub. github.com and Zenodo (DOI:10.5281/zenodo.15476098). Large Supplementary Files are also available in .tsv format at the same Github and Zenodo links.

## Notes

Present address: Department of Genetics and Genome Sciences, Case Western Reserve University School of Medicine, 2109 Adelbert Road, Cleveland, OH 44106, United States
