# Supplementary material for: Sm-site containing mRNAs can accept Sm-rings and are downregulated in Spinal Muscular Atrophy
Source: Nucleic Acids Res. 2025 Aug 18;53(15):gkaf794. doi: 10.1093/nar/gkaf794 (PMC12359035; doi:10.1093/nar/gkaf794)
Supplement: gkaf794_Supplemental_Files [file gkaf794_supplemental_files.zip › NAR-0320-C-2024_Revised_Supplementary_Figures_2025.07.28.docx]

**SUPPLEMENTARY FIGURES FOR:**

**Sm-site containing mRNAs can accept Sm-rings and are downregulated in Spinal Muscular Atrophy**

**AUTHORS**

Anton J. Blatnik III^1,^*, Manu Sanjeev^2^, Jacob Slivka^1^, Benjamin Pastore^1,3^, Caleb M. Embree^2^, Wen Tang^1,3^, Guramrit Singh^2,3^, Arthur H. M. Burghes^1,3^

^1^ Department of Biological Chemistry and Pharmacology, The Ohio State University Wexner Medical Center, 395 W. 12th Ave, Columbus, OH 43210.

^2^ Department of Molecular Genetics, The Ohio State University, 484 West 12th Avenue, Columbus, OH 43210.

^3^Center for RNA Biology, The Ohio State University, Columbus, OH 43210, USA

*Correspondence should be addressed to Anton J. Blatnik III. Email: anton.blatnik@case.edu.

Present Address: Anton J Blatnik III, Department of Genetics and Genome Sciences, Case Western Reserve University School of Medicine, 2109 Adelbert Road, Cleveland, OH 44106.

**SUPPLEMENTARY** **FIGURES
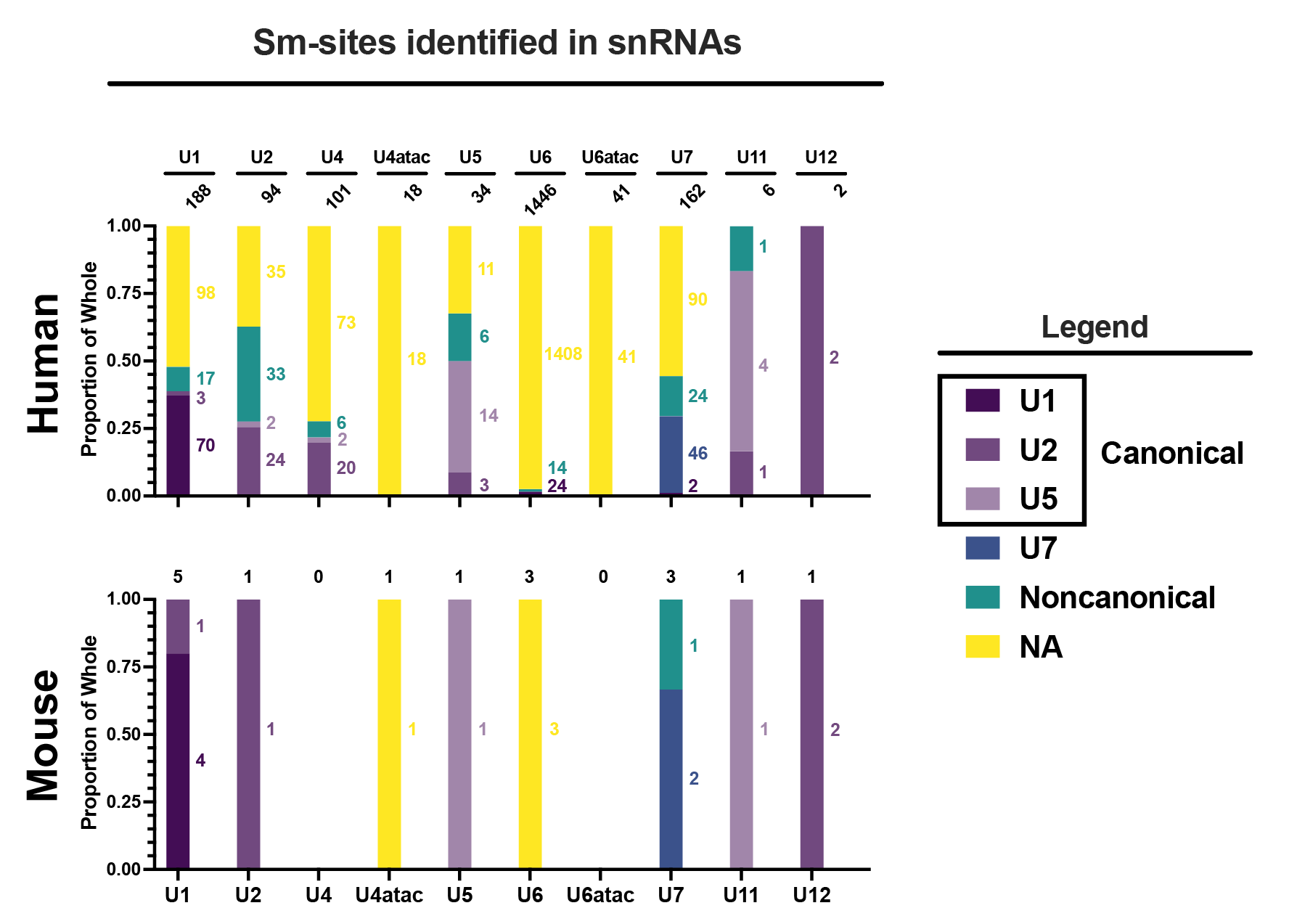
**

**Supplementary Figure 1:** **Breakdown of type of Sm-site detected in different annotated U snRNAs.** **(top)** for human genes, **(bottom)** for mouse genes. For all bar graphs, top numbers are the number of genes represented within the bar, side numbers are the values contributing to the bar for a given Sm-site, in color. Each bar corresponds to the annotation of snRNA—U1, U2, U4, U4atac, U5, U6, U6atac, U7, U11, and U12. Within each bare is a breakdown of each Sm-site type—U1, U2, U5, U7, Noncanonical or Absent. Of note, mouse snRNAs are very poorly annotated, with only a few variants, if any, defined for each type.

**Supplementary Figure 2:** **Breakdown of type of Sm-site detected in NCBI Refseq and Gencode human
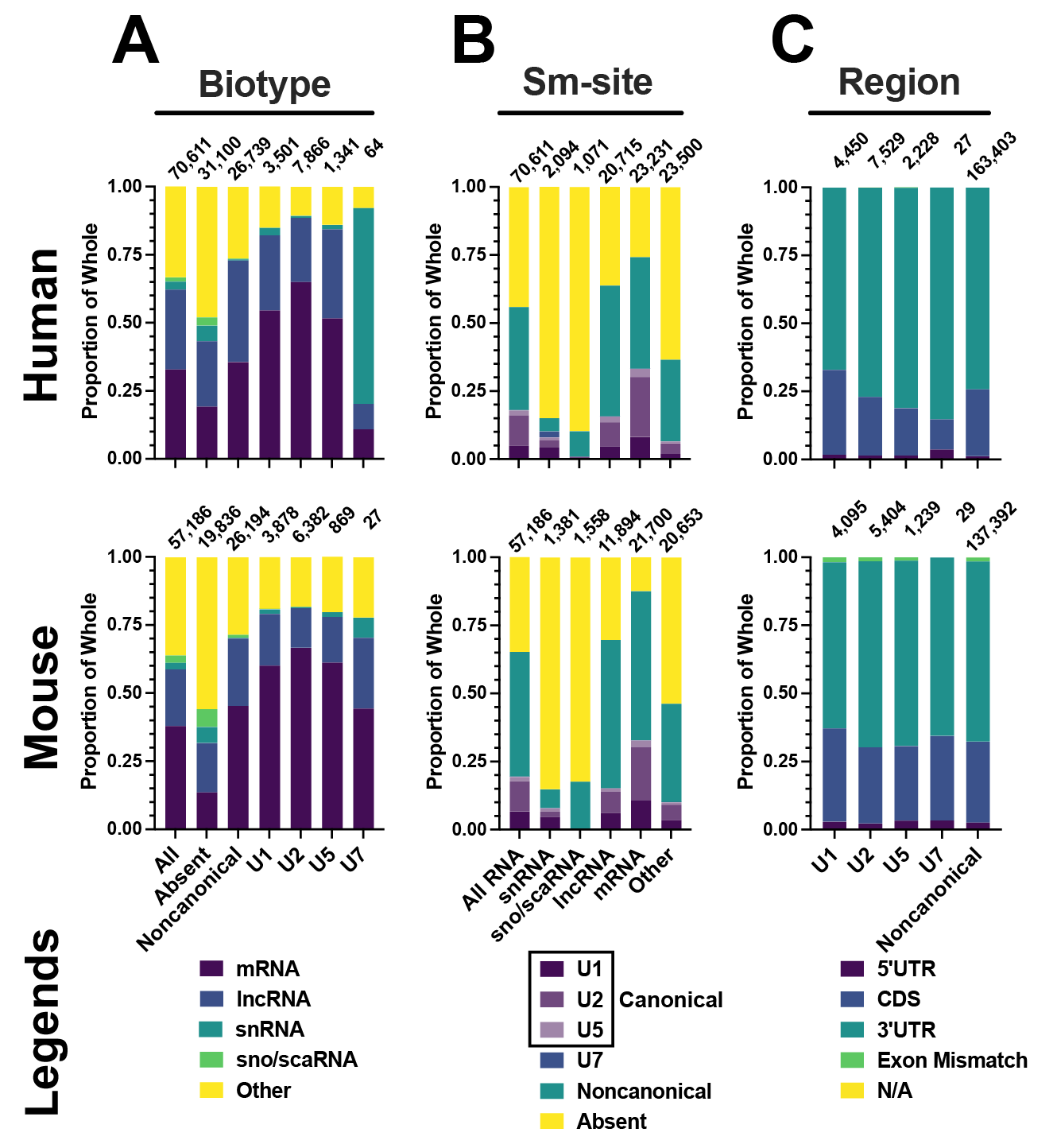
 and mouse transcriptomes.** **(top)** for human genes, **(bottom)** for mouse genes. For all bar graphs, top numbers are the number of genes represented within the bar. Data presented in **A**-**C** correspond to a single, unique transcript ID of a single, unique gene. **(A)** Proportional bar graph of RNA biotypes. **All** is a breakdown of the annotated genome. **Absent** are RNAs not predicted to contain an Sm-site. **Noncanonical** are those RNAs only predicted to contain noncanonical Sm-site sequences. **U1**, **U2**, **U5**, and **U7** are RNAs predicted to have a U1, U2, U5, or U7 Sm-site, but may have additional Sm-site sequences. **(B)** Proportional bar graph giving a breakdown of types of Sm-sites predicted in each of the following biotypes: All, snRNA, sno/scaRNA, lncRNA, mRNA, and Other. **(C)** Proportional bar graph depicting the region of an mRNA where Sm-sites are predicted.

**Supplementary Figure 3. Frequency of Sm-sites is mildly correlated with transcript or 3’UTR length.
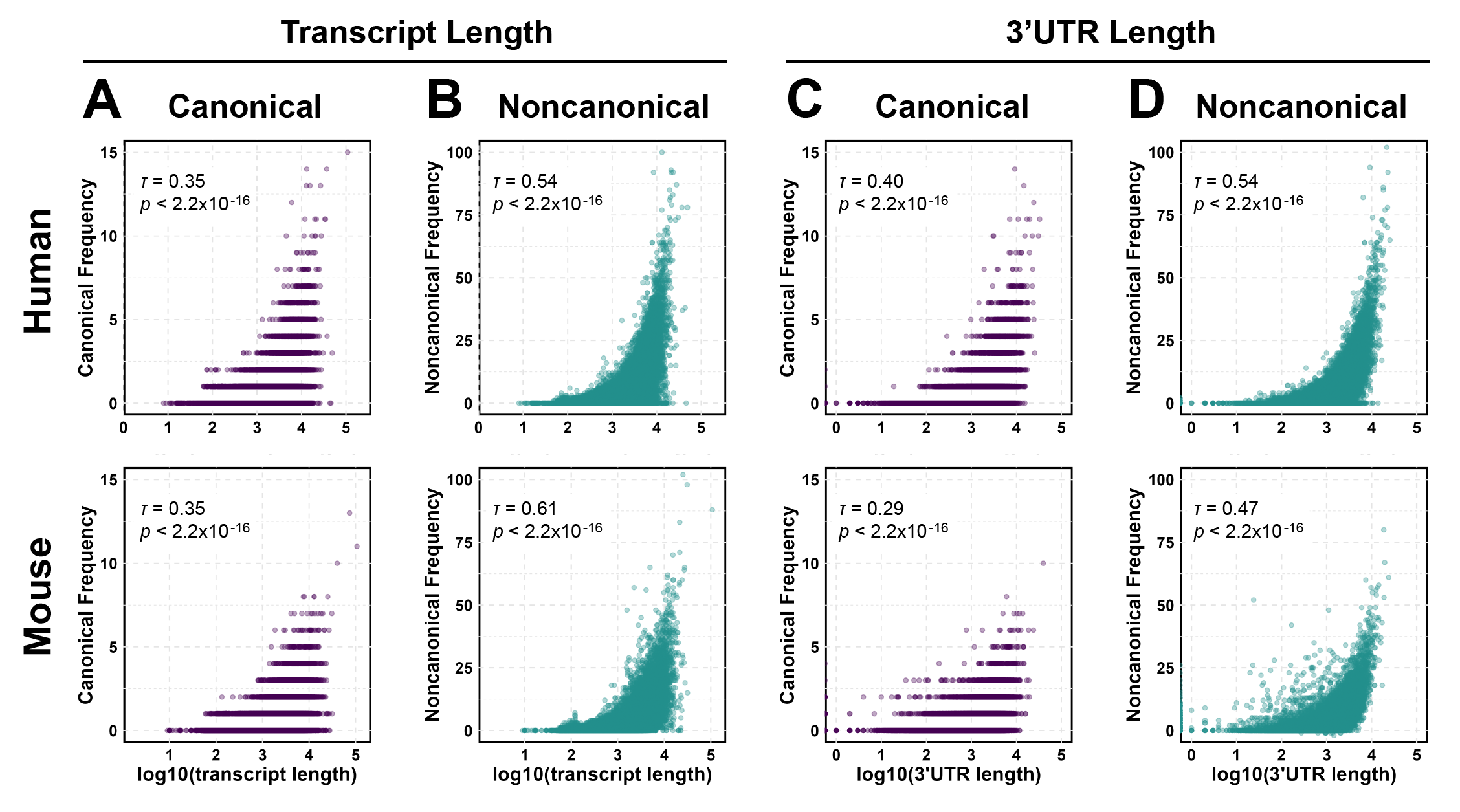
** **Top:** human genes, **Bottom:** mouse genes. For all bar graphs, top numbers are the number of genes represented within the bar. Data presented in **A-D** represent a unique transcript ID of a single, unique gene ID. **(A)** Scatter plot of the frequency of canonical Sm-sites predicted in a transcript vs the length of the transcript. *R* is the coefficient of a Kendall rank-ordered test. **(B)** Scatter plot of the frequency of noncanonical Sm-sites predicted in a transcript vs the length of the transcript. 𝜏 is the coefficient of a Kendall rank-ordered test. **(C)** Scatter plot of the frequency of canonical Sm-sites predicted in the 3’UTR of an mRNA vs the length of the mRNA 3’UTR. *R* is the coefficient of a Kendall rank-ordered test. **(D)** Scatter plot of the frequency of noncanonical Sm-sites predicted in the 3’UTR of an mRNA vs the length of the mRNA 3’UTR. 𝜏 is the coefficient of a Kendall rank-ordered test.

**Supplementary Figure 4: Modification of the standard Sm-ring assembly reaction to test Sm-protein
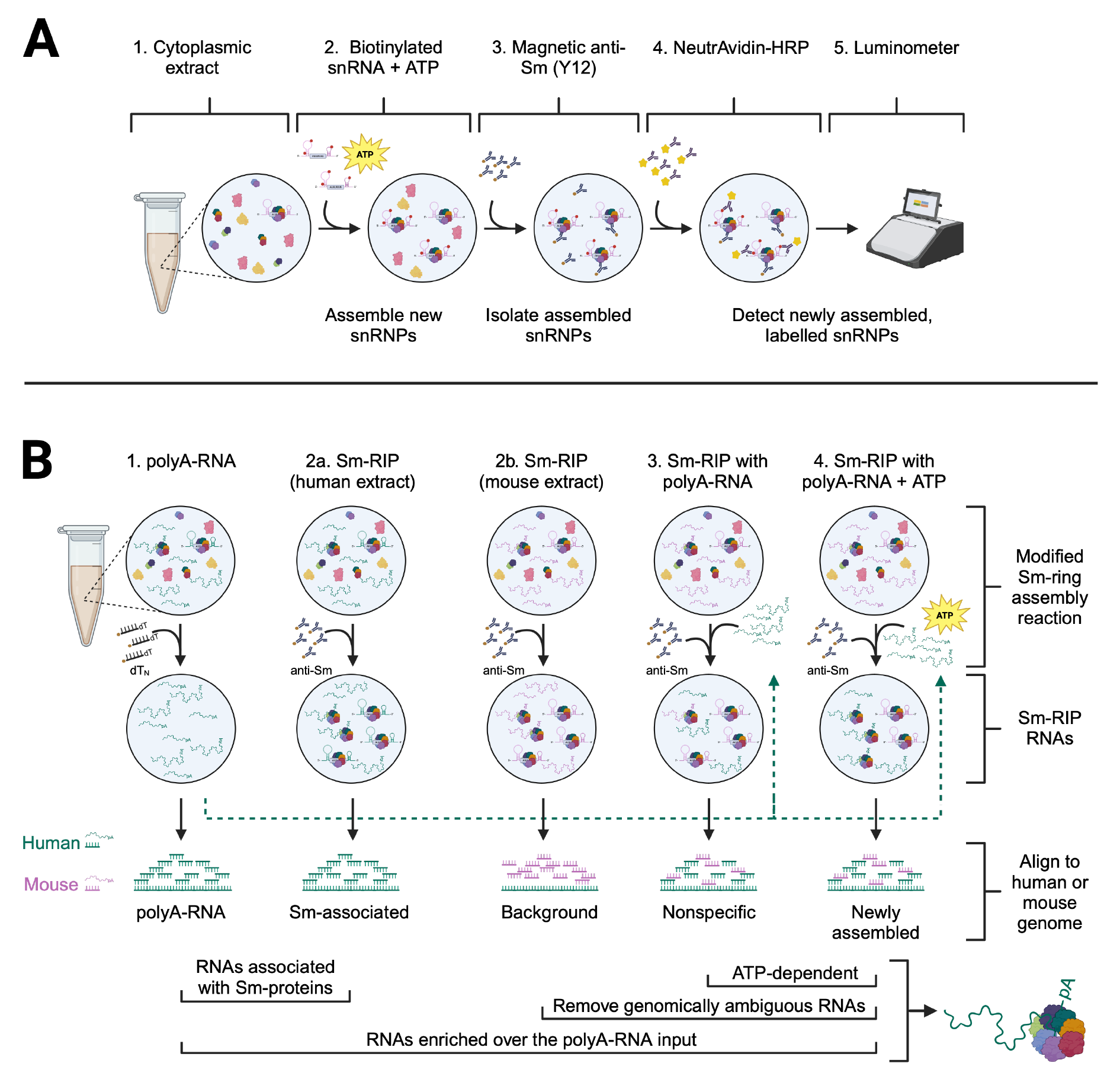
 ring assembly on polyA-RNAs.** **(A)** Schematic of the snRNP assembly reaction described by Wan *et al*. 1) a cytoplasmic cell extract is, 2) incubated with an *in vitro* transcribed, biotinylated human U4 snRNA and ATP. Newly assembled snRNPs are 3) enriched following an anti-Sm (Y12) immunoprecipitation and 4) detected using an NeutrAvidin-HRP antibody and 5) quantified using a luminometer. **(B)** Modified Sm-ring assembly reaction in which polyA-RNA is supplied in place of an *in vitro* transcribed snRNA. Green denotes an RNA or genome of human origin and pink denotes an RNA or genome of mouse origin. Four sample conditions were analyzed. 1) a polyA-RNA enriched library used as input for the Sm-ring assembly reactions and aligned to the genome of origin. 2) an anti-Sm (Y12) RNA immunoprecipitation (Sm-RIP) in cytoplasmic cell extract to identify those RNAs that associate with Sm-proteins under physiological conditions when aligned to the genome of origin (2a), and secondly to remove genomically ambiguous RNAs when aligned to the opposing species genome (2b). 3) an Sm-RIP in which polyA-RNA of one species is incubated with the cytoplasmic extract of the opposing species and aligned to the genome of polyA-RNA origin to identify RNAs that newly associate with Sm-proteins, but that are likely nonspecific. 4) an Sm-RIP in which polyA-RNA of one species is incubated with the cytoplasmic extract of the opposing species and ATP, which aligned to the genome of polyA-RNA origin will identify RNAs with newly assembled Sm-rings. Comparisons outlined underneath conditions were used to identify the candidate polyA-RNAs that most likely recieve an Sm-ring. The candidate should be show an ATP-dependent enrichment, it should be enriched over the polyA-RNA input library, and the RNA should associate with Sm-proteins under physiological conditions. Lastly, reads aligning to both human and mouse genomes (genomically ambiguous) can be removed, as these would be considered false-positives. Created in BioRender (Blatnik, A. (2025) <https://BioRender.com/ko8o1mi>).


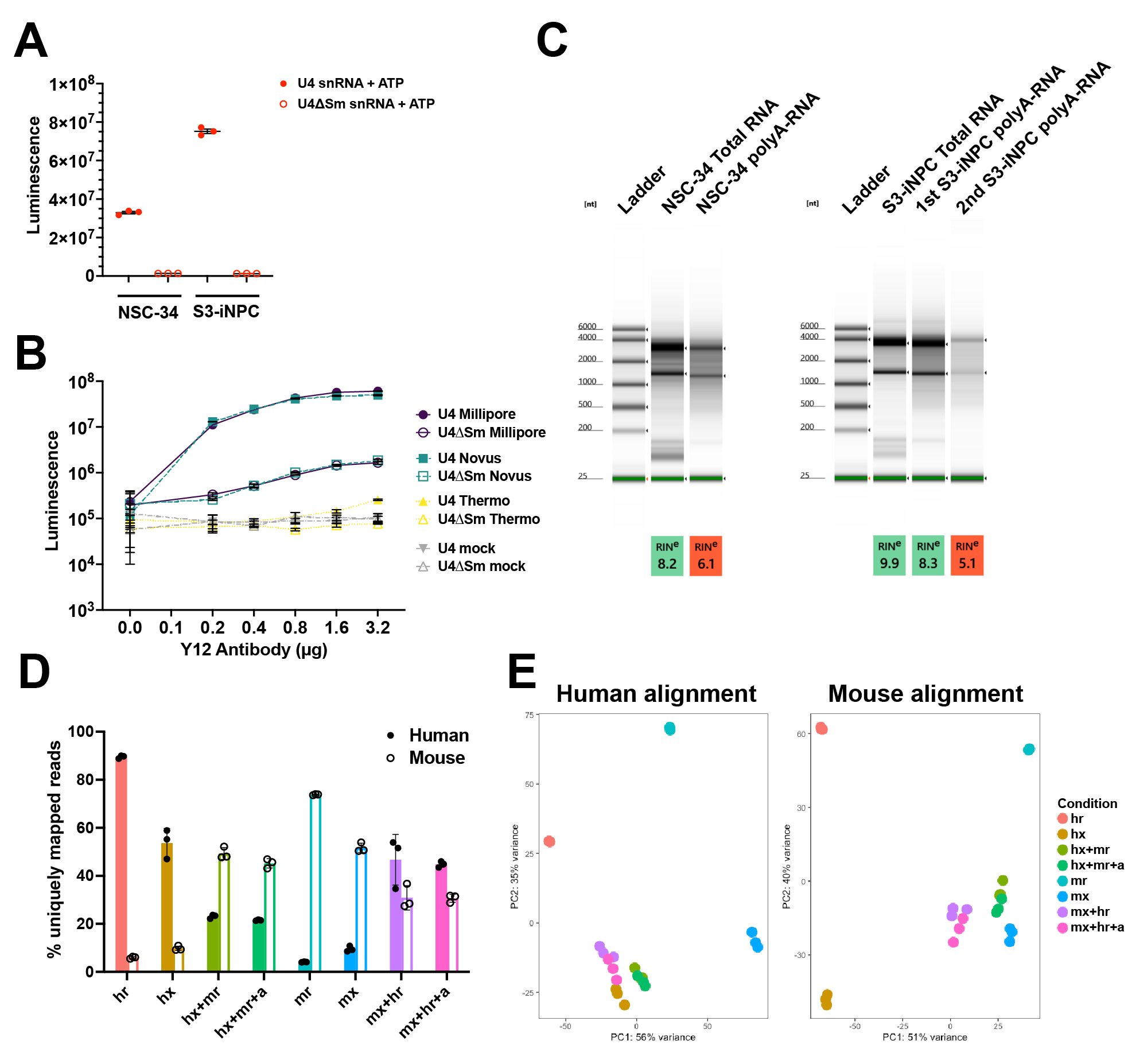
**Supplementary Figure 5:** **Controls and validation of the experimental outline.** **(A)** snRNP assembly capacity in cytoplasmic cell extracts used to perform Sm-assembly specific RNA immunoprecipitations. The amount of cytoplasmic cell extract was adjusted proportionally to yield equivalent snRNP assembly capacity. **(B)** snRNP assembly reactions performed to select manufacturer of anti-Sm Y12 antibody. A single lot of the Novus Y12 antibody was used for all subsequent experiments at a ratio of 1.6 µg antibody to 15 µL Dynabeads-Protein G. **(C)** RNA-high sensitivity TapeStation gel image comparing Total RNA to oligo-dT (polyA-RNA) enriched RNA used in Sm-assembly specific RNA immunoprecipitations. Isolation of S3-iNPC polyA-RNA required two rounds of oligo-dT purification. Reduced RIN was used to as a proxy for removal of non-polyA-RNAs. **(D)** Percent uniquely mapped, STAR aligned reads for each sequenced libraries. Alignment of the human GRCh38 genome is depicted by solid shapes and mouse GRCm39 genome by the open shapes. The designation of ‘h’ or ‘m’ identifies the species of the following condition: ‘r’ polyA-RNA, ‘x’ cytoplasmic extract. ‘a’ indicates the addition of ATP. Therefore, mx+hr+a is a mouse cytoplasmic extract incubated with human polyA-RNA and ATP. **(E)** Principal component analysis for each sequencing library color coded by the sample condition. Naming is the same as in (D).

**
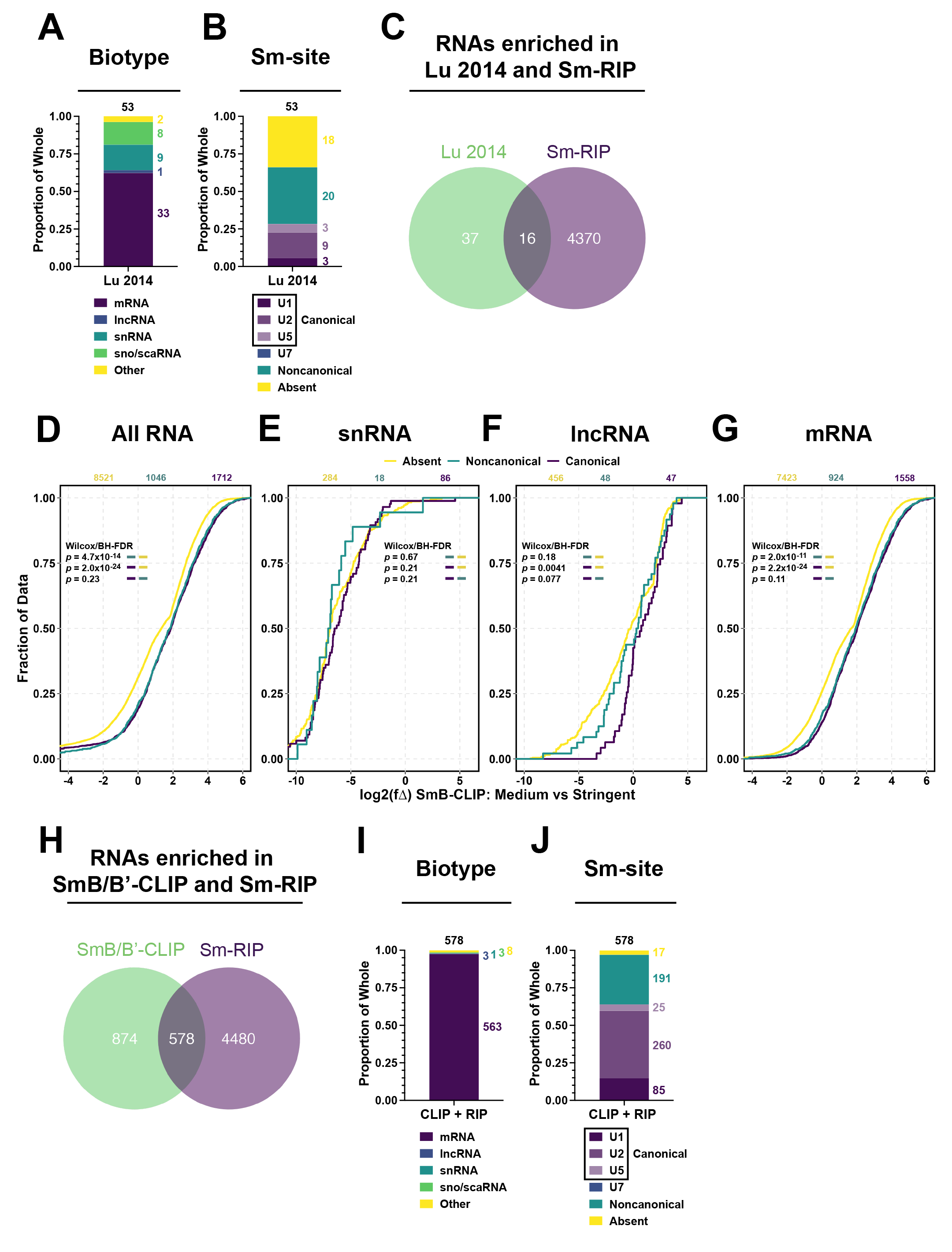
Supplementary Figure 6: Sm-site containing mRNAs are enriched in previous Sm-RIP and anti-SmB/B’-CLIP-Seq data.** For **(ABIJ)**, values above bars indicate the number of genes contributing to the plots. Numbers in color to the sides of bars indicate the number of genes contributing to the specified group within the bar. **(A)** Proportional bar graph of the RNA biotypes for gene products enriched with Sm-proteins by Lu *et al* Genome Biology 2014. **(B)** Proportional bar graph giving a breakdown of types of Sm-sites predicted in RNAs enriched with Sm-proteins by Lu *et al* Genome Biology 2014. **(C)** Venn diagram depicting the overlapping gene products between different Sm-association conditions. **Lu 2014** are those RNAs found to be enriched with Sm-proteins within the Lu *et al* Genome Biology 2014 study. **Sm-enriched** are RNAs physiologically associated with Sm-proteins—log2(f∆) ≥ 0.6, *padj* < 0.05, between anti-Sm-RIP condition and the polyA-RNA sequencing conditions in Figure 2CD. **(D-G)** Cumulative distribution function plots comparing the Medium and Stringent wash conditions from the anti-SmB-CLIP-Seq data published by Briese *et al* 2020 [Briese Nat Struct Mol Biol 2020]. CDFs included plot **All RNA (D)**, only **snRNA (E)**, only **lncRNA (F)**, or only **mRNA (G)** delineating by Sm-site prediction—**Absent** (yellow), **Noncanonical** (green), **Canonical** (purple). Values above CDFs indicate the number of genes plotted for each condition in color. Wilcoxon Rank Sum Tests with Continuity Correction and Benjamini-Hochberg False Discovery Rate corrections for multiple testing were used to calculate adjusted *p-values* for the left color being greater than the right color are provided in the upper lefthand corner of the graphs. **(H)** Venn diagram depicting the overlapping gene products between different Sm-association conditions. **SmB-CLIP** are those RNAs comprised of the highest quartile log2(f∆) between the Medium and Stringent wash conditions as described by Briese *et al* 2020. **Sm-enriched** are RNAs associated with Sm-proteins—log2(f∆) ≥ 0.6, *padj* < 0.05, between anti-Sm-RIP condition and the polyA-RNA sequencing conditions in Figure 2CD. **(I)** Proportional bar graph of the RNA biotypes for gene products shared between the SmB-CLIP and Sm-enriched conditions **(CLIP + RIP)**. **(J)** Proportional bar graph giving a breakdown of types of Sm-sites predicted in RNAs shared between the SmB-CLIP and Sm-enriched conditions **(CLIP + RIP)**.

**Supplementary Figure 7. Sm-site containing RNAs are specifically enriched with Sm-proteins.
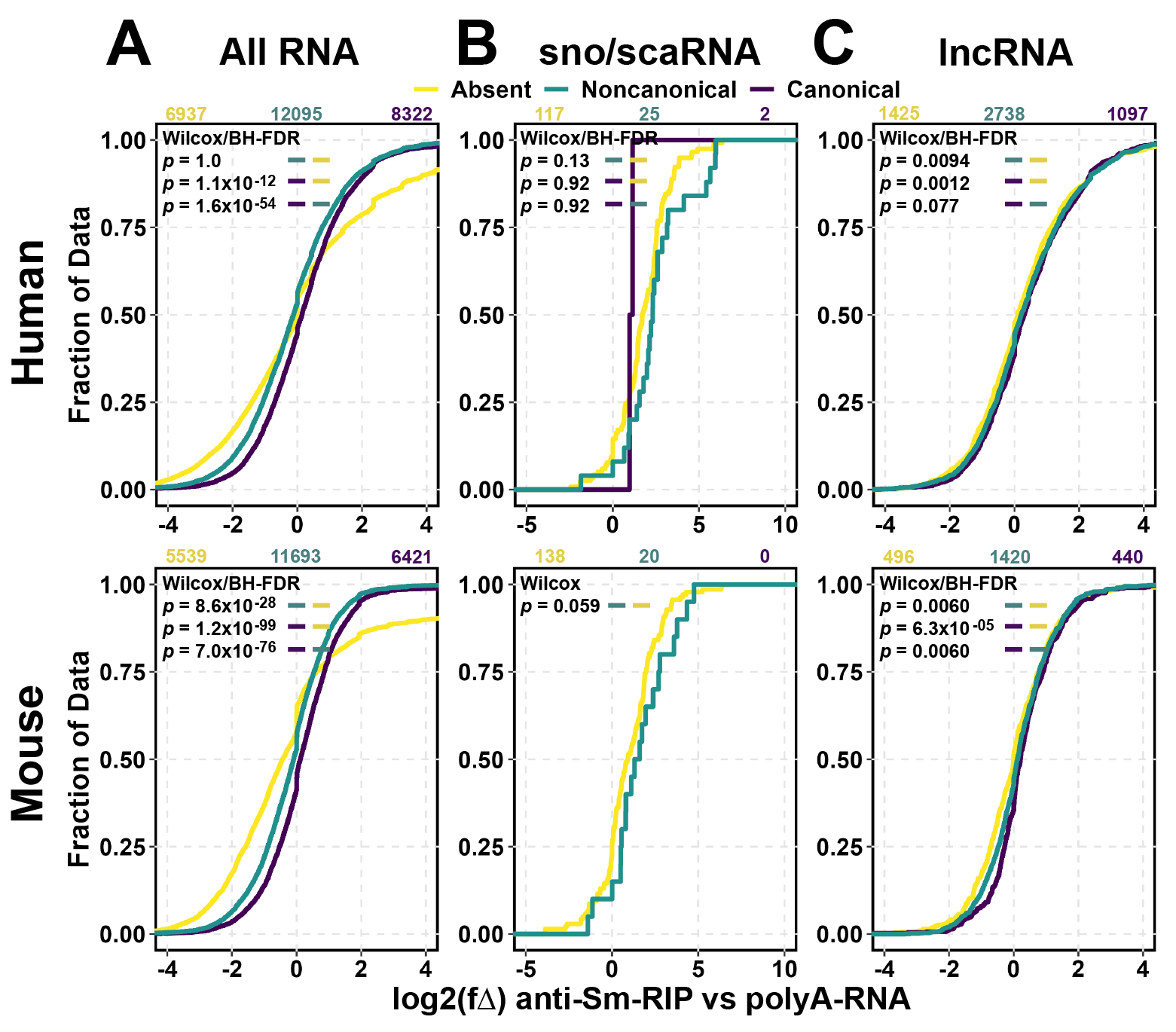
** Cumulative distribution plots for given RNA biotypes, plotting the log2 fold change (log2(f∆)) by increasing value. log2(f∆) was calculated between the anti-Sm-RIP vs polyA-RNA conditions. U7 Sm-site-containing RNAs were removed from analysis. **(A)** All RNA, **(B)** sno/scaRNA, **(C)** lncRNA. Colors designate whether an Sm-site is **Absent** (yellow), **Noncanonical** (green), or **Canonical** (purple). Values above CDFs indicate the number of genes plotted for each condition in color. Wilcoxon Rank Sum Tests with Continuity Correction and Benjamini-Hochberg False Discovery Rate corrections for multiple testing were used to calculate adjusted *p-values* for the left color being greater than the right color are provided in the upper lefthand corner of the graphs.


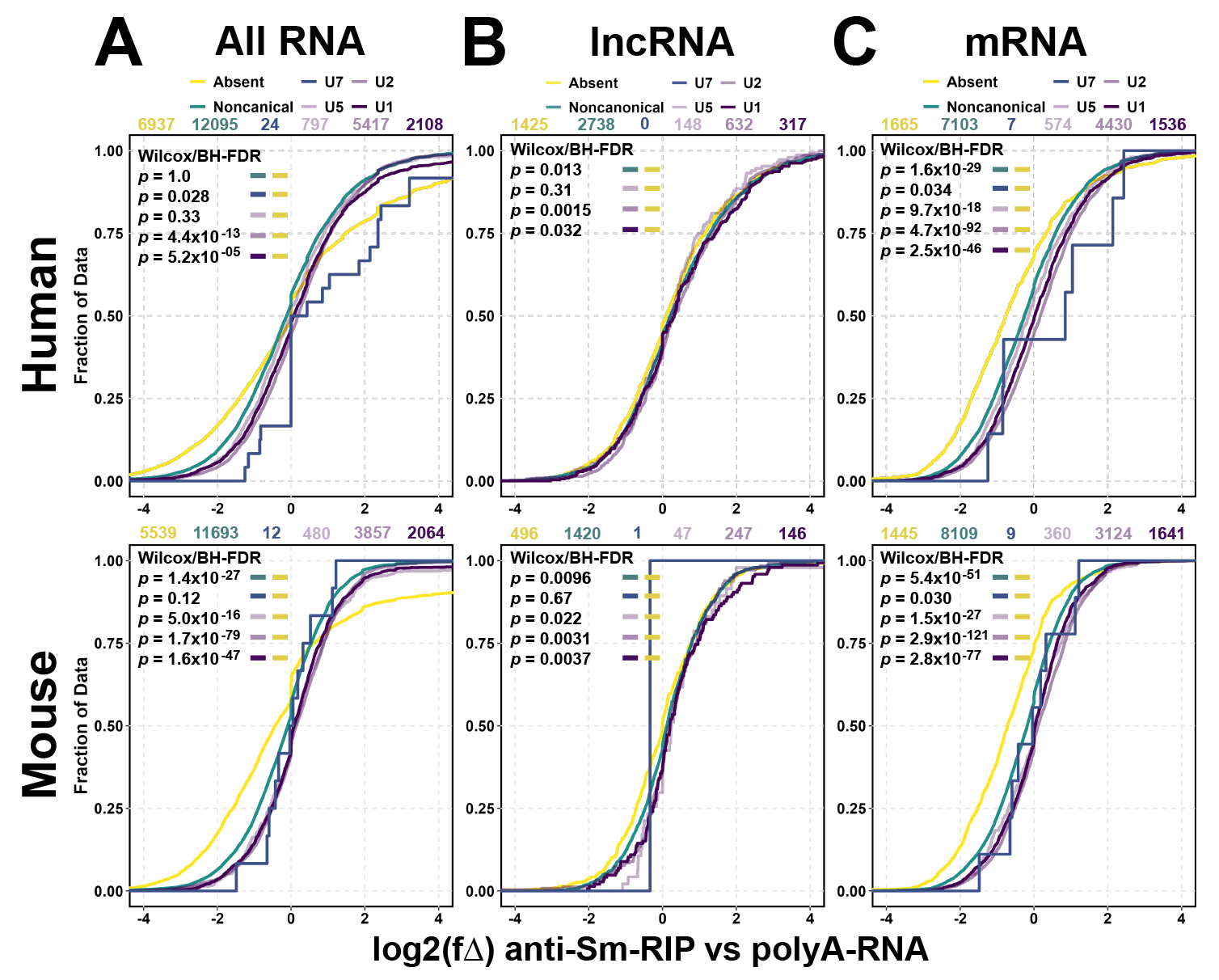


**Supplementary Figure 8: Type of Sm-site correlates with further enrichment with Sm-proteins.** Cumulative distribution plots for given RNA biotypes, plotting the log2 fold change (log2(f∆)) by increasing value. log2(f∆) was calculated between the anti-Sm-RIP vs polyA-RNA conditions. (A) All RNA, (B) lncRNA, (C) mRNA. Colors designate whether an Sm-site is Absent (yellow), Noncanonical (green), U7 (blue), or U1, U2, or U5 (purple, darkest to lightest). Values above CDFs indicate the number of genes plotted for each condition in color. Wilcoxon Rank Sum Tests with Continuity Correction and Benjamini-Hochberg False Discovery Rate corrections for multiple testing were used to calculate adjusted *p-values* for the left color being greater than the right color are provided in the upper lefthand corner of the graphs.

**Supplementary Figure 9:** **The frequency of Sm-sites in an RNA correlates with Sm-protein enrichment.
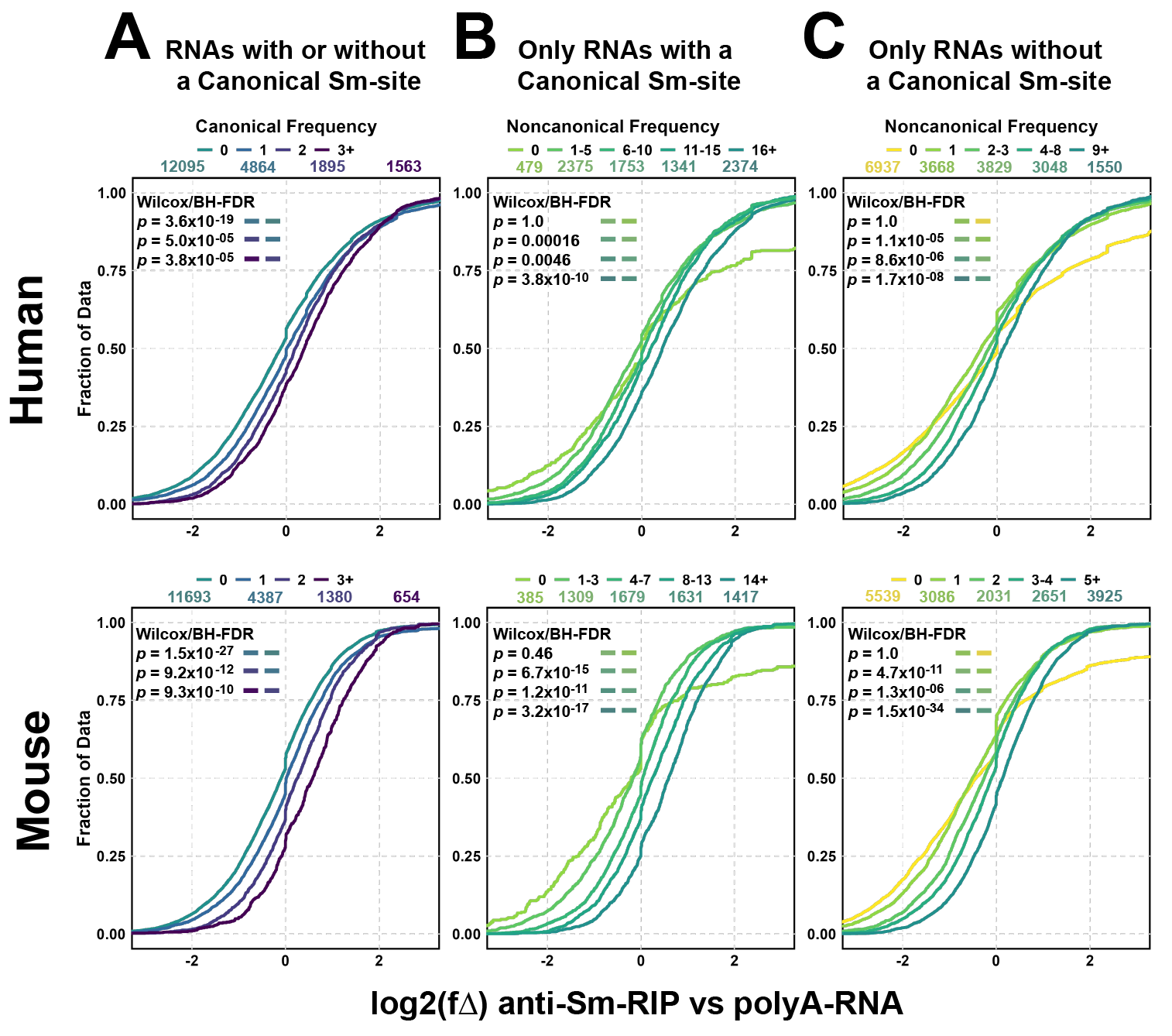
 (top)** for human analysis and **(bottom)** for mouse analysis. For each cumulative distribution plot, log2 fold change (log2(f∆)) between the anti-Sm-RIP and polyA-RNA conditions are plotted in increasing order, delineated by the frequency of Sm-sites predicted for the product of the gene. Values above CDFs indicate the number of genes plotted for each condition in color. Wilcoxon Rank Sum Tests with Continuity Correction and Benjamini-Hochberg False Discovery Rate corrections for multiple testing were used to calculate adjusted *p-values* for the left color being greater than the right color are provided in the upper lefthand corner of the graphs. **(A)** Cumulative distribution plot delineating the frequency of canonical Sm-site prediction in an mRNA transcript by log2 fold change (log2(f∆)) in anti-Sm-RIP vs the polyA-RNA transcriptome. mRNAs predicted to solely contain noncanonical Sm-sites are not plotted in this graph. **(B)** Cumulative distribution plot of only mRNAs predicted to contain at least one canonical Sm-site, delineating the frequency of noncanonical Sm-sites predicted within the mRNA transcript, against the log2 fold change in anti-Sm-RIP vs the polyA-RNA transcriptome. **(C)** Cumulative distribution plot of mRNAs predicted to solely contain noncanonical Sm-sites, delineating the frequency of noncanonical Sm-sites predicted within the mRNA transcript, against the log2 fold change in anti-Sm-RIP vs the polyA-RNA transcriptome.

**Supplementary Figure 10: Transcript or 3’UTR length is not a predictor of anti-Sm RIP enrichment.** **(top)
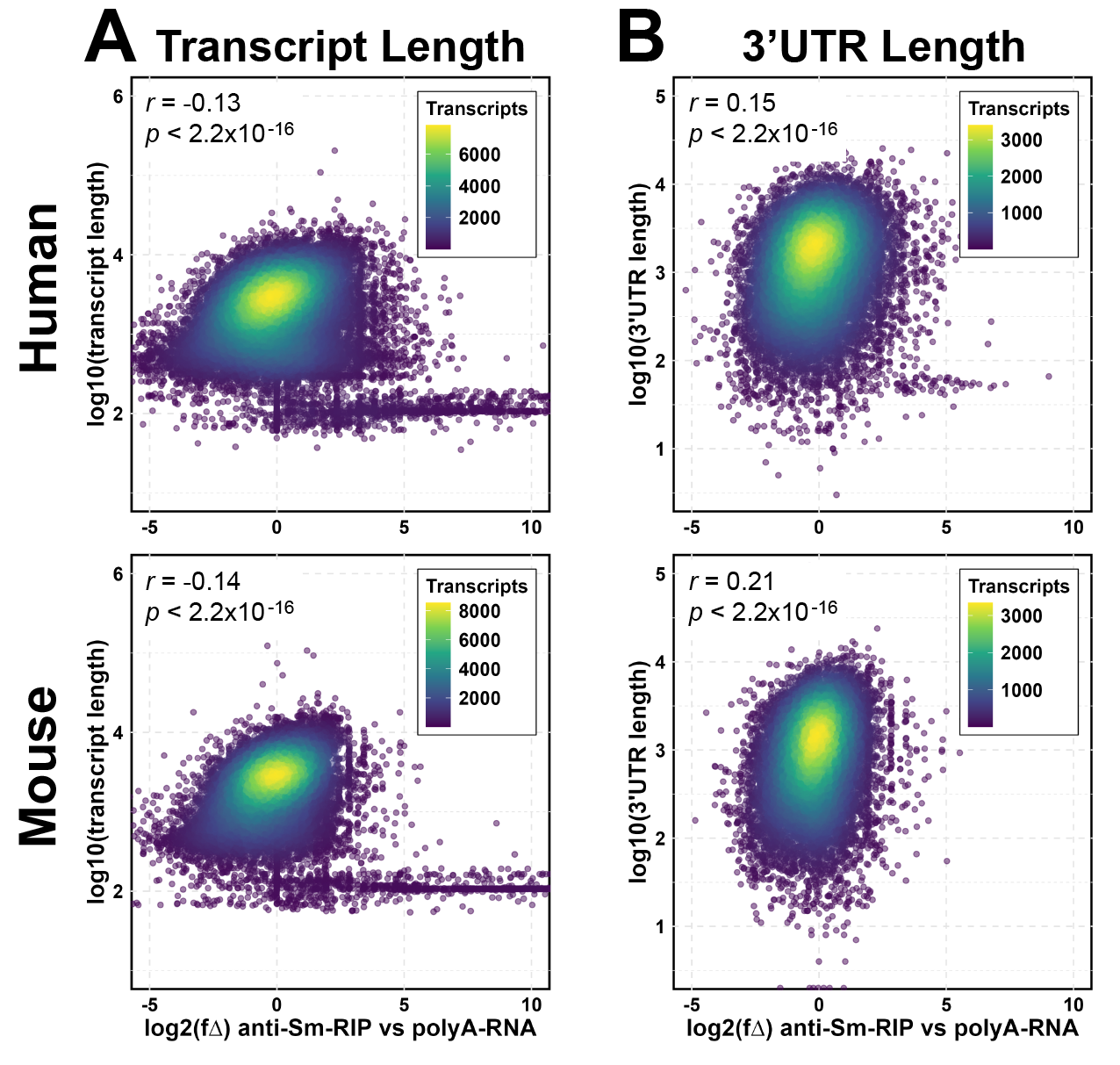
** for human analysis and **(bottom)** for mouse analysis. **(A)** log10 of transcript length plotted against log2 fold change between anti-Sm-RIP and the polyA-RNA transcriptome. *r* is a Pearson correlation coefficient. **(B)** log10 of 3’UTR length plotted against log2 fold change between anti-Sm-RIP and the polyA-RNA transcriptome. *r* is a Pearson correlation coefficient.

**Supplementary Figure 11: Noncoding RNAs containing Sm-sites are also enriched in anti-Sm-RIPs in
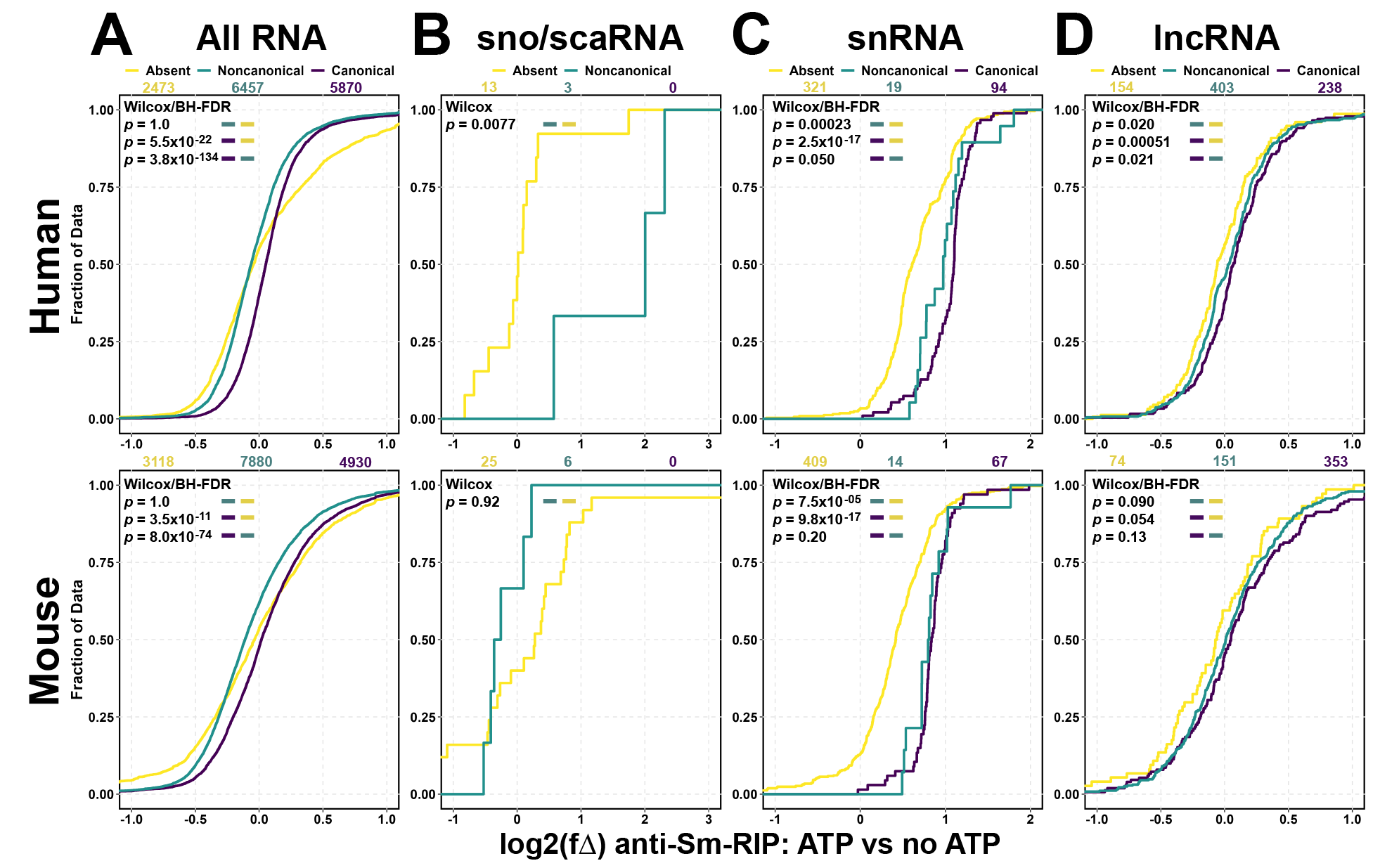
 an ATP-dependent manner.** For **(A-D)**, **(top)** for human analysis and **(bottom)** for mouse analysis. **(A-C)** Cumulative distribution plots of all RNA **(A)**, only sno/scaRNA **(B)**, snRNA **(C)**, or lncRNA **(D)** delineating by Sm-site prediction—**Absent** (yellow), **Noncanonical** (green), **Canonical** (purple). Values above CDFs indicate the number of genes plotted for each condition in color. Wilcoxon Rank Sum Tests with Continuity Correction and Benjamini-Hochberg False Discovery Rate corrections for multiple testing were used to calculate adjusted *p-values* for the left color being greater than the right color are provided in the upper lefthand corner of the graphs.

**Supplementary Figure 12: Type of Sm-site correlates with further enrichment following ATP addition
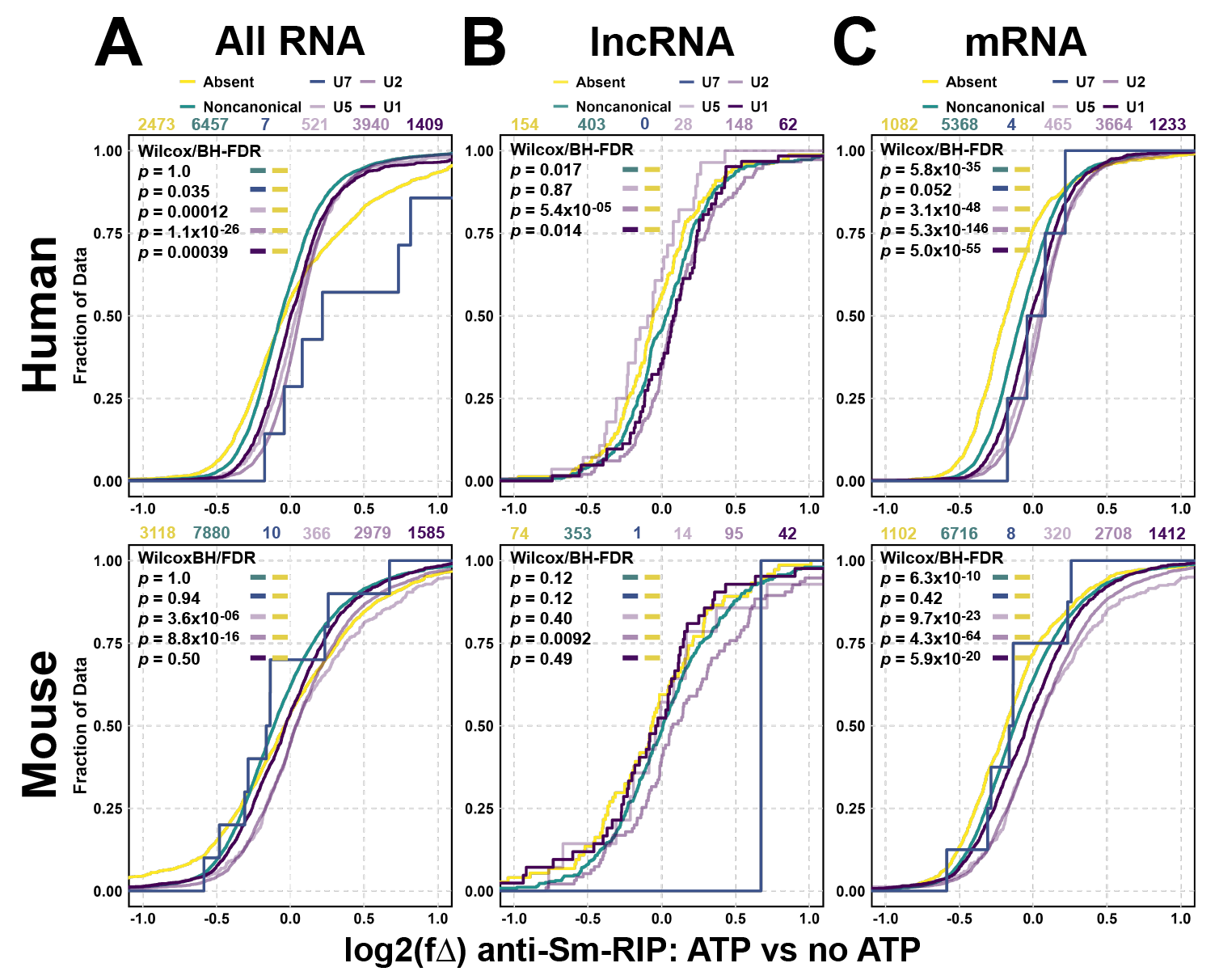
 and anti-Sm-RIP.** Cumulative distribution plots for given RNA biotypes, plotting the log2 fold change (log2(f∆)) by increasing value. log2(f∆) was calculated between the ATP+ and ATP- conditions. (A) All RNA, (B) lncRNA, (C) mRNA. Colors designate whether an Sm-site is **Absent** (yellow), **Noncanonical** (green), **U7** (blue), or **U1**, **U2**, or **U5** (purple, darkest to lightest). Values above CDFs indicate the number of genes plotted for each condition in color. Wilcoxon Rank Sum Tests with Continuity Correction and Benjamini-Hochberg False Discovery Rate corrections for multiple testing were used to calculate adjusted *p-values* for the left color being greater than the right color are provided in the upper lefthand corner of the graphs.

**Supplementary Figure 13. The frequency of Sm-sites in an RNA correlates with further Sm-protein
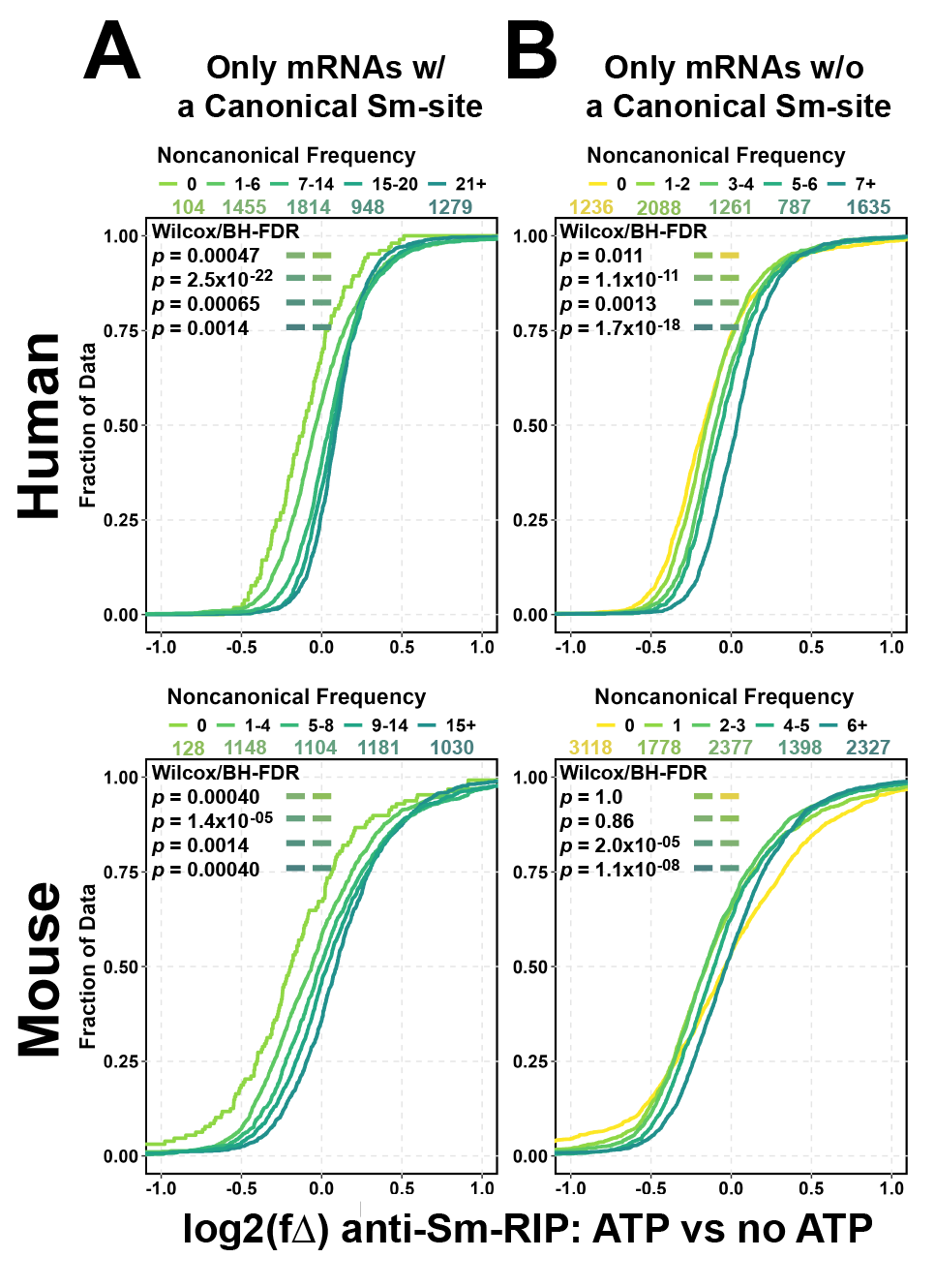
 enrichment upon addition of ATP into the assembly reactions.** **(top)** for human analysis and **(bottom)** for mouse analysis. For each cumulative distribution plot, log2 fold change (log2(f∆)) between the anti-Sm-RIP supplemented with polyA-RNA and ATP vs without ATP conditions are plotted in increasing order, delineated by the frequency of Sm-sites predicted for the gene product. Values above CDFs indicate the number of genes plotted for each condition in color. Wilcoxon Rank Sum Tests with Continuity Correction and Benjamini-Hochberg False Discovery Rate corrections for multiple testing were used to calculate adjusted *p-values* for the left color being greater than the right color are provided in the upper lefthand corner of the graphs. **(A)** Cumulative distribution plot of only mRNAs predicted to contain at least one canonical Sm-site, delineating the frequency of noncanonical Sm-sites predicted within the mRNA transcript, against the log2 fold change in anti-Sm-RIP supplemented with polyA-RNA and ATP vs anti-Sm-RIPs supplemented only with polyA-RNA. **(B)** Cumulative distribution plot of mRNAs predicted to solely contain noncanonical Sm-sites, delineating the frequency of noncanonical Sm-sites predicted within the mRNA transcript, against the log2 fold change in anti-Sm-RIP supplemented with polyA-RNA and ATP vs anti-Sm-RIPs supplemented only with polyA-RNA.


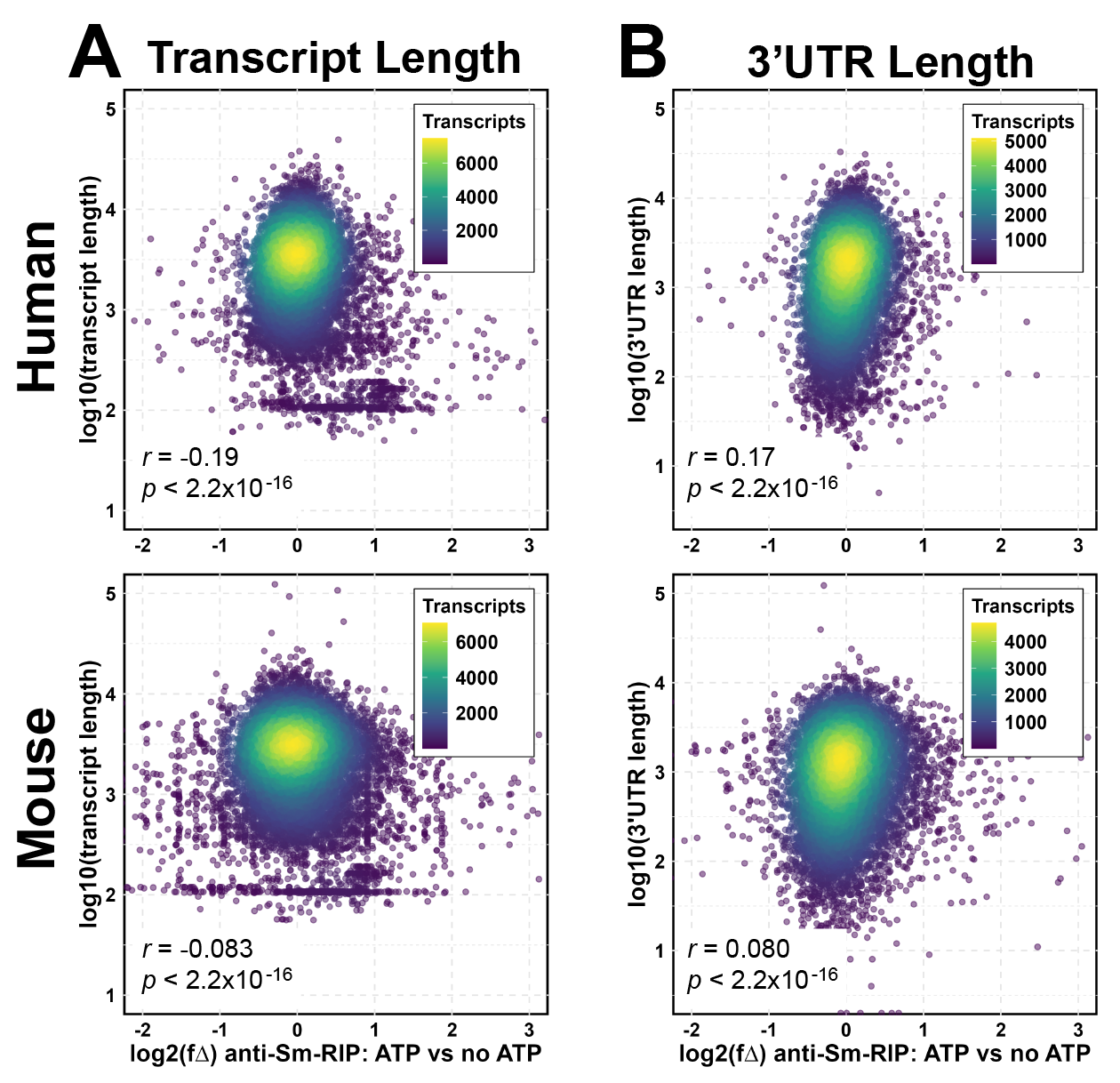


**Supplementary Figure 14: Transcript or 3’UTR length is not a predictor of ATP-dependent enrichment in anti-Sm-RIP experiments.** **(top)** for human analysis and **(bottom)** for mouse analysis. **(A)** log10 of transcript length plotted against log2 fold change between anti-Sm-RIP supplemented with polyA-RNA and ATP vs anti-Sm-RIPs supplemented only with polyA-RNA. *r* is a Pearson correlation coefficient. **(B)** log10 of 3’UTR length plotted against log2 fold change between anti-Sm-RIP supplemented with polyA-RNA and ATP vs anti-Sm-RIPs supplemented only with polyA-RNA. *r* is a Pearson correlation coefficient.

**Supplementary Figure 15: U
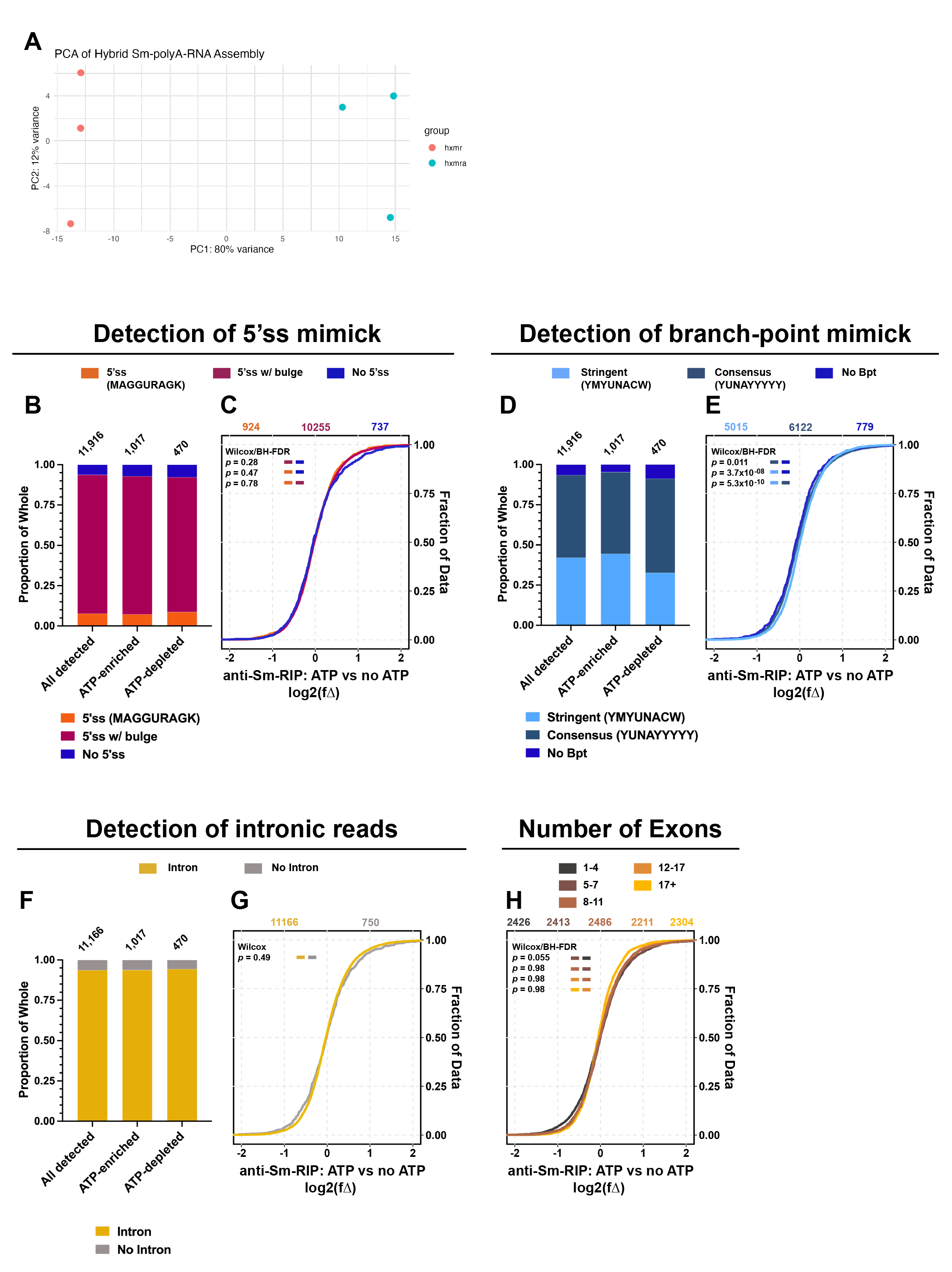
 snRNP-binding does not predict enrichment with Sm-proteins in an ATP-dependent manner.** RIPs following incubation of human cytoplasmic extract with mouse polyA-RNA in the presence or absence of ATP. Reactions were treated for 15 min with 2 M urea and 5 mg/mL heparin, immunoprecipitation was performed in 2 mg/mL heparin and RSB-1000 + 0.1% NP-40, and washed 8 times with RSB-1000 + 0.1% NP-40. U7 Sm-site-containing mRNAs were removed from analysis as this site is both infrequent and receives a specialized Sm-ring differentiating it from U1, U2, and U5 type Sm-sites. **(A)** Principal Component Analysis for sequenced samples. Human cytoplasmic extract incubated with mouse polyA-RNA in absence of ATP (**hxmr**, red) and in the presence of ATP (**hxmra**, green). For **B-E**, colors indicate whether the gene encodes a transcript containing a mimic of U snRNA complementary sequence: 5’ splice-site (**5’ss**, orange), a 5’ss allowing for a single mismatch or deletion (**5’ss w/ bulge**, maroon), a Stringent U2/U12 complement (**Stringent**, sky blue), a Consensus branch-point (**Consensus**, blue grey), or that a 5’ss or branch-point is absent (**No 5’ss**/**Bpt**, royal blue). For **F-G**, colors indicate whether a single read has been counted within an intron of the gene product for the anti-Sm-RIP condition: **Intron** (gold) and **No Intron** (grey). For **BDF**, proportional bar graph of mRNAs enriched (log2(f∆) > 0.6, *padj* < 0.05) in anti-Sm-RIP: ATP vs no ATP condition, stratified by detected Sm-sites. **All** indicates the total different transcripts mapped in the sequenced library, **Sm-enriched** are those transcripts enriched (log2(f∆) > 0.6 *padj* < 0.05) in the anti-Sm-RIP. **Sm-depleted** are those transcripts that were significantly depleted (log2(f∆) < -0.6, *padj* < 0.05) in the anti-Sm-RIP. For **CEG**, cumulative distribution plots for mRNAs, plotting the log2 fold change (log2(f∆)) by increasing value, stratified by whether the RNA contains an Sm-site, sequence complementary to U snRNAs, or a read has been assigned within an intron. log2(f∆) was calculated between the anti-Sm-RIP(Y12): ATP vs no ATP conditions. **(H)**  CDF for mRNAs stratified by binned number of exons within the transcript. Colors indicate increasing number of exons per transcript. Values above CDFs indicate the number of genes plotted for each condition in color. Wilcoxon Rank Sum Tests with Continuity Correction and Benjamini-Hochberg False Discovery Rate corrections for multiple testing were used to calculate adjusted *p-values* for the left color being greater than the right color are provided in the upper lefthand corner of the graphs.


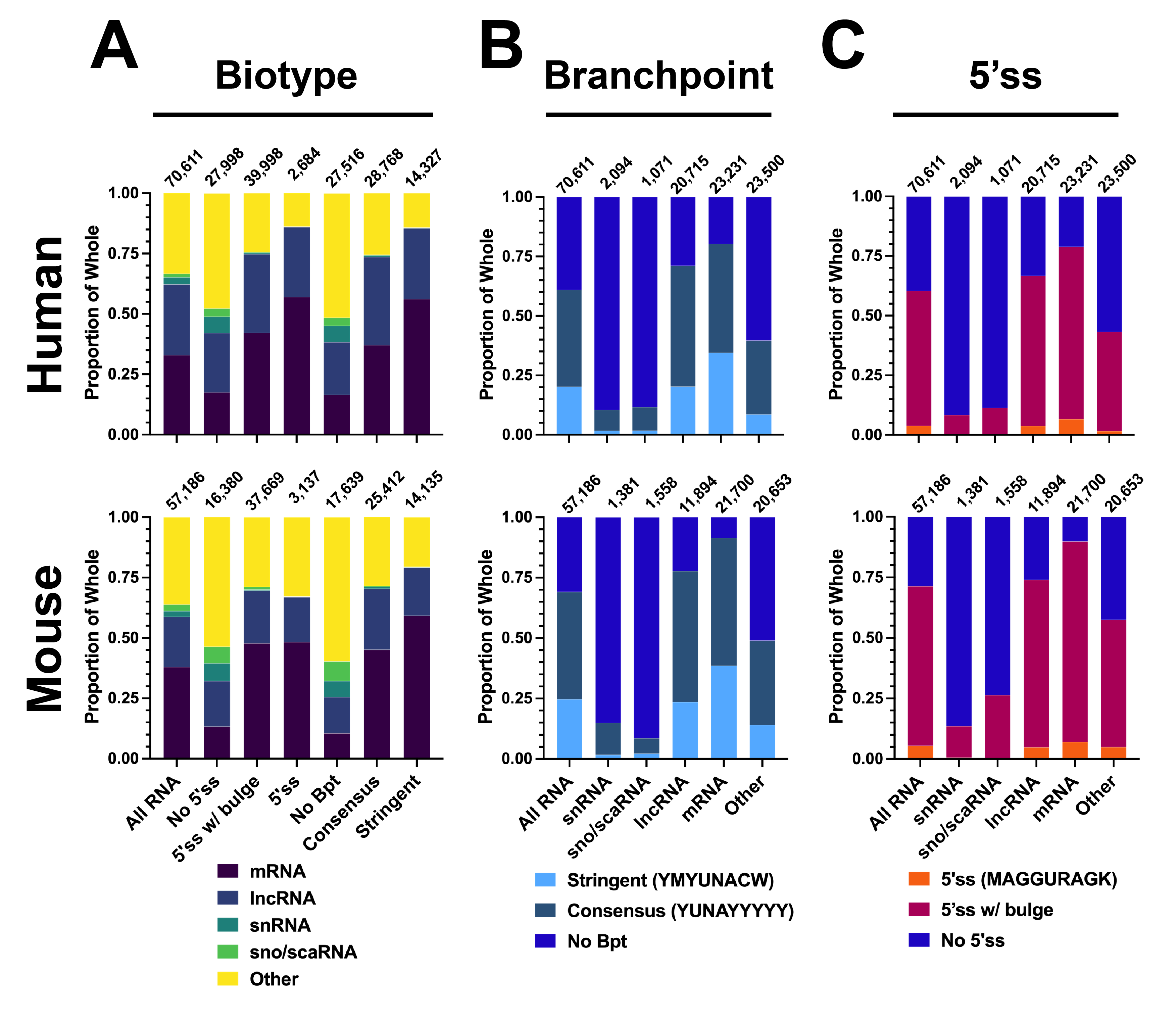
**Supplementary Figure 16: Breakdown of sequences mimicking 5’ splice-sites and branch-points detected in NCBI Refseq and Gencode human and mouse transcriptomes.** **(top)** for human analysis and **(bottom)** for mouse analysis. For all bar graphs, top numbers are the number of genes represented within the bar. Data presented in **A**-**C** correspond to a single, unique transcript ID of a single, unique gene. **(A)** Proportional bar graph of RNA biotypes. **All** is a breakdown of the annotated genome. **No 5’ss** are RNAs not predicted to contain a 5’ splice-site mimicking sequence. **5’ss w/ bulge** are those RNAs predicted to contain the consensus 5’ss sequence (MAGGURAGK) allowing for a single mismatch or deletion. **5’ss** are those RNAs predicted to contain the consensus 5’ss sequence (MAGGURAGK). **No Bpt** are RNAs not predicted to contain a branch-point mimicking sequence. **Consensus** are those RNAs only predicted to contain the consensus YUNAYYYYY mammalian branch-point sequence. **Stringent** are RNAs predicted to contain the Stringent U2/U12-specific YMYUNACW complementary sequence. **(B)** Proportional bar graph giving a breakdown of types of 5’ splice-sites sequences predicted in each of the following biotypes: All, snRNA, sno/scaRNA, lncRNA, mRNA, and Other. **(C)** Proportional bar graph giving a breakdown of types of branch-point sequences predicted in each of the following biotypes: All, snRNA, sno/scaRNA, lncRNA, mRNA, and Other.


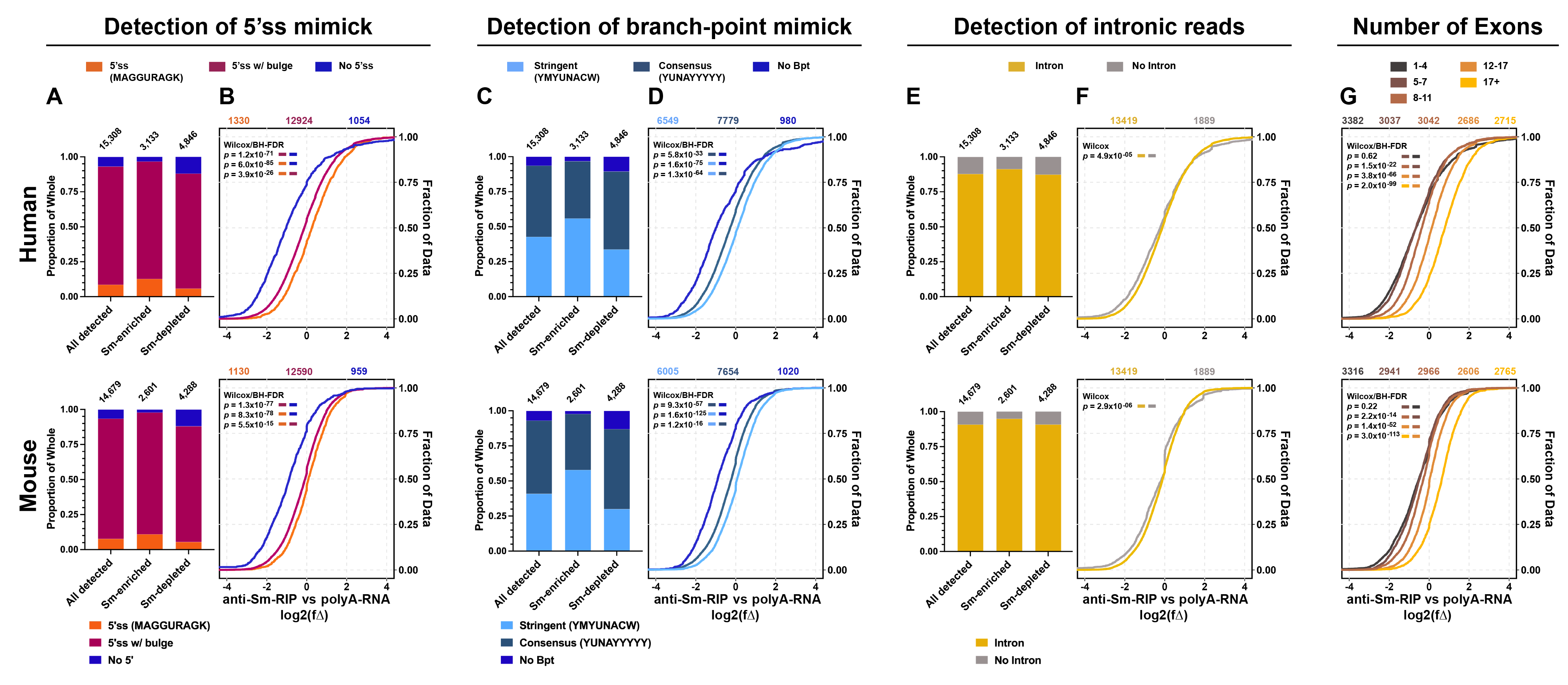


**Supplementary Figure 17: mRNAs containing sequences complementary to snRNAs are enriched with Sm-proteins.** For **A-F**, **(top)** human analysis and **(bottom)** mouse analysis. U7 Sm-site-containing mRNAs were removed from analysis as this site is both infrequent and receives a specialized Sm-ring differentiating it from U1, U2, and U5 type Sm-sites. For **A-D**, colors indicate whether the gene encodes a transcript containing a mimic of U snRNA complementary sequence: 5’ splice-site (**5’ss**, orange), a 5’ss allowing for a single mismatch or deletion (**5’ss w/ bulge**, maroon), a Stringent U2/U12 complement (**Stringent**, sky blue), a Consensus branch-point (**Consensus**, blue grey), or that a 5’ss or branch-point is absent (**No 5’ss**/**No** **Bpt**, royal blue). For **E-F**, colors indicate whether a single read has been counted within an intron of the gene product for the anti-Sm-RIP condition: **Intron** (gold) and **No Intron** (grey). For **ACE,** proportional bar graph of mRNAs enriched (log2(f∆) > 0.6, *padj* < 0.05) in anti-S-RIP vs the polyA-RNA transcriptome, stratified by the presence of a U snRNA complementary sequence. **All** indicates the total different transcripts mapped in the sequenced library, **Sm-enriched** are those transcripts enriched (log2(f∆) > 0.6 *padj* < 0.05) in the anti-Sm-RIP. **Sm-depleted** are those transcripts that were significantly depleted (log2(f∆) < -0.6, *padj* < 0.05) in the anti-Sm-RIP. For **BDF**, cumulative distribution plots for mRNAs stratified by U snRNA complementary sequence detected, plotting the log2 fold change (log2(f∆)) by increasing value. log2(f∆) was calculated between the anti-Sm-RIP vs polyA-RNA conditions. **(B)** 5’ss, **(D)** Bpt, **(F)** Intron. **(G)** CDF for mRNAs stratified by binned number of exons within the transcript, plotting the log2 fold change (log2(f∆)) by increasing value. log2(f∆) was calculated between the anti-Sm-RIP vs polyA-RNA conditions. Colors indicate increasing number of exons per transcript. Values above CDFs indicate the number of genes plotted for each condition in color. Wilcoxon Rank Sum Tests with Continuity Correction and Benjamini-Hochberg False Discovery Rate corrections for multiple testing were used to calculate adjusted *p-values* for the left color being greater than the right color are provided in the upper lefthand corner of the graphs.


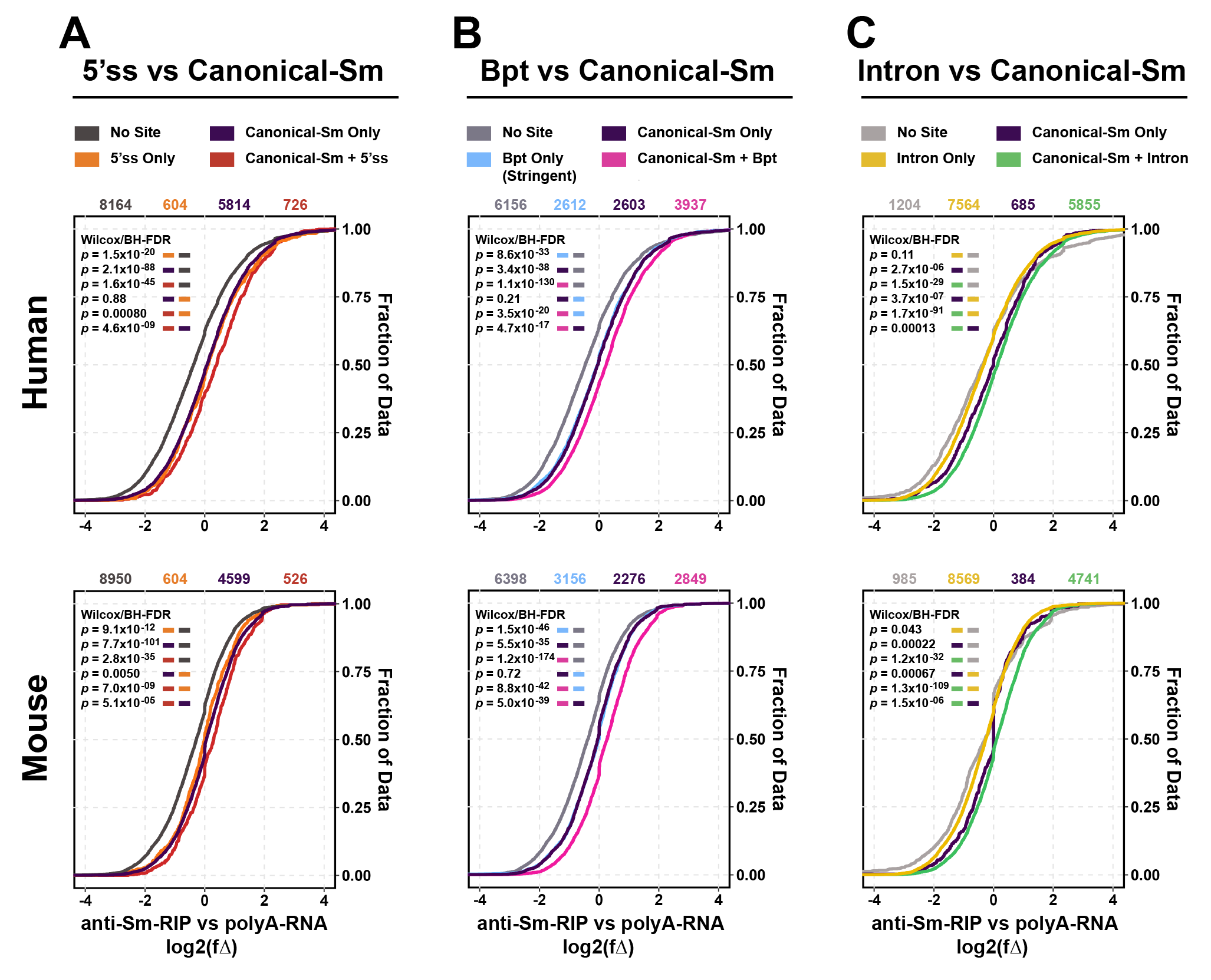


**Supplementary Figure 18: mRNAs predicted to contain a canonical Sm-site and complementary sequence to U snRNAs are most enriched in anti-Sm-RIPs.** Cumulative distribution plots for mRNAs, stratified by U snRNA complementary sequence and canonical Sm-site, plotting the log2 fold change (log2(f∆)) by increasing value. log2(f∆) was calculated between the anti-Sm-RIP vs polyA-RNA conditions. **(A)** for mimic of 5’ splice sites: **No Site** (dark grey), only a 5’ splice-site mimic (**5’ss Only**, orange), only a Canonical Sm-site (**Canonical-Sm Only**, purple), and the presence of both a canonical Sm-site and 5’ss (**Canonical-Sm + 5’ss**, red). **(B)** for mimic of branch-point sequence: **No Site** (dark grey), only a stringent branch-point mimic (**Bpt Only**, sky blue), only a Canonical Sm-site (**Canonical-Sm Only**, purple), and the presence of both a canonical Sm-site and stringent branch-point mimic (**Canonical-Sm + Bpt**, pink). **(C)** for detection of a read in an intron: **No Site** (grey), detection of an intronic read only (**Intron Only**, gold), only a Canonical Sm-site (**Canonical-Sm Only**, purple), and the presence of both a canonical Sm-site and an intronic read (**Canonical-Sm + Intron**, green). Values above CDFs indicate the number of genes plotted for each condition in color. Wilcoxon Rank Sum Tests with Continuity Correction and Benjamini-Hochberg False Discovery Rate corrections for multiple testing were used to calculate adjusted *p-values* for the left color being greater than the right color are provided in the upper lefthand corner of the graphs.


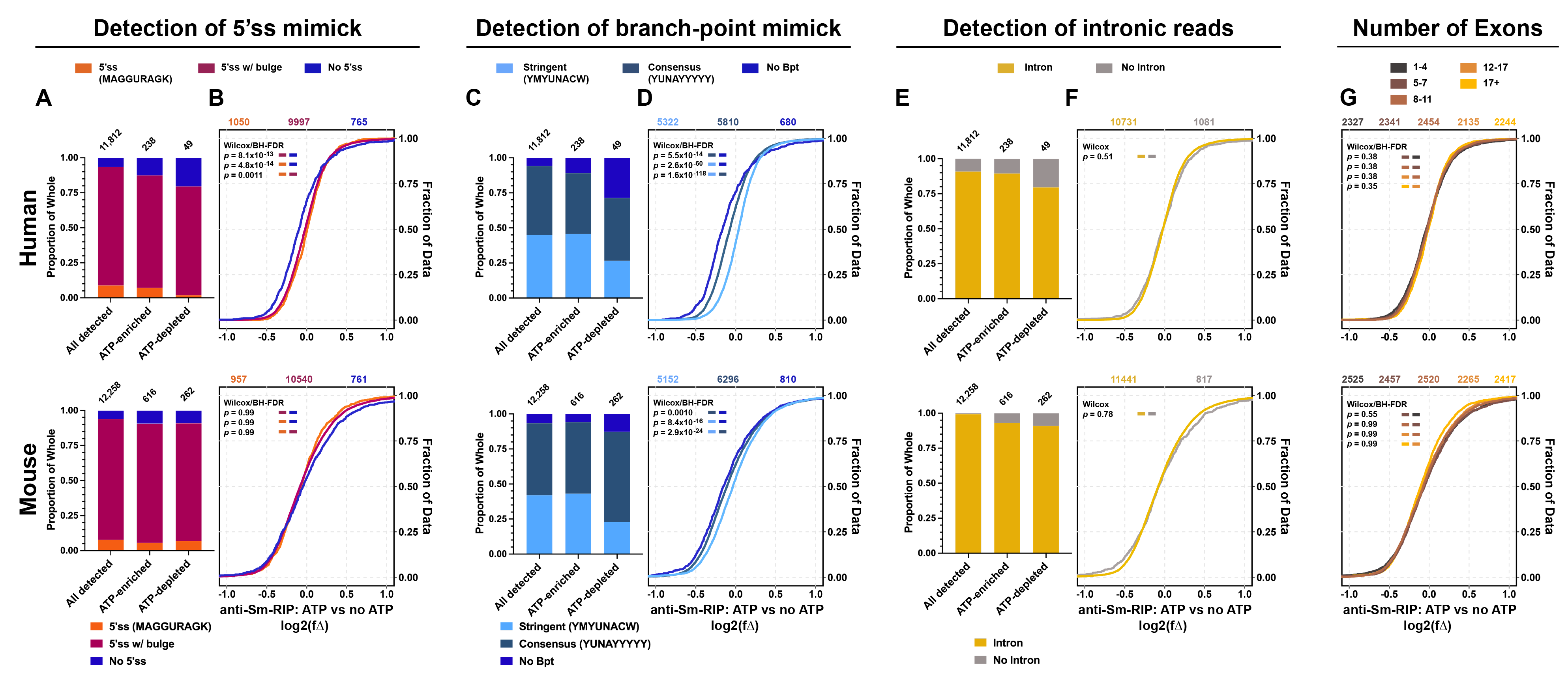


**Supplementary Figure 19: Canonical Sm-site mRNAs are specifically enriched in anti-Sm-RIPs in an ATP-dependent and U snRNA independent manner.** For **A-F**, **(top)** human analysis and **(bottom)** mouse analysis. U7 Sm-site-containing RNAs were removed from analysis as this site is both infrequent and receives a specialized Sm-ring differentiating it from U1, U2, and U5 type Sm-sites. For **A-D**, colors indicate whether the gene encodes a transcript containing a mimic of U snRNA complementary sequence: 5’ splice-site (**5’ss**, orange), a 5’ss allowing for a single mismatch or deletion (**5’ss w/ bulge**, maroon), a Stringent U2/U12 complement (**Stringent**, sky blue), a Consensus branch-point (**Consensus**, blue grey), or that a 5’ss or branch-point is absent (**No 5’ss**/**No Bpt**, royal blue). For **E-F**, colors indicate whether a single read has been counted within an intron of the gene product for the polyA-RNA condition: **Intron** (gold) and **No Intron** (grey). For **ACE**, proportional bar graph of mRNAs enriched (log2(f∆) > 0.6, *padj* < 0.05) in anti-Sm-RIP supplemented with ATP vs anti-Sm-RIP not supplemented with ATP, stratified by the presence of a U snRNA complementary sequence. Proportional bar graph giving a breakdown of types of Sm-sites in mRNAs represented in the analysis (**All**), represented in those mRNAs enriched following anti-Sm-RIP (**ATP-enriched**, log2(f∆) > 0.6, *padj* < 0.05), and those not-enriched (**ATP-depleted**, log2(f∆) < -0.6, *padj* < 0.05). For BDF, cumulative distribution plots for mRNAs stratified by U snRNA complementary sequence detected, plotting the log2 fold change (log2(f∆)) by increasing value. log2(f∆) was calculated between the anti-Sm-RIP vs polyA-RNA conditions. **(B) 5’ss**, **(D) Bpt**, **(F) Intron**. **(G)** CDF for mRNAs stratified by binned number of exons within the transcript, plotting the log2 fold change (log2(f∆)) by increasing value. log2(f∆) was calculated between the anti-Sm-RIP vs polyA-RNA conditions. Colors indicate increasing number of exons per transcript. Values above CDFs indicate the number of genes plotted for each condition in color. Wilcoxon Rank Sum Tests with Continuity Correction and Benjamini-Hochberg False Discovery Rate corrections for multiple testing were used to calculate adjusted *p-values* for the left color being greater than the right color are provided in the upper lefthand corner of the graphs.


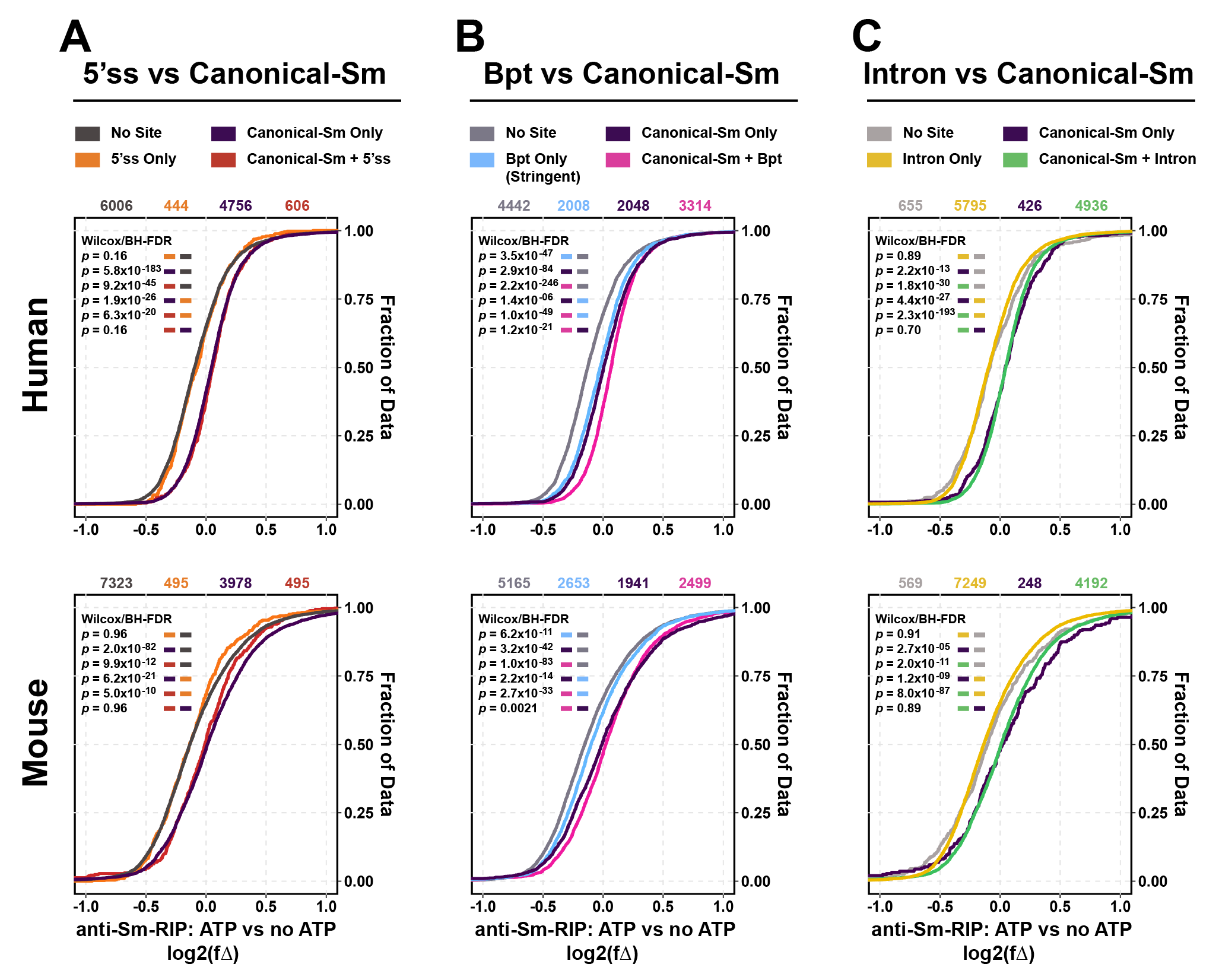


**Supplementary Figure 20: Prediction of a canonical Sm-site in mRNAs is deterministic over U snRNA complementarity for ATP-dependent Sm-association.** Cumulative distribution plots for mRNAs, stratified by U snRNA complementary sequence and canonical Sm-site, plotting the log2 fold change (log2(f∆)) by increasing value. log2(f∆) was calculated between anti-Sm-RIP supplemented with ATP vs anti-Sm-RIP not supplemented with ATP. **(A)** for mimic of 5’ splice sites: **No Site** (dark grey), only a 5’ splice-site mimic (**5’ss Only**, orange), only a Canonical Sm-site (**Canonical-Sm Only**, purple), and the presence of both a canonical Sm-site and 5’ss (**Canonical-Sm + 5’ss**, red). **(B)** for mimic of branch-point sequence: **No Site** (dark grey), only a stringent branch-point mimic (**Bpt Only**, sky blue), only a Canonical Sm-site (**Canonical-Sm Only**, purple), and the presence of both a canonical Sm-site and stringent branch-point mimic (**Canonical-Sm + Bpt**, pink). **(C)** for detection of a read in an intron: **No Site** (grey), detection of an intronic read only (**Intron Only**, gold), only a Canonical Sm-site (**Canonical-Sm Only**, purple), and the presence of both a canonical Sm-site and an intronic read (**Canonical-Sm + Intron**, green). Values above CDFs indicate the number of genes plotted for each condition in color. Wilcoxon Rank Sum Tests with Continuity Correction and Benjamini-Hochberg False Discovery Rate corrections for multiple testing were used to calculate adjusted *p-values* for the left color being greater than the right color are provided in the upper lefthand corner of the graphs.


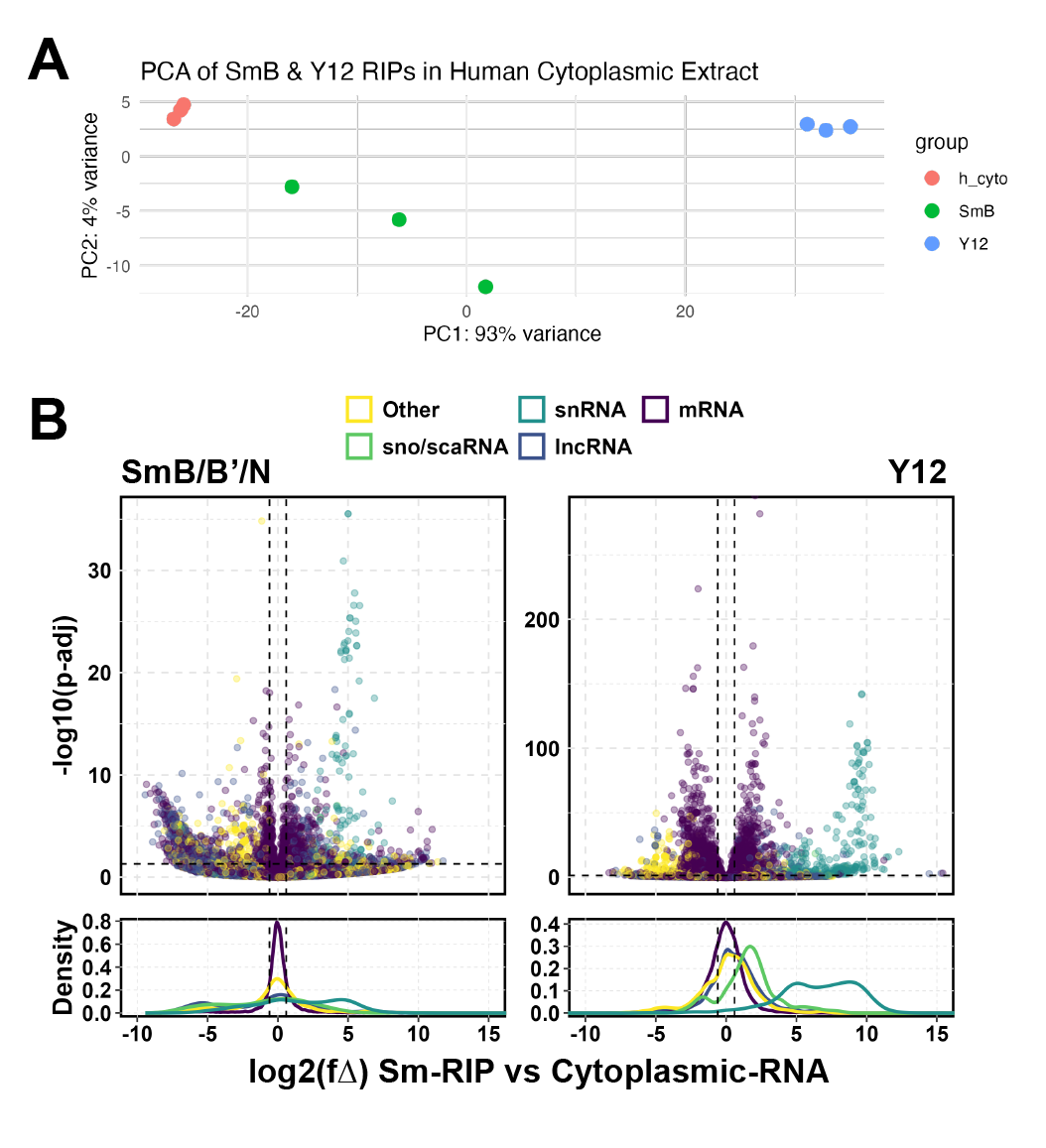


**Supplementary Figure 21: RNAs captured by Y12 and SmB/B’/N-specific antibodies are similar.** **(A)** Principal component analysis for each sequencing library color coded by the sample condition. For human cytoplasmic RNA (**h_cyto**, red), SmB/B’/N-RIP (**SmB**, green), and Y12-RIP (**Y12**, blue). **(B)** Volcano plot and accompanying density plot indicating the different types of RNAs enriched in SmB/B’/N **(left)** of Y12 **(right)** RNA immunoprecipitation when compared to the human cytoplasmic transcriptome. X-axis for volcano and density plots are shared—log2 fold change (log2(f∆)) of anti-Sm-RIP vs human cytoplasmic transcriptome.


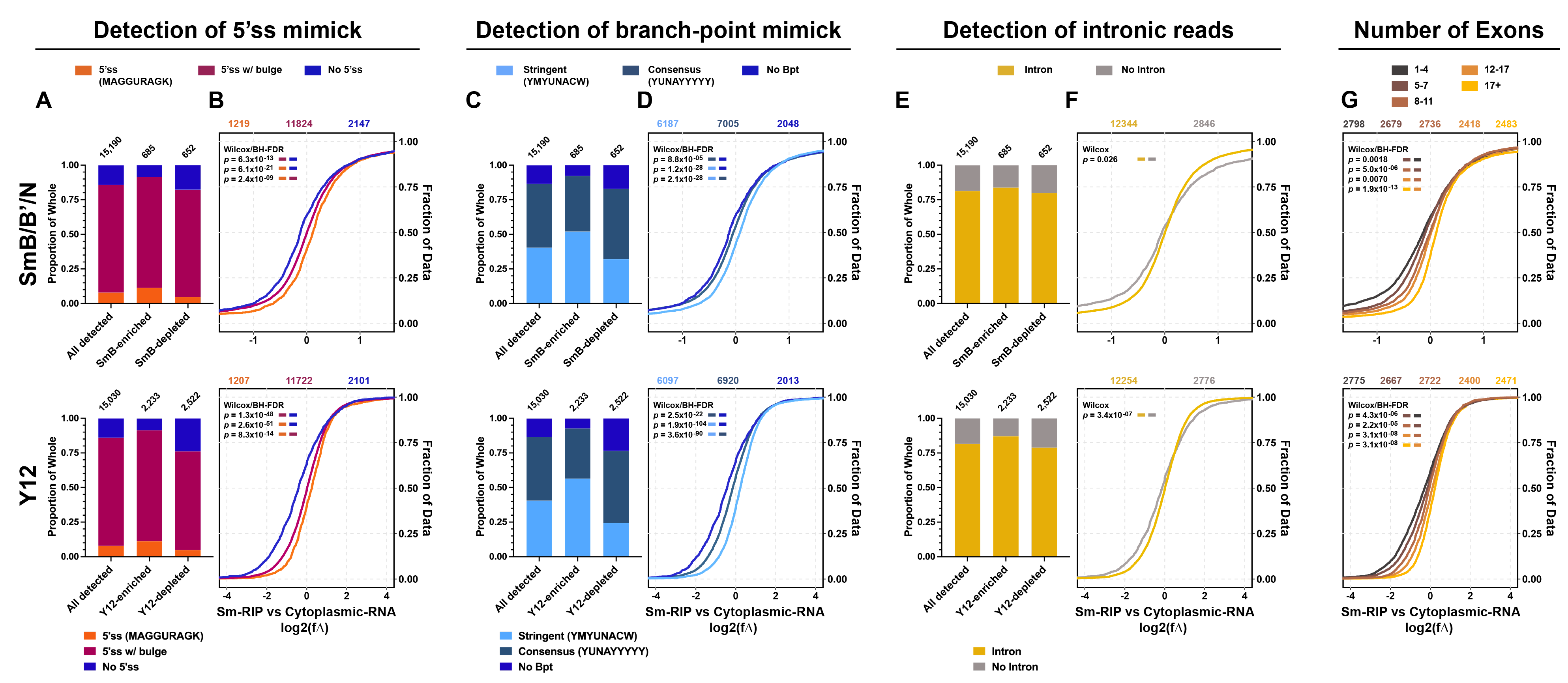
**Supplementary Figure 22: Y12 and SmB/B’/N antibodies specifically capture human mRNAs containing sequences complementary to snRNAs.** For **A-F**, **(top)** SmB/B’/N analysis and **(bottom)** Y12 analysis. U7 Sm-site-containing mRNAs were removed from analysis as this site is both infrequent and receives a specialized Sm-ring differentiating it from U1, U2, and U5 type Sm-sites. For **A-D**, colors indicate whether the gene encodes a transcript containing a mimic of U snRNA complementary sequence: 5’ splice-site (**5’ss**, orange), a 5’ss allowing for a single mismatch or deletion (**5’ss w/ bulge**, maroon), a Stringent U2/U12 complement (**Stringent**, sky blue), a Consensus branch-point (**Consensus**, blue grey), or that a 5’ss or branch-point is absent (**No 5’ss**/**No** **Bpt**, royal blue). For **E-F**, colors indicate whether a single read has been counted within an intron of the gene product for the anti-Sm-RIP condition: **Intron** (gold) and **No Intron** (grey). For **ACE,** proportional bar graph of mRNAs enriched (log2(f∆) > 0.6, *padj* < 0.05) in anti-Sm-RIP vs the human cytoplasmic transcriptome, stratified by the presence of a U snRNA complementary sequence. **All** indicates the total different transcripts mapped in the sequenced library, **Sm-enriched** are those transcripts enriched (log2(f∆) > 0.6 *padj* < 0.05) in the anti-Sm-RIP. **Sm-depleted** are those transcripts that were significantly depleted (log2(f∆) < -0.6, *padj* < 0.05) in the anti-Sm-RIP. For **BDF**, cumulative distribution plots for mRNAs stratified by U snRNA complementary sequence detected, plotting the log2 fold change (log2(f∆)) by increasing value. log2(f∆) was calculated between the anti-Sm-RIP vs human cytoplasmic RNA conditions. **(B)** 5’ss, **(D)** Bpt, **(F)** Intron. **(G)** CDF for mRNAs stratified by binned number of exons within the transcript, plotting the log2 fold change (log2(f∆)) by increasing value. log2(f∆) was calculated between the anti-Sm-RIP vs human cytoplasmic RNA conditions. Colors indicate increasing number of exons per transcript. Values above CDFs indicate the number of genes plotted for each condition in color. Values above CDFs indicate the number of genes plotted for each condition in color. Wilcoxon Rank Sum Tests with Continuity Correction and Benjamini-Hochberg False Discovery Rate corrections for multiple testing were used to calculate adjusted *p-values* for the left color being greater than the right color are provided in the upper lefthand corner of the graphs.

**
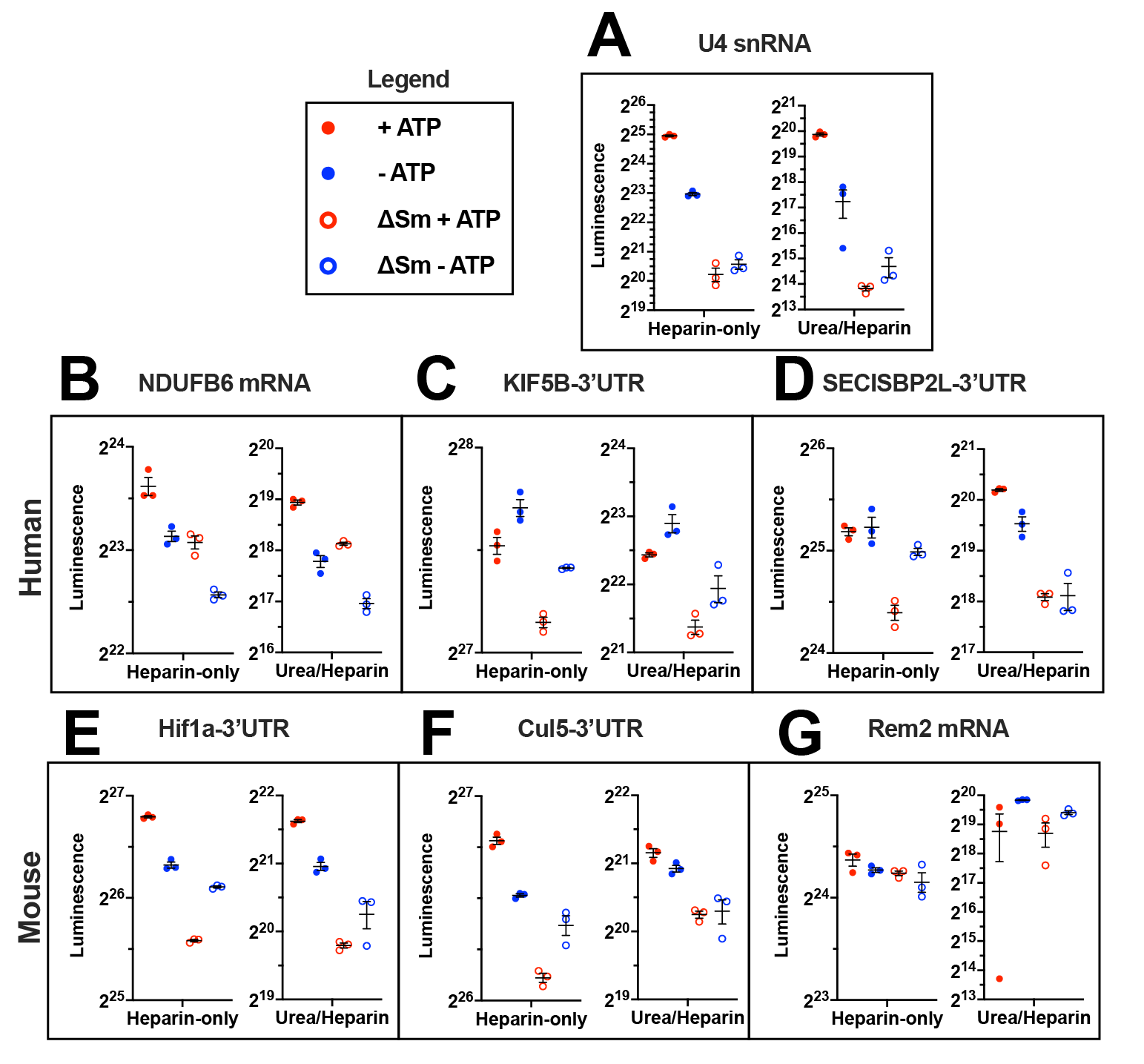
Supplementary Figure 23: Raw luminescence values for ATP and Sm-site dependent assembly of Sm-protein rings on mRNAs as shown in Figure 4D-J**. **(A-G)** Luminescence results from detection of *in vitro* transcribed, biotin-labelled human U4 snRNA **(A)**, mRNAs (NDUFB6 **(B)** and Rem2 **(G)**), and mRNA 3’UTRs (KIF5B **(C)**, SECISBP2L **(D)**, Hif1a **(E)**, and Cul5 **(F)**) enriched following anti-Sm-RIP. 4 conditions were performed for each RNA: (solid red dot) cytoplasmic cell extract supplemented with wild-type RNA and ATP, (solid blue dot) cytoplasmic cell extract supplemented with wild-type RNA but not with ATP, (open red circle) cytoplasmic cell extract supplemented with ATP and RNA mutated to remove the Sm-site sequence, and (open blue circle) cytoplasmic cell extract supplemented with RNA mutated to remove the Sm-site sequence but not ATP. All shown RNAs have a canonical Sm-site except Rem2. Left graphs are the results obtained by performing the immunoprecipitation in 2 mg/mL heparin, RSB-500 + 0.1% NP-40 and washed 8 times with RSB-500 + 0.1% NP-40. Right graphs are the results obtained following 15 min treatment with 2M urea and 5 mg/mL heparin, followed by immunoprecipitation in 2 mg/mL heparin, RSB-500 + 0.1% NP-40 and washed 8 times with RSB-500 + 0.1% NP-40 and are also shown in presented in Figure 6D-H. Raw luminescence values are comparable between Heparin-only and Urea/Heparin conditions were performed on separate plates. Raw luminescence is comparable for the different RNAs within these groups as they were performed at the same time, using the same reagents, and same plate.

**Supplementary Figure 24: Sm-ring assembly on mRNAs is reproducible with an antibody specific for
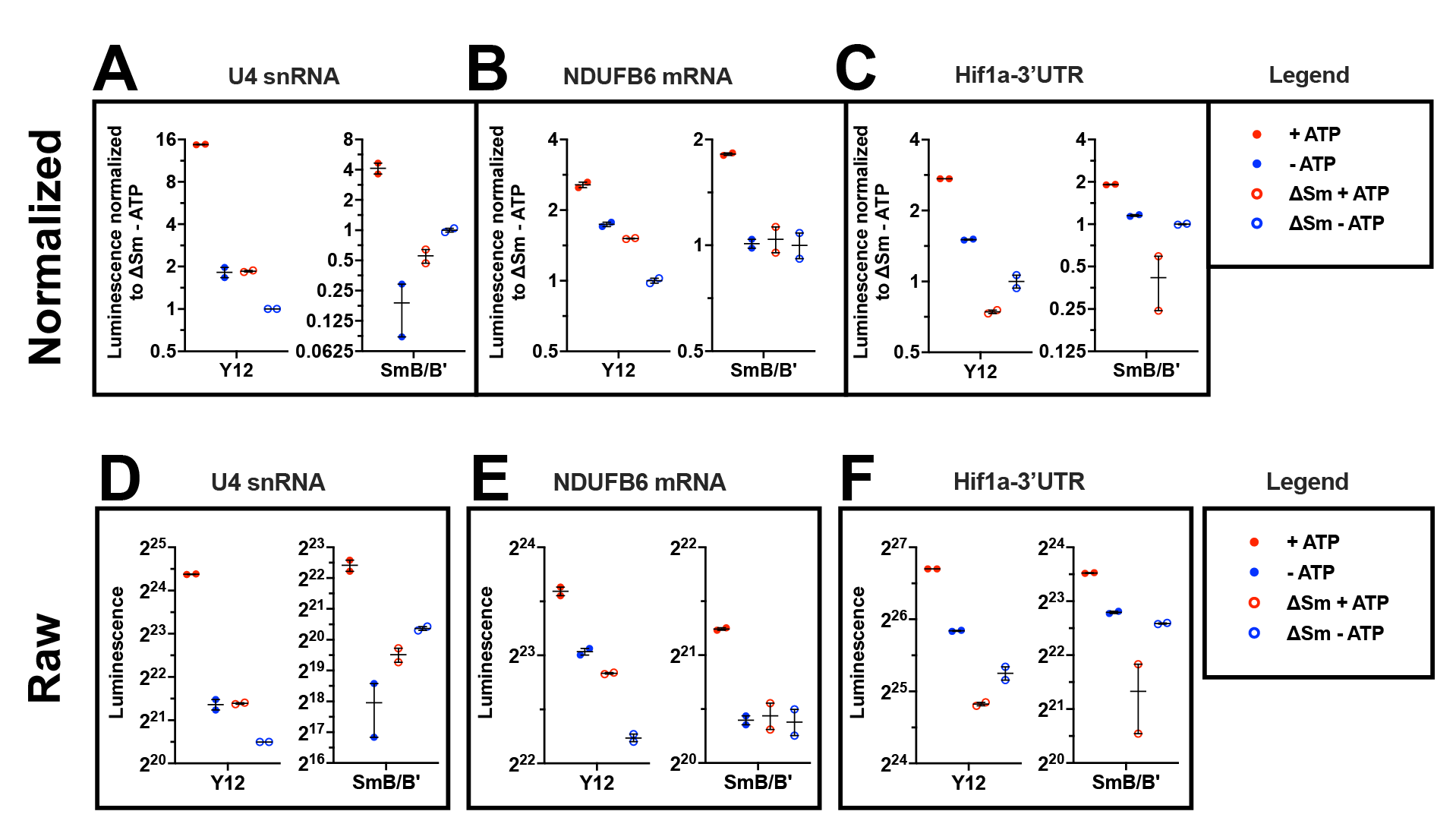
 SmB/B’.** Luminescence results from detection of *in vitro* transcribed, biotin-labelled human U4 snRNA **(AD)**, NDUFB6 mRNA **(BE)** and Hif1a 3’UTR **(CF)** enriched following anti-Sm-RIP. 4 conditions were performed for each RNA: (solid red dot) cytoplasmic cell extract supplemented with wild-type RNA and ATP, (solid blue dot) cytoplasmic cell extract supplemented with wild-type RNA but not with ATP, (open red circle) cytoplasmic cell extract supplemented with ATP and RNA mutated to remove the Sm-site sequence, and (open blue circle) cytoplasmic cell extract supplemented with RNA mutated to remove the Sm-site sequence but not ATP. All shown RNAs have a canonical Sm-site and all reactions were incubated with 2M urea and 5 mg/mL heparin for 15 min, followed by immunoprecipitation in 2 mg/mL heparin, RSB-500 + 0.1% NP-40 and washed 8 times with RSB-500 + 0.1% NP-40. Left graphs are the results obtained by performing the immunoprecipitation with the infamous Y12 antibody, and right was performed using an SmB/B’ specific antibody. Raw luminescence values are given on the bottom and luminescence normalized to the ∆Sm-ATP condition are given on the top set of graphs.

**Supplementary Figure 25: Principal component analyses for external sequencing data used in this
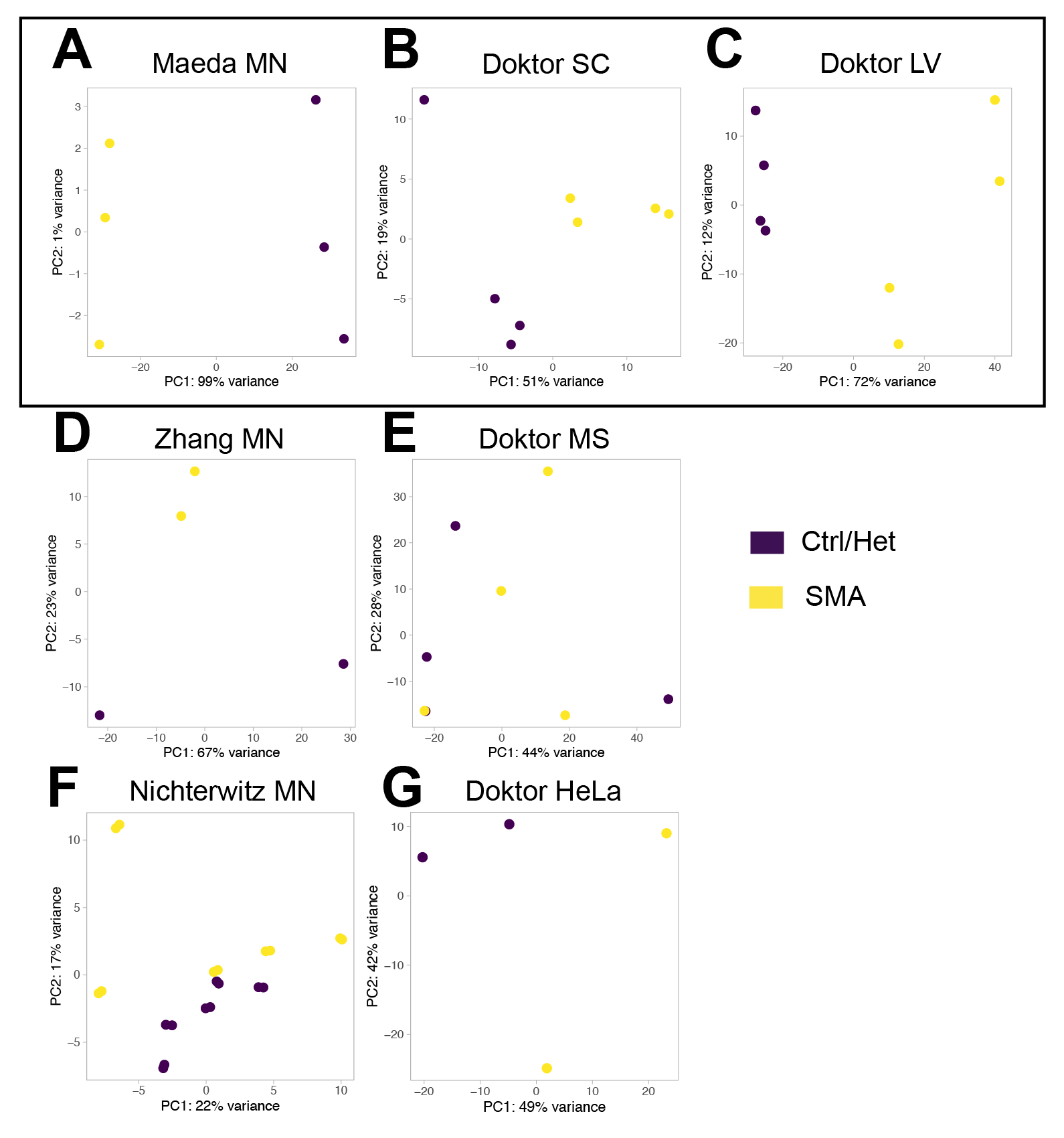
 study.** Boxed PCA plots **(A-C)** indicate those datasets used for analyses described in Figure 5. Un-boxed PCA plots **(D-G)** were excluded from analysis as the principal variance between samples is not due to the sample genotype and therefore any variations cannot be expected to be due to a decrease in SMN. **(A)** comparison of mESC differentiated motor neurons derived from SMA (*Smn^+/-^*;*SMN2^+/+^*) and normal (*Smn^+/+^*;*SMN2^+/+^*) as published by Maeda *et al PLoS ONE* 2014 (96). **(B)** comparison of spinal cord lysates collected from post-natal day 5 Taiwanese SMA (*Smn^-/-^*;*SMN2^+/+^*) and Taiwanese Het (*Smn^+/-^*;*SMN2^+/+^*) mice as published by Doktor *et al NAR* 2017 (97). **(C)** comparison of liver lysates collected from post-natal day 5 Taiwanese SMA (*Smn^-/-^*;*SMN2^+/+^*) and Taiwanese Het (*Smn^+/-^*;*SMN2^+/+^*) mice as published by Doktor *et al NAR* 2017 (97). **(D)** comparison of post-natal-day 1 ∆7SMA (*Smn^-/-^*;*SMN2^+/+^*;SMN∆7^+/+^) and ∆7Het (*Smn^+/-^*;*SMN2^+/+^*;SMN∆7^+/+^) laser-microdissected mouse motor neurons as published by Zhang *et al PNAS* 2014 (115). **(E)** comparison of muscle lysates collected from post-natal day 5 Taiwanese SMA (*Smn^-/-^*;*SMN2^+/+^*) and Taiwanese Het (*Smn^+/-^*;*SMN2^+/+^*) mice as published by Doktor *et al NAR* 2017 (97). **(F)** comparison of post-natal-day 5 ∆7SMA (*Smn^-/-^*;*SMN2^+/+^*;SMN∆7^+/+^) and ∆7Het (*Smn^+/-^*;*SMN2^+/+^*;SMN∆7^+/+^) laser-microdissected mouse motor neurons as published by Nichterwitz *et al* (98). **(G)** comparison of siRNA-mediated knockdown of SMN in HeLa cell extracts as published by Doktor *et al NAR* 2017 (97).

**Supplementary Figure 26. Sm-site containing RNAs are associated with intracellular vesicle
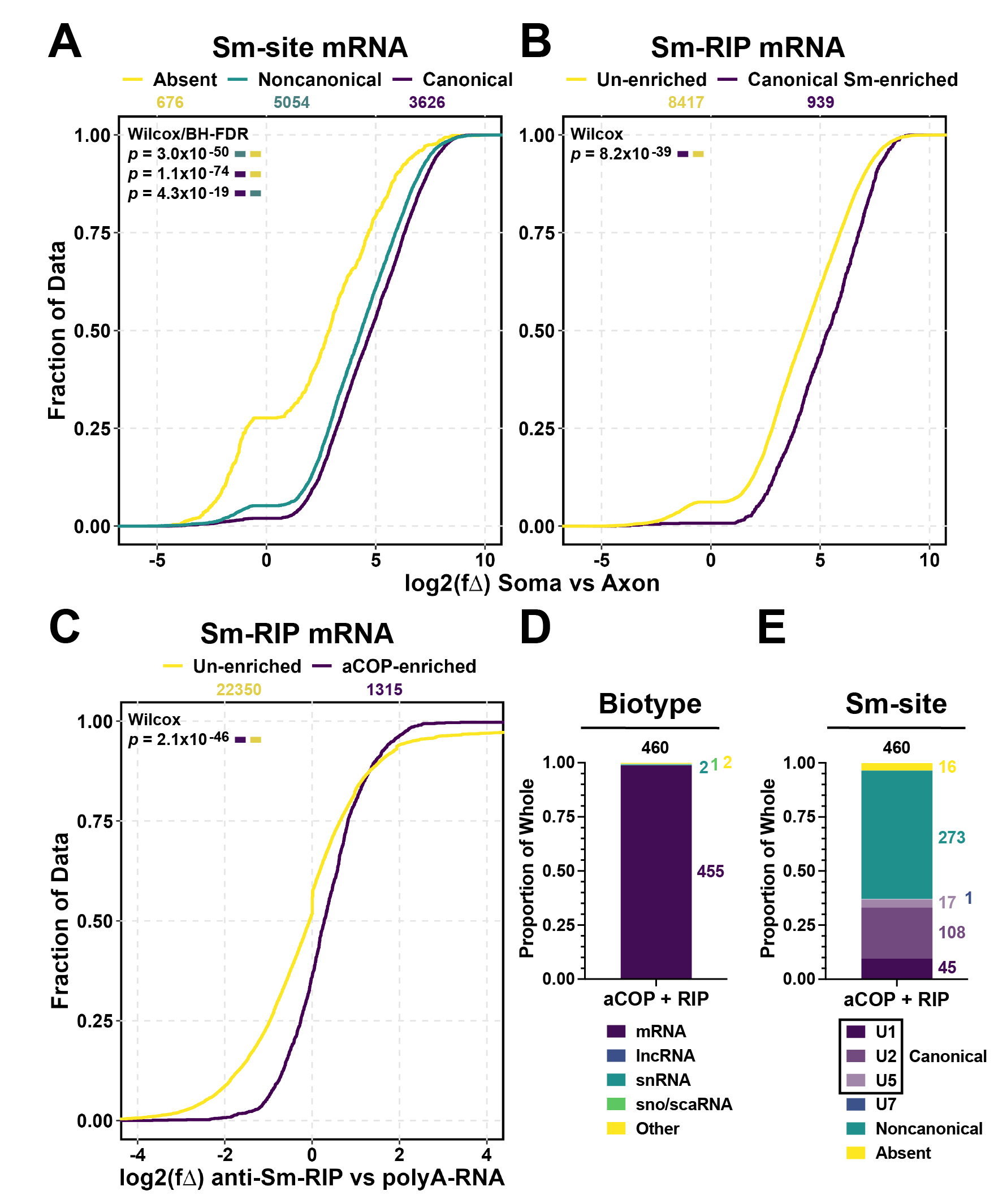
 trafficking machinery.** For **A-C,** Values above CDFs indicate the number of genes plotted for each condition in color. Wilcoxon Rank Sum Tests with Continuity Correction and Benjamini-Hochberg False Discovery Rate corrections for multiple testing were used to calculate adjusted *p-values* for the left color being greater than the right color are provided in the upper lefthand corner of the graphs. **(A)** Cumulative distribution function plots comparing log2 fold change between **Soma** or **Axon** specific transcripts as published by Nijssen *et al* (115). Plots are delineated by Sm-site prediction—**Absent** (yellow), **Noncanonical** (green), **Canonical** (purple). **(B)** Cumulative distribution function plots comparing log2 fold change between **Soma** or **Axon** specific transcripts as published by Nijssen *et al* (115). Plots are delineated by whether the transcripts were found to be enriched with Sm-proteins **(Sm-enriched**, purple**)**—log2(f∆) ≥ 0.6, *padj* < 0.05 in the anti-Sm-RIP vs polyA-RNA comparison—or **Un-enriched** (yellow) in the anti-Sm-RIP vs polyA-RNA comparison. **(C)** Cumulative distribution function plot comparing log2 fold change in the anti-Sm-RIP vs polyA-RNA comparison, delineating by RNAs previously found to associate with alpha-COP (**aCOP-enriched**, purple), or not (**Un-enriched**, yellow), as found by Todd *et al* (74). For **D-E**, Values above bars indicate the number of genes contributing to the plots. Numbers in color to the sides of bars indicate the number of genes contributing to the specified group within the bar. **(D)** Proportional bar graph giving a breakdown of types of RNAs associated with alpha-COP and physiologically enriched in anti-Sm-RIP (log2(f∆) ≥ 0.6, *padj* < 0.05). **(E)** Proportional bar graph giving a breakdown of types of Sm-sites predicted in RNAs associated with alpha-COP and physiologically enriched in anti-Sm-RIP (log2(f∆) ≥ 0.6, *padj* < 0.05).
